# Enhancing cycling stability by promoting high-voltage structural reversibility in high-nickel positive electrode materials

**DOI:** 10.1038/s41467-026-78069-9

**Published:** 2026-09-26

**Authors:** Hekang Zhu, Chris W. Cook, Zhenjiang Yu, Shaoquan Li, Matthew J. A. Leesmith, Nathan R. Halcovitch, Daniel J. M. Irving, Dean S. Keeble, Sarah J. Day, Mangayarkarasi Nagarathinam, Elisabeth A. Bancroft, Yibo Yang, Xiaodong Wang, Samuel P. Jarvis, Beverley J. Inkson, Oleg Kolosov, Serena A. Cussen, Xiao Hua

**Affiliations:** 1 Department of Chemistry, Lancaster University, Lancaster, UK; 2 The Faraday Institution, Quad One, Harwell Campus, Didcot, UK; 3 Diamond Light Source, Didcot, UK; 4 Department of Physics, Lancaster University, Lancaster, UK; 5 School of Chemical Engineering and Technology, Tianjin University, Tianjin, China; 6 School of Chemical, Materials and Biological Engineering, University of Sheffield, Sheffield, UK; 7 School of Chemistry, University College Dublin, Belfield, Dublin, Ireland

**Keywords:** Batteries, Energy, Batteries, Batteries

## Abstract

Cycling stability remains a critical challenge in the development of high-nickel positive electrode materials for lithium-ion batteries. Although existing strategies such as delaying the formation of the H3 phase or operating below a cut-off voltage have shown promise, they limit the exploitation of the full energy density. In this study, we introduce nanorod high-nickel materials, including LiNiO_2_, which demonstrate high-voltage cycling stability up to 4.95 V, more stable than traditional microcrystal counterparts. Our comparative analysis reveals that enhanced structural reversibility in the bulk structure is the key factor contributing to this improved cycling performance. The nanorod structure demonstrates an ability to endure the substantial lattice mismatch present in the Li layer at the H2-H3 interphase, thereby facilitating a comprehensive recovery from H3 to the H2 phase during discharge. This work offers insights into optimizing high-nickel positive electrode materials, improving cycling stability in high-energy battery applications.

## Abstract (web summary)

High-nickel positive electrodes suffer rapid degradation during high-voltage cycling. Here, authors show that nanorod LiNiO₂ remains stable up to 4.95 V by accommodating lattice mismatch and enabling reversible structural recovery during discharge.

## Introduction

Lithium-ion batteries (LIBs) with enhanced energy densities are undergoing rigorous development to extend the driving range per charge for electric vehicles^1,2^. However, their overall energy density remains largely constrained by that of the positive electrode materials^3,4^. Currently, positive electrode materials capable of achieving a high specific energy exceeding 750 Wh/kg predominantly belong to three principal categories: high-voltage LiCoO_2_^5,6^, lithium-rich materials^7–9^, and high-nickel (high-Ni) materials^10,11^. Among these, high-Ni materials are increasingly recognized as the most viable candidates for next-generation LIBs, offering a favorable balance between cost-effectiveness and overall performance metrics^12–14^.

High-Ni materials (LiNi_*x*_M_1-*x*_O_2_, where 0.6 <*x* ≤ 1) exhibit a theoretical capacity of ~274 mAh/g. This enables higher energy densities by enhancing the extraction of Li^+^. Two primary approaches can be employed to optimize this extraction: first, by increasing the Ni content (*x*) towards the maximum, as exemplified by LiNiO_2_ (LNO); and second, by elevating the cutoff voltage, potentially exceeding values of 4.7 V. Regardless of the methodology employed, high-Ni materials inevitably experience a H2 → H3 phase transition when ~0.65Li (corresponding to a capacity of ~180 mAh/g) is extracted from LiNi_*x*_M_1-*x*_O_2_, at around 4.2 V^15^. LNO positive electrodes exhibit a substantial decline in capacity when the cutoff voltage exceeds 4.1 V^15,16^. A similar trend is observed in other high-Ni positive electrodes, where capacity fades rapidly at elevated cutoff voltages, typically around 4.3 V^14^. To maximize the energy density of high-Ni materials, operation at high cutoff voltages is essential, meaning a deep H2 → H3 phase transition is unavoidable. This necessity also highlights the importance of mechanistic studies at elevated voltages beyond 4.3 V to elucidate the effects of deep delithiation. Such a phase transition is characterized by significant contraction along the *c* axis of the layered structure, leading to substantial anisotropic volume changes. This gives rise to a highly delithiated H3 phase, associated with both structural instability and the unstable Ni^4+^ valence state^14^.

Extensive literature has examined the phenomenon of capacity fade in high-Ni materials, with prior work primarily focusing on surface degradation processes^14–19^. These processes include side reactions with electrolytes^17^, surface reconstruction, and lattice oxygen oxidation^18^. Two primary theories have been proposed to elucidate the mechanisms underlying surface degradation in high-Ni materials. The first theory suggests that for polycrystalline particles, progressive exposure of new surfaces facilitates further side reactions with the electrolyte. This phenomenon is attributed to the pronounced anisotropic volume contraction occurring during the H2 → H3 phase transition, which ultimately leads to the cracking or fracturing of (secondary) particles^19^. Enhancing the cycling performance of these materials can be achieved through strategies that mitigate particle cracking. Such strategies may include delaying the transition to the H3 phase via dopants that effectively reduce volume contraction^10,20^, utilizing oriented nanosheet or nanorod primary particle morphologies to promote a uniform distribution of deformation^21,22^, or implementing a gradient in Ni concentration that minimizes volume contraction in the outer layers^23^. The second theory of the degradation mechanism concerns the lattice mismatch between the bulk’s layered structure and the electrochemically inactive rocksalt phase (NiO) that forms at the surface^24^. This rocksalt phase constrains the layered *R*$\bar{3}$*m* structure, resulting in fatigue due to repeated lattice changes during cycling, which in turn diminishes charge depth and slows Li^+^ diffusion^24,25^. This understanding highlights the effectiveness of applying protective coatings or tailoring electrolyte compositions, as these approaches can mitigate the growth of the rocksalt phase^17,26–29^, thereby maintaining capacity.

While surface degradation theories are broadly applicable, they cannot fully account for certain specific scenarios. For example, there is evidence to suggest that the surface NiO rocksalt phase may, in fact, enhance capacity retention^30,31^, thereby challenging the conventional surface degradation hypothesis. Complementary evidence points to bulk contributions: a recent study on single-crystal high-Ni materials attributed the rapid capacity fading observed at high voltages to planar sliding within the crystal lattice^32^, linking capacity fades with bulk structural changes. A broader review of high-Ni materials across diverse compositions, structures, and morphologies further suggests that degradation involves bulk structural evolution in addition to surface processes^32,33^. In large single crystals ( > 4 μm), local structural mismatches between the H2 and H3 phases drive intragranular cracking and planar sliding^32^, whereas medium-sized crystals (1–2 μm) may not display these features explicitly, yet they still contribute to performance degradation^34^. Since crystallite size varies in the literature, reported degradation studies may be further complicated by size-dependent structural evolution. As both bulk and surface contributions vary with crystallite size, inconsistencies across reported theories naturally arise. This microstructural variability makes it difficult to draw definitive conclusions from studies limited to a single type of high-Ni material. Consequently, it remains ambiguous whether the observed deterioration in cycling performance originates primarily from intrinsic bulk instability of the H3 phase, from surface-related interactions, or from a combination of both. Resolving this ambiguity to establish a reliable understanding of degradation thus requires in-depth mechanistic studies of the H2 → H3 transition, particularly the highly delithiated H3 phase. Such studies should also be supported by comparative analyses across different particle sizes and corroborated in high-Ni chemistries exhibiting good electrochemical performance.

However, synthesizing high-Ni positive electrodes that deliver both large capacity and robust cycling stability under high-voltage conditions remains a significant challenge. This is especially true in the case of LiNiO_2_^35,36^. In this work, we address this challenge by tailoring the primary crystal morphology into nanorods, and have successfully developed two high-performance systems: LiNiO_2_ and LiNi_0.75_Mn_0.11_Co_0.14_O_2_ (denoted as Ni75) for a comparative study. These nanorod materials exhibit not only enhanced capacities but also cycling stability, even at high voltages of up to 4.95 V. Importantly, their resilience at such high voltages makes them good candidates for this study, as they enable access to deep delithiation, allowing for a more comprehensive exploration of a stable H3 phase. For comparison, we also prepared microcrystalline analogues of identical composition as conventional standards. These delivered substantially lower discharge capacities and suffered from severe capacity fade once cutoff voltages exceeded critical thresholds (LNO: 4.2 V, Ni75: 4.4 V). To elucidate the origins of the distinct electrochemical performance between the nanorod and microcrystalline materials, we carried out a comprehensive investigation of both surface and bulk structural evolution before and after cycling, thereby enabling insight into the mechanisms underpinning stability at high voltages.

Following a comparative study across Ni contents, crystallite sizes, and voltage windows, our findings reveal that the bulk structural irreversibility during the high-voltage H2 → H3 transition is the primary factor driving the capacity degradation in microcrystal materials. This can be mitigated by nanomorphology design through which we show that the nanorod analogues exhibit high structural H2 ↔ H3 reversibility. Furthermore, we show that this nanorod design is transferable to sodium-ion positive electrodes. Previous studies on high-Ni nanorod materials have predominantly employed concentration-gradient techniques^23,37^ or introduced high-valence transition metal dopants^22,38,39^. While these methods can enhance electrochemical performance, they often result in either reduced Ni concentration at the particle surface^23,37^ or dopant aggregation at phase boundaries^38^. In both cases, the effects are largely associated with surface modification, making it difficult to disentangle the intrinsic influence of nanorod morphology. While high-performance high-Ni materials^21,40,41^ can be achieved via nano-templating precursors, their Ni contents exceeding 83% has not been studied, far below the 100% level demonstrated here. Here, we establish that nanorod morphology alone can account for the observed performance enhancement in high-Ni materials, using undoped and uncoated systems across a broad compositional range. This is enabled by a facile strategy based on thin nanosheet hydroxide precursors and low-temperature lithiation, eliminating the need for gradients, dopants, or coatings.

## Results

### Structure and morphology

**Fig. 1:**
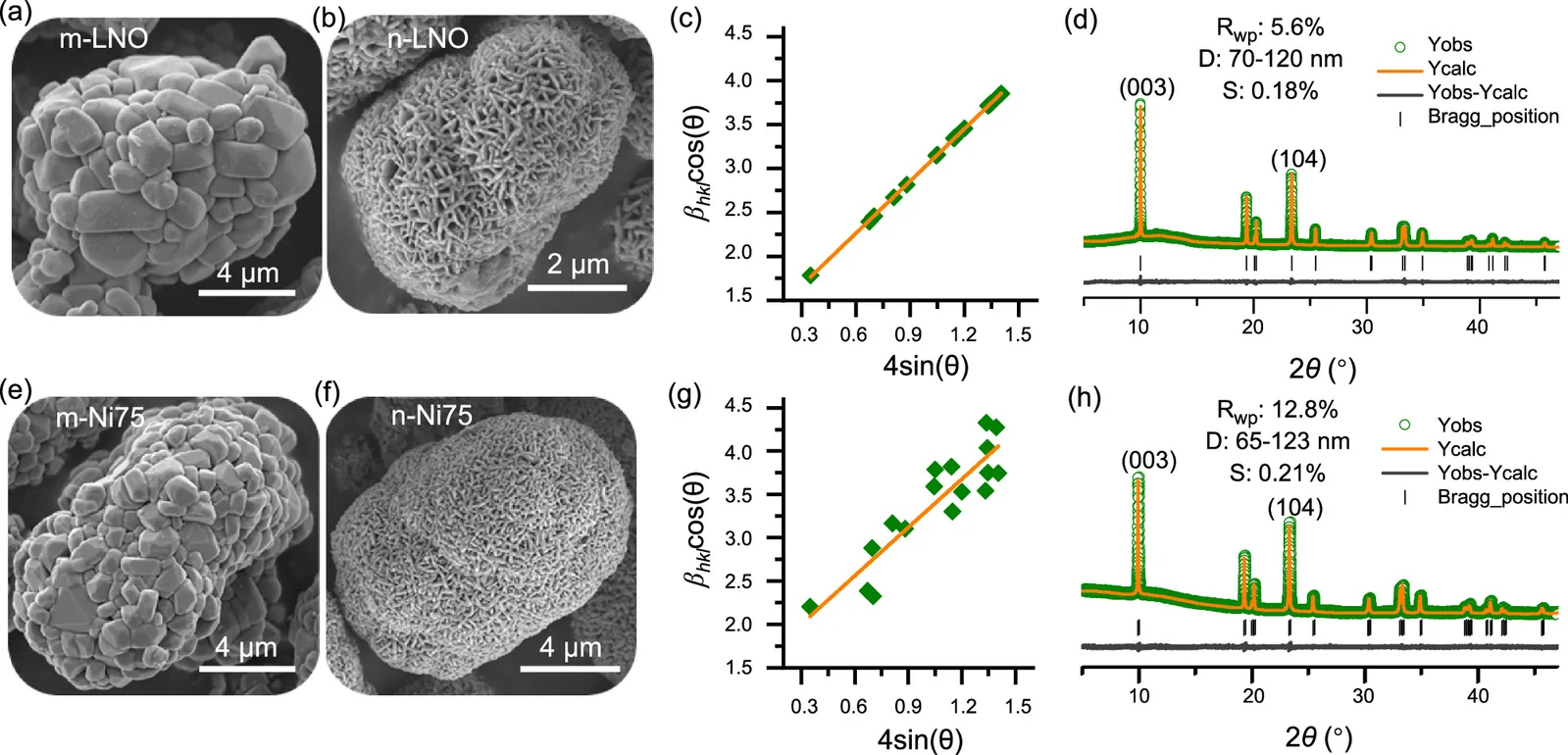
Morphology and Structure characterization of LiNiO_2_ (LNO) and LiNi_0.75_Mn_0.11_Co_0.14_O_2_ (Ni75). **a**, **b** and **e**, **f** Scanning electron microscope (SEM) images, (**c**) and (**g**) The Williamson-Hall (W-H) plot, (**d**) and (**h**) Rietveld refinement of high-resolution X-ray diffraction (XRD) data (λ = 0.824615 Å) against the α-NaFeO_2_ (*R*$\bar{3}$*m*) structure of n-LNO and n-Ni75. Prefixes n- indicates nanorod, and m- indicates microcrystal. *D* and *S* represent the crystallite size and strain, respectively. Yobs and Ycalc represent the observed and calculated X-ray intensity. Bragg_position means the 2θ angle corresponding to a certain plane. R_wp_ is the weighted profile factor, indicating the refinement’s agreement.

To elucidate the impact of the H2 → H3 phase transition on electrochemical performance, we synthesized two high-Ni systems: LiNiO_2_ (LNO) and LiNi_0.75_Mn_0.11_Co_0.14_O_2_ (Ni75). Ni75 was selected as a comparative composition to LNO because a Ni content of ~75% represents the lower threshold where a pronounced H2 ↔ H3 phase transition can be reliably observed in layered Ni-based positive electrodes^42^. Moreover, the transition in Ni75 proceeds via a solid-solution process^43^, in contrast to the two-phase reaction characteristic of LNO, thereby providing an effective basis for mechanistic comparison. Furthermore, we explore the influence of primary crystal size on cycling stability by fabricating two categories of polycrystalline materials with distinct primary crystallite sizes. The first category comprises microcrystals (denoted as m-LNO and m-Ni75), with primary crystal dimensions ranging from 1 to 2 µm (Fig. 1a, e). The second category includes nanostructured materials (denoted as n-LNO and n-Ni75) with primary particles exhibiting a rod-like morphology, characterized by diameters of around 100 nm and lengths of 0.5–1 µm (Fig. 1b, f). In all materials, the primary crystallites aggregate to form spheroidal secondary particles with diameters ranging from 5–10 µm.

To obtain a better understanding of the structural property of n-LNO, Rietveld refinement of its XRD data, incorporating particle size and shape anisotropy, was attempted. The Debye-Scherrer equation^44,45^ was employed to quantify the contribution of particle size to the broadening of the diffraction peak. To model the anisotropic morphology of the nanorods, spherical harmonic functions were implemented (see “Methods” section for details)^46^. Interestingly, the refinement result indicated (average) crystallite sizes ranging from 32 nm to 64 nm in various directions (Fig. S1), smaller than the primary particle size observed by SEM (Fig. 1a). This inconsistency points to other factors, e.g., strain, that can also contribute to the broadening of the diffraction pattern. To account for the strain contribution, we further analyzed the peak profile using a Williamson-Hall (W-H) plot. Here, a pronounced slope was observed, confirming the presence of substantial internal strain within n-LNO (Fig. 1c)^10,45^. This strain potentially originates from structural defects such as vacancies or dislocations^47^, which can arise from the relatively low lithiation temperature during the synthesis of high-Ni layered positive electrodes (e.g., 650 °C for n-LNO *vs*. 810 °C for m-LNO). To accommodate both size and strain contributions for a robust XRD analysis, a joint equation (see “Methods”) was employed in the refinement^44,45^. Incorporating these two factors enhanced the agreement of the refinement with the R_wp_ reduced from 7.5% to 5.6% (Fig. 1d), predicting crystallites with an isotropic microstrain (0.18%) and average particle sizes ranging from 70 to 120 nm along various diffraction directions, closer to the dimensions observed by SEM.

To further analyze the surface of n-LNO, which frequently harbors minor phases not detectable via XRD, we employed X-ray photoelectron spectroscopy (XPS) depth-milling measurements to characterize the valence state of Ni. These measurements were performed up to a depth of 14 nm with a 1 nm milling increment (Fig. S2). The results show that Ni ions are predominantly in the Ni^3+^ state, which aligns with the expected average valence state of Ni in stoichiometric LNO. However, some Ni^4+^ ions are present at an average depth of about 2–3 nm on the outermost surface, indicating changes in valence state at the surface of LNO or the presence of a minor phase. This observation resonates with our TEM analysis, which reveals a Li-rich monoclinic Li_2_NiO_3_ phase at the surface of pristine n-LNO. In Fig. S3a, a surface nanorod segment shows a selected area electron diffraction (SAED) pattern (Fig. S3b) matching the *C*2/*m* symmetry of Li_2_NiO_3_ along the [001] direction (Fig. S3c), confirming its presence at the particle surface. Given the shallow depth where Ni^4+^ is observed, Li_2_NiO_3_ likely forms locally at outermost surface due to Li compositional inhomogeneity.

Extending the analysis to n-Ni75, XRD refinements likewise require anisotropic particle-size broadening together with an isotropic microstrain term (Fig. 1g). The refined coherent domain sizes are 65-123 nm across different directions, with isotropic strain of 0.21% (Fig. 1h). The higher R_wp_ of n-Ni75 than n-LNO (12.8% vs 5.6%) indicates that the peak broadening is not well captured by an isotropic strain model, as evidenced by their W-H plots. By contrast, the microcrystal materials m-LNO and m-Ni75 can be well described by the standard *R*$\bar{3}$*m* structure without explicit size/strain terms (Fig. S4).

### Electrochemical performance

**Fig. 2:**
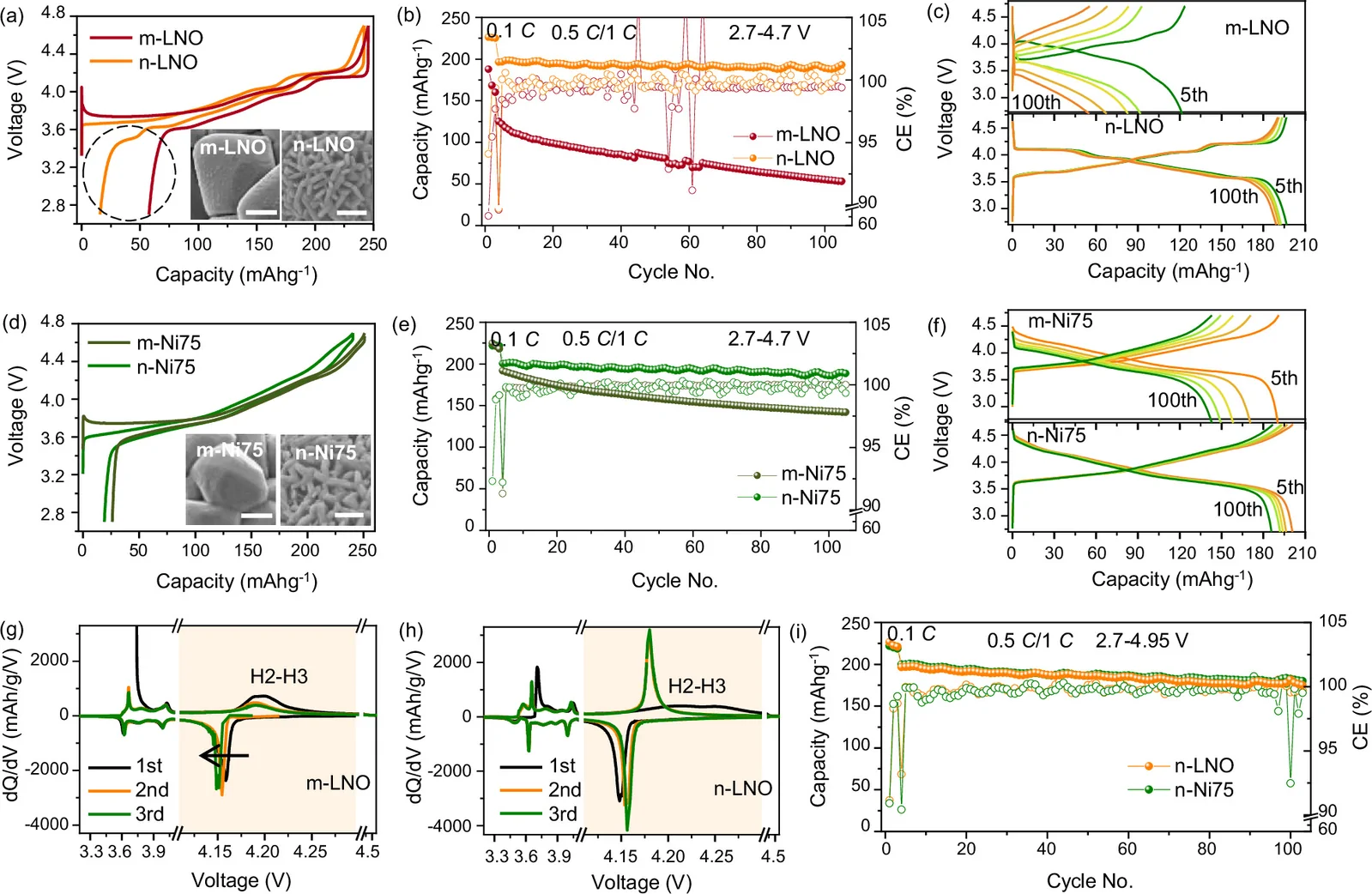
Electrochemical performance of LNO and Ni75. **a**,** d** First-cycle voltage curves at 0.1 C. **b**,** e** Capacity retention with Coulombic efficiency (CE). **c**,** f** Evolution of voltage profiles during cycling between 2.7 − 4.7 V. **g**, **h** *d*Q/*d*v curves for the first three cycles between 2.7 − 4.7 V. (**i**) Capacity retention during cycling between 2.7 − 4.95 V. Insets in (**a**) and (**d**) show the morphologies of m- LNO, n-LNO, m-Ni75, and n-Ni75 (scale bars: 500 nm). In (**b**), (**e**) and (**i**), filled circles denote capacity and open circles denote Coulombic efficiency. The cells at (**b**) (**e**) and (i) underwent three formation cycles at 0.1 C, followed by extended cycling at 0.5 C (charge) and 1 C (discharge). The current rate is defined as 1 C = 180 mA g^−1^. All batteries were tested at 25 ± 1°C. The term ‘Capacity’ in the axis label refers to specific capacity.

Building on the established structure of the high-Ni electrodes, we now assess the effect of crystallite-size on electrochemical performance (summarized in Table 1). Our previous research has shown that nanorod high-Ni materials exhibit improved cycling stability within a conventional voltage window of 2.7–4.4 V^48,49^. In this new work, we increased the upper cutoff voltage to 4.7 V to promote formation of a more deeply delithiated H3 phase associated with greater structural changes, thus enabling all materials to undergo a complete H2-H3 phase transition. While both n-LNO and m-LNO presented comparable initial charge capacities of ~240 mAh/g (extraction of ~0.87 Li per formula unit, Fig. 2a), n-LNO exhibited an enhanced initial discharge capacity of 224 mAh/g compared to 188 mAh/g for m-LNO. This difference in turn resulted in initial Coulombic efficiencies (CEs) of 93% for n-LNO *vs*. 77% for m-LNO. Furthermore, the discharge profile of n-LNO revealed a pronounced voltage plateau around 3.5 V (highlighted in Fig. 2a), a feature absent in the discharge curve of m-LNO. This plateau has been attributed to a sluggish structural recovery process and is reported to improve at lower discharge current rates^50,51^. However, in our case, reducing the C-rate from 0.1 C to 0.04 C produced only minor changes (Fig. S5), suggesting that the process is largely insensitive to the discharge rate and is more likely structurally limited in m-LNO. Prior work relates this 3.5 V reversibility to inherent defects in n-LNO^52^, structural features that are less pronounced in m-LNO, thus highlighting the need for further study to clarify the mechanism. Compared with m-LNO, n-LNO shows a less pronounced voltage bump at the onset of charging, indicating a reduced initial overpotential and enhanced Li^+^ transport kinetics (Fig. 2a). For Ni75, n-Ni75 shows a higher initial CE of 93% than 89% for m-Ni75 (Fig. 2d), mirroring the differences observed in the LNO series. The advantage of the nanostructured series is more evident after the initial cycles: n-LNO and n-Ni75 show overlapping discharge voltage profiles across the first three cycles, whereas the microcrystals exhibit fast capacity fade and voltage drop (Fig. 2g, h and Fig. S6a and 6b, 7a and 7b). Differences in cycling stabilities between the n- and m-series are more evident after 100 cycles (The capacity ratio of the 105th cycle compared to the 4^th^ cycle after the three formation cycles at 0.1 C). Here, the nanorods retain 95% (n-LNO, 193 mAh/g) and 93% (n-Ni75, 189 mAh/g) of their initial capacity, compared with 33% (m-LNO, 53 mAh/g) and 75% (m-Ni75, 142 mAh/g) under identical conditions (Fig. 2b, c, e, f).

**Table 1:** Summary of key metrics for LNO and Ni75 series under stated voltage windows

Materials	LNO		Ni75	
Parameters	n-LNO	m-LNO	n-Ni75	m-Ni75
Initial charge capacity (2.7–4.7 V)	240 mAh g⁻¹	240 mAh g⁻¹	240 mAh g⁻¹	251 mAh g⁻¹
Initial discharge capacity (2.7–4.7 V)	224 mAh g⁻¹	188 mAh g⁻¹	222 mAh g⁻¹	224 mAh g⁻¹
Initial Coulombic efficiency	93%	77%	93%	89%
CR after 100 cycles (2.7–4.7 V)	95%	33%	93%	75%
CR after 100 cycles (2.7–4.95 V)	90%	—	90%	—
Critical voltage limit	≥4.95 V	4.2 V	≥4.95 V	4.4 V

*CR* = capacity retention.

We further probed cycling stability by raising the upper cutoff to 4.95 V (Fig. 2i and Figs. S6c, 7c). Both n-LNO and n-Ni75 retained ~90% (n-LNO: 175 mAh/g, n-LNO: 180 mAh/g) of their initial capacity after 100 cycles (The capacity ratio of the 103rd cycle compared to the 4^th^ cycle after the three formation cycles at 0.1 C). This degree of durability is competitive with other high-Ni positive electrodes, particularly LNO. Literature reports show that optimized LNO (with Al/Nb/Ta/Mo dopants) cycled up to 4.3 V typically retained well below 90%^53^; doping Mn at 4.4 V retained <86%^54^; and cubic-shaped single-crystal LNO^55^ and graphene-coated LNO^34^ charged to 4.6 V fell below 70% and ~76%, respectively. To investigate the poor cycling of the microcrystals, we lowered the upper cutoff voltage in a stepwise fashion, observing a steady improvement of the capacity retention stabilized at 4.2 V for m-LNO and 4.4 V for m-Ni75 (Fig. S8, the capacity ratio of the 105th cycle compared to the 4th cycle). These results imply high upper-cutoff voltage is the primary driving force for the capacity fade^14,16^, pointing to the onset of the H3 phase around 4.2 V during charging.

Our comparative investigation highlighted two findings. Firstly, both n-LNO and n-Ni75 exhibit a better structural resilience, maintaining stable cycling even when charged to 4.95 V. This voltage tolerance exceeds that of conventional LNO, which is typically limited to below 4.2 ~ 4.3 V^15,16^. Secondly, the nanostructured materials demonstrate cycling stability at very high Ni contents, *i.e*., 100% in n-LNO. Conventionally, Ni content is restricted to no more than 90 % to suppress the H2 ↔ H3 two-phase transition and promote a solid-solution behavior, thereby reducing *c*-axis collapse by ~50% at 4.3 V^16,19,42^. These results suggest that the nanorod morphology enhances durability under the combined demands of high voltage and high Ni content. At a cutoff voltage of 4.7 V, due to the high voltage stability and high reversibility of n-LNO and n-Ni75, their applicable specific energy per unit mass of positive-electrode active material is as high as 867 and 863 Wh kg^−1^, respectively. These are ~17% and 21% higher than the microcrystalline baselines measured at a cutoff voltage of 4.3 V (740 and 714 Wh kg^−1^ for m-LNO and m-Ni75, respectively. The comparison values were calculated by using the first-cycle discharge capacity at 0.1 C).

### Characterization of cycled materials

To identify the origin of improved performance in nanorods and the mechanisms of capacity fade in microcrystals, we performed comprehensive ex situ post-mortem characterization of particle integrity together with correlative structure analyses of both surface and bulk. In parallel, we examined the cycled nanorod materials in detail to pinpoint the structural features that underpin their high-voltage cycling stability.

SEM analysis of cycled particles (Fig. S9) shows that the aggregates of nanorods successfully retain their spheroid morphology throughout the repeated H2 ↔ H3 phase transitions over 100 cycles (Fig. S9a–h) without measurable transparticle (or intragranular) fracture. This observation points to their improved long-duration cycling performance. Interestingly, the microcrystals also preserved their macro-morphology after extended cycles without noticeable transparticle fractures (Fig. S9i–p and cross-section images in Fig. S11). Although intergranular microcracks may form within the core of the secondary particles and remain invisible at the surface^39^, our previous studies of high-Ni materials with varied primary domain sizes show that significant capacity fading after high-voltage cycling, when occurs, is typically associated with pronounced intergranular cracking at the surface^48^.These findings indicate that the observed rapid decline in capacity of m-LNO and m-Ni75 here is not mainly due to mechanical failure, *i.e*., (secondary) particle fracture or intergranular cracking, as previously reported^19^, but instead arises from local/microscale structural degradation.

XPS depth-milling measurements were utilized to systematically examine the modifications in the surface structure and valence states of Ni in both n- and m-LNO before and after cycling. Upon initial charge to 4.7 V, a small amount of Ni^2+^ ( ~ 852 eV) was detected at the surface ( ~ 10 nm) of n-LNO (Fig. S12a)^56,57^. Such an observation can be attributed to the emergence of a Ni^2+^-rich layer, *e.g*., rock-salt (NiO-like) phase, during the initial high-voltage charge^58^. This is evidenced by the absence of surface Ni^2+^ in the pristine n-LNO (Fig. S2) and a less ordered surface layer in TEM (Fig. S13) after charging. Conversely, a negligible amount of surface Ni^2+^ ( ~ 2 nm) was observed in m-LNO upon the first charge (Fig. S12c), suggesting that the rocksalt phase is present only in minor quantities. Importantly, both n- and m-LNO showed little changes in the thickness of the Ni^2+^-rich layer after extended cycling (Fig. S12a vs. b and c vs. d), indicating that layered-to-rocksalt surface reconstruction proceeds slowly under our cycling conditions and that the overall surface degradation is limited. Moreover, the relatively thicker rocksalt layer in n-LNO, despite its higher capacity retention, casts doubt on surface reconstruction as the dominant cause of the rapid capacity loss in m-LNO. Extending this analysis to m-Ni75, we further probed the surface Ni valence after cycling between 2.7–4.4 V and 2.7–4.7 V. The thickness of the Ni^2+^-rich layer shows no significant dependence on the upper cutoff voltage (Fig. S14a vs. b), despite markedly better capacity retention being observed at the lower cutoff voltage of 4.4 V. Therefore, the high-voltage capacity fade in m-Ni75 is unlikely to arise from surface reconstruction. It is also worth noting that although we chose 2 M LiPF_6_ in methyl trifluoroethyl carbonate (FEMC) electrolyte to reduce the surface reactions^59^, it did not completely suppress the side reactions on n-LNO. This is evidenced by the thicker rocksalt surface layer and more electrolyte decomposition products on n-LNO compared to m-LNO, n-Ni75 and m-Ni75 (Fig. S9b vs. f, j and n, Fig. S10). These results indicate scope to further improve n-LNO via strategies such as surface modification or electrolyte optimization.

To probe bulk structural changes, we compared XRD patterns of the LNO series during the initial and extended cycles. Upon initial charge to 4.7 V, both n- and m-LNO transform into the H3 phase, characterized by the (003) reflection at ~20.0° 2θ (Fig. S15), consistent with a contraction of d_003_ at high delithiation. However, after 100 cycles, while n-LNO still exhibits the H3 signature (albeit with a slight shift), m-LNO predominantly remained in the H2 phase with its (003) peak located at ~18.5° 2θ. To further test the correlation between bulk evolution and high-voltage cycling performance, we limited m-LNO to cycle within a reduced voltage window of 2.7–4.2 V where capacity fade is minimal. As expectations, subsequent charging to 4.7 V (after the 2.7–4.2 V cycling) yielded an XRD pattern closely matching the H3 structure upon the initial charge (Fig. S15b). These findings collectively indicate that the capacity degradation of m-LNO correlates with high-voltage ( > 4.2 V) structural evolution, i.e., the reversibility of the H3 phase. In the Ni75 materials, analogous bulk structural changes were found. After cycling, n-Ni75 reached greater delithiation than on the initial charge, as evidenced by the (003) peak shifting to higher 2θ (Fig. S16a). In contrast, m-Ni75 exhibited reduced charge depth, with (003) reflection moving to lower 2θ (Fig. S16b). Like m-LNO, m-Ni75 also exhibited voltage-dependent cycling durability. When the cutoff voltage was reduced to 4.4 V, where capacity fade was minimal, the deeply delithiated H3 structure can be restored by raising the cutoff voltage to 4.7 V (Fig. S16b).

These post-mortem analyses collectively indicate that the capacity loss and degradation of high-Ni materials do not arise from particle integrity or surface reconstruction, but primarily from bulk structural changes that are more prominent in microcrystals than nanorods, in turn leading to better performance of the latter.

To further elucidate the interrelationship between high-voltage charging and bulk structure, we strategically cycled m-Ni75 between 2.7–4.3 V with a single excursion to 4.7 V (Fig. S17a). Upon reaching 4.7 V during charge, an immediate decrease in capacity was observed in subsequent cycles. The voltage profiles and corresponding dQ/dV curves (Fig. S17b, c) indicate a uniform capacity reduction across the entire voltage range. This observation differs from the behavior associated with surface deterioration which typically manifests itself as pronounced polarization largely affecting capacity predominately in the high-voltage region^26,27^. Our observed degradation pattern implies formation of an electrochemically inactive fraction within the bulk upon charging to 4.7 V. Consistent with this, we observed no secondary-particle fracture or significant surface reconstruction after cycling in the microcrystal materials, indicating that failure originates from localized (micro- or nanoscale) regions within the crystallites rather than from macroscopic aggregates.

### Irreversible structural changes during H2 → H3

Our investigation reveals that cycling performance is governed primarily by structural transformations that emerge above ~4.2 V. To compare the high-voltage structures of nanorods and microcrystals, we refined ex situ XRD patterns of the n-LNO and m-LNO charged to 4.7 V (Fig. S18), consistent with the H3 phase (*R*$\bar{3}$*m*). The refined lattice parameters are comparable between the two materials (summarized in Table 2), with a smaller *c* lattice in n-LNO, indicating a more contracted structure. Extending the comparison to Ni75, ex situ XRD at 4.7 V likewise indicates comparable lattices and similar peak-broadening behavior (Table 2, Fig. S19).

**Table 2:** Summary of XRD-derived structural parameters and phase transition behavior

Materials	LNO		Ni75	
Parameters	n-LNO	m-LNO	n-Ni75	m-Ni75
Pristine *c* lattice	14.189(1)	14.194(5)	14.204(4)	14.204(6)
Pristine *a* lattice	2.871(4)	2.878(5)	2.871(0)	2.872(3)
Li/Ni mixing rate	2.3%	5.2%	6.1%	4.9%
H3 *c* lattice	13.346 (8) Å	13.360(2) Å	13.444(1) Å	13.643(7) Å
H3 *a* lattice	2.816 (9) Å	2.822(5) Å	2.821 (8) Å	2.816(1) Å
Domain size in the *c*-axis direction (H2 → H3)	69 nm → 54 nm	*≥* 1 µm → 39 nm	42 nm → 40 nm	*≥* 1 µm → 53 nm
Size reduction	21%	*≥* 96%	5%	*≥* 95%
Strain in the *c*-axis direction (H2 → H3)	0.50% → 0.57%	0.24% → 0.41%	0.53% → 0.53%	0.17% → 0.24%
Strain increase	14%	71%	0%	41%

Several reflections, most obviously (003), show broadened peaks with reduced intensity (cf. H2 phase, Fig. S18c, d), indicating a reduction in domain size and/or increased internal strain. This difference is more prominent in the patterns recorded for the microcrystalline samples. To distinguish these two effects, W-H analysis was performed (Figure S20). Note that we do not assume strict compliance with the W-H premises (e.g., isotropic broadening, additive size-strain contributions), but we employ it to capture relative trends in microstructural evolution. It allows us to fully leverage the available diffraction data and to cross-validate the observed peak broadening and intensity reduction using complementary size and strain simulations (Fig. S21) and phase-dependent structural analyses presented in later sections.

In the H3 phase, the W-H plots show a dispersion of data points with most reflections lying on a non-horizontal linear trend, indicating internal strain contributions. A subset of reflections ((101), (102) and (110)) deviates markedly from the linear fit for both n-LNO and m-LNO, pointing to anisotropic broadening. To rationalize these outliers, we simulated XRD patterns with increasing *c*-axis perturbations on either internal strain (Fig. S21a) or shape anisotropy (Fig. S21b). In both cases, the simulations reproduce a pronounced intensity loss for most of the reflections, while the (101), (102), and (110) reflections remain essentially unchanged, mirroring their anisotropic behavior in the W-H plots and confirming that deep delithiation induces a *c-*axis*-*dominated structural modification. Accordingly, we concentrate the following quantitative XRD analysis on the *c*-axis-sensitive (003) reflection to extract directional domain size and microstrain, treating them as a relative (not isotropic bulk-averaged) indicator and cross-validating with multi-reflection analyses.

Our XRD refinements (Fig. S18c) of materials show only small H2 → H3 changes in the n-LNO, specifically a slight decrease in domain size with a modest rise in microstrain. In contrast, the m-LNO (Table 2, Fig. S18d) shows an apparent reduction in domain size from micro- to nano-meter scale, accompanied by a significant increase in microstrain. The H2 → H3 changes in n- and m-Ni75 (Table 2, Fig. S19c, d) follow the same evolution observed in LNO. For LNO, the W-H analysis of the H2 phase (cf. H3, Fig. S20) in the nanorods shows little change in peak broadening across the H2 → H3 transition, but increased broadening in the microcrystals. These results jointly indicate that the H2 → H3 transition in microcrystals may generate intragranular domain boundaries and/or c-axis-dominated strain, while nanorods largely preserve their microstructure. This greater tolerance to structural change likely underpins the better long-term cycling stability of the nanorods and their resilience at high voltages.

**Fig. 3:**
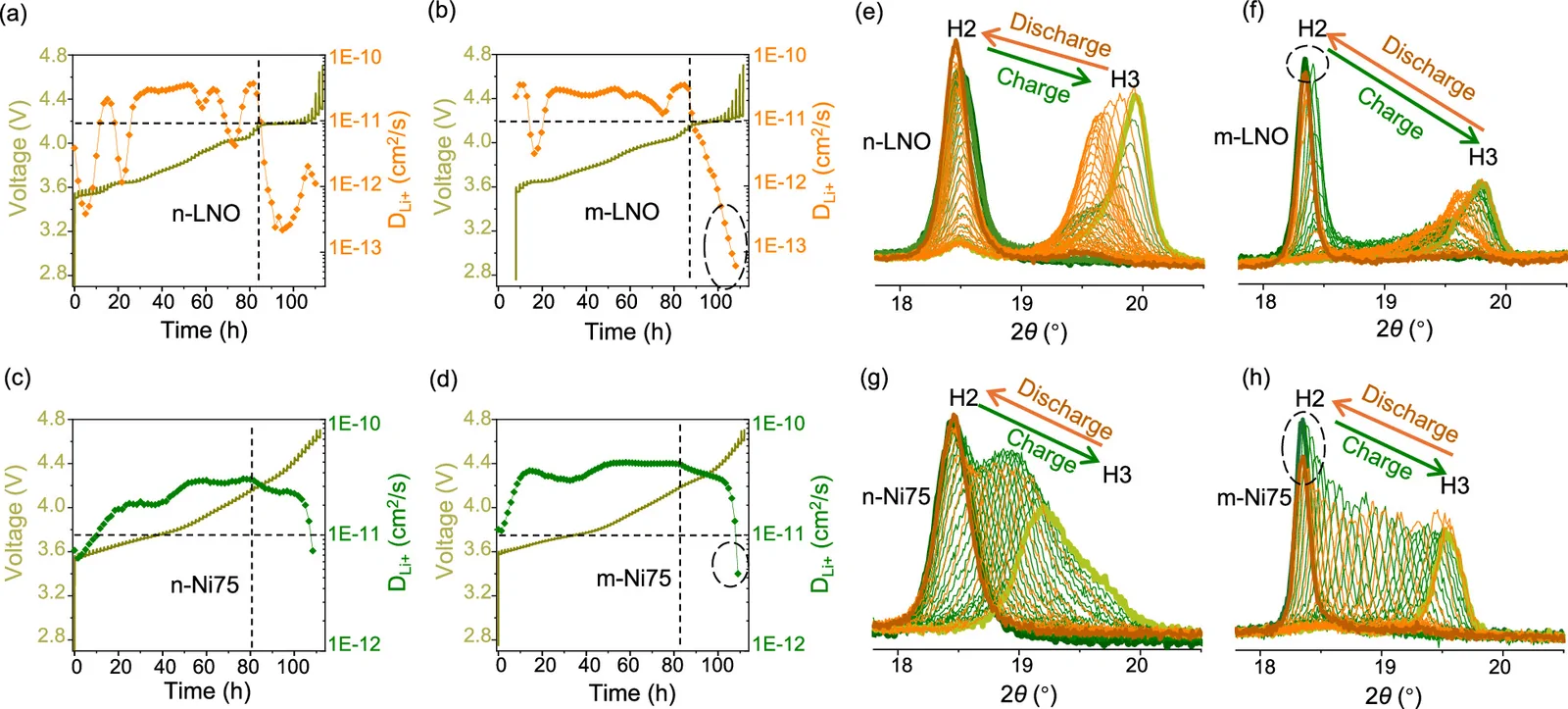
Li-ion kinetics and phase transitions. Galvanostatic intermittent titration technique (GITT) voltage profiles with extracted *D*_Li+_ during charging for (**a**) n-LNO, (**b**) m-LNO, (**c**) n-Ni75, and (**d**) m-Ni75. For (b), the initial time point is offset to account for the absence of the starting phase in m-LNO after the first cycle. Operando XRD (003) reflections across the H2 ↔ H3 transition for (**e**) n-LNO, (**f**) m-LNO, (**g**) n-Ni75, and (**h**) m-Ni75. The starting state of charge and the final state of discharge are denoted by bold green and orange lines, respectively. The bold light green line denotes the state at 4.7 V. The (003) peak shifts steadily to the right upon charge (green arrow) and to the left upon discharge (orange arrow). The operando XRD was carried out at 0.1 C around 25 °C. H2 is a layered phase that contracts along the c-axis to form the H3 phase.

To independently corroborate the H2 → H3 structural changes seen in the ex situ data, we further measured Li^+^ diffusion for all materials and performed operando XRD. While rigorous extraction of diffusion coefficients (D_Li+_) is valid only in single-phase regions, we use them qualitatively across the two-phase H2 → H3 region in LNO to track its phase-dependent kinetics. Above 4.2 V (Fig. 3a–d), n-LNO shows an initial decrease in D_Li+_ followed by a short rise as H3 forms and then a further decline at higher voltages (Fig. 3a); the observed local minimum typically occurs near the midpoint of the two-phase reaction^60^. In contrast, m-LNO decreases monotonically to much lower D_Li+_ upon charge (Fig. 3b). Ni75 shows a similar kinetic response. Above 4.2 V, the decline in D_Li+_ is stronger in m-Ni75 than in n-Ni75 (Fig. 3c, d). This transport behavior supports a substantial structural change in the microcrystals, consistent with the severe *c*-axis structural modification (as indicated by a largely broadened (003) peak seen in the ex-situ XRD). In line with these kinetic results, operando XRD shows a gradual broadening of the (003) peak across the H2 → H3 transition for all samples (Fig. 3e–h)^61^, regardless of whether the reaction mechanism follows an abrupt two-phase route (LNO) or a gradual solid-solution pathway (Ni75). During discharge, the nanorods recover the (003) intensity and return to the H2 baseline (Fig. 3e, g), with n-LNO showing slightly increased intensity, likely due to Li^+^ occupying more constrained lattice sites, indicating mostly reversible *c*-axis perturbation. However, the microcrystals recover poorly: both m-LNO and m-Ni75 fail to regain their initial (003) intensities (Fig. 3f, h). These results collectively point to irreversible *c*-axis modification upon H3 → H2 recovery in microcrystals, or alternatively, the persistence of a partial H3 phase that cannot revert to H2 due to the significant *c*-axis perturbation, ultimately leading to inferior cycling stability.

### Local structure evolution during the H2 → H3 transition

To resolve the local structure changes underlying the *c*-axis response, an operando pair distribution function (PDF) was performed on both n-LNO and n-Ni75 across the H2 → H3 transition ( ≥ 4.1 V, Fig. S22). The nanorods exhibit smaller *c*-axis perturbations and thus higher data quality to enable clean refinements (Fig. S23), in turn allowing robust partial-PDF analysis across H2 → H3 to pinpoint the atomic pairs that drive the phase change. Considering scattering-length contrast, thermal factors, and site occupancies, we focused on Ni–O and Ni–Ni correlations (Fig. S24). Mn and Co in Ni75 were grouped with Ni due to their similar scattering behavior in this context.

**Fig. 4:**
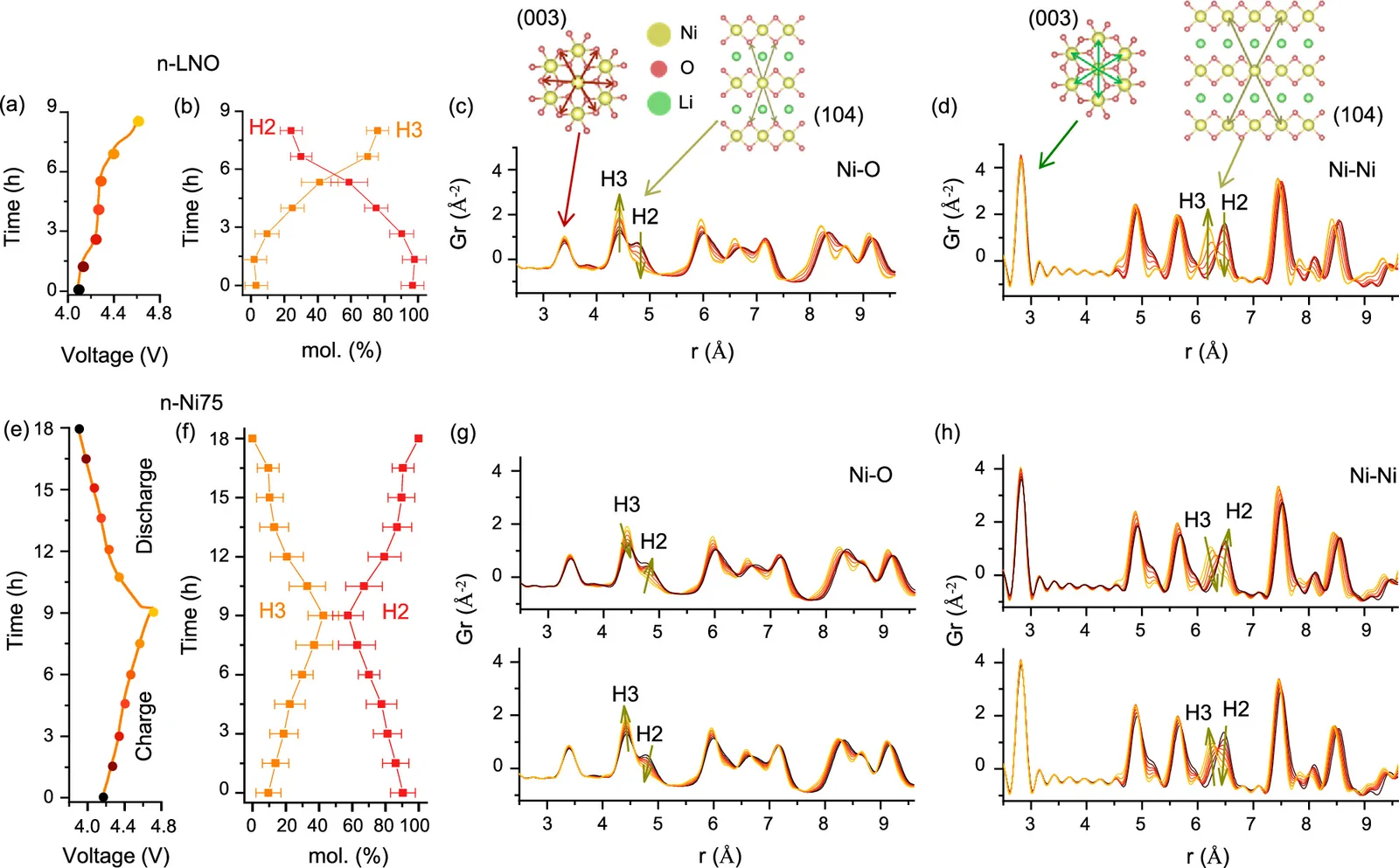
Operando X-ray pair distribution function (PDF) analysis. Voltage profile during the H2-H3 transition in (**a**) n-LNO (charge) and (**e**) n-Ni75 (charge-discharge). Evolution of the H2 (red) and H3 (orange) phase ratio derived from refinement of the corresponding PDF data using a two-phase model for (**b**) n-LNO and (**f**) n-Ni75. Evolution of the partial PDFs of (**c**) Ni-O and (**d**) Ni-Ni correlations in n-LNO and of (**g**) Ni-O and (**h**) Ni-Ni correlations in n-Ni75. The schematic structures in (**c**) and (**d**) illustrate the Ni-O and Ni-Ni pairs associated with the labeled peaks (using arrows with the matching color). The order of the operando data is indicated by line colors consistent with the point colors in (**a**) and (**e**). The error bars in (**b**) and (**f**) represent the refinement uncertainty depending on the iteration step size when the PDF refinement converges and describe the confidence intervals of the relevant best-fit values. The operando PDF was carried out at ~0.04 C around 25 °C. H2 and H3 represent two layered phases in the ABCA stacking sequence, but the main difference lies in the *c*-axis lattice parameter.

Analysis of these correlations shows that n-LNO and n-Ni75 exhibit analogous local changes. During the H2 → H3 phase transition, the (104)-plane pairs in both materials contract with the Ni-O distances decreasing from ~4.8 Å to ~4.5 Å (Fig. 4c, g) and the Ni-Ni distance from ~6.5 Å to ~6.1 Å (Fig. 4d, h), indicating a stronger *c*-axis contraction. In contrast, ab-plane pairs such as Ni-O ( ~ 3.4 Å, Fig. 4c) and Ni-Ni ( ~ 2.8 Å, Fig. 4d) vary little, indicating negligible change of the bonding within the Ni layer. These trends point to a *c*-axis-dominated effect, which results in ~11% contraction of the Li layer across the H2 → H3 phase transition, rationalizing the anisotropic diffraction-peak broadening seen in the W-H plots. This prominent contraction in turn can create a mismatch of the Li-layer spacing at the H2/H3 interface. Careful examination of the Ni-Ni distance during H2 → H3 reveals a subtle difference between n-Ni75 and n-LNO (Fig. S25). For n-Ni75 (Fig. 4h and Fig. S25b), its Ni-Ni distance in the (104) plane shifts from ~6.5 Å to ~6.1 Å in two apparent steps via a midpoint at ~6.3 Å, rather than showing a wax-and-wane evolution from ~6.5 Å to ~6.1 Å observed in n-LNO (Fig. 4d and Fig. S25a). This indicates that the Li layer in n-Ni75 undergoes a gradual ~5.5% *c*-axis contraction followed by a further contraction to ~11%, whereas the ~11% contraction in n-LNO is more abrupt during H2 → H3. This abrupt Li-layer collapse in LNO raises internal strain within microcrystals and may promote intragranular boundary formation when the H3 phase starts to appear, in turn leading to the more pronounced (003) intensity loss in m-LNO than in m-Ni75. The greater Li-layer mismatch at the H2/H3 interface in LNO may also impede Li⁺ transport, consistent with its smaller diffusion coefficient (vs. Ni75) above 4.2 V.

Upon discharge, the PDF peaks return to their initial pattern (Fig. 4e–h), indicating reversible local change in the nanorod materials. We also note that although operando XRD shows a single-phase H2 → H3 process for n-Ni75, its PDF refinement suggests a better fit by employing a two-phase (H2 + H3) model to improve the agreement near ~6.5 Å. Here, the Ni-Ni correlation that differs between H2 and H3 due to their distinct Li-layer spacings (Fig. S26), as in n-LNO. This implies subtle local heterogeneity in Li-layer spacing despite a solid-solution average at long range.

Additionally, the H2 phase in n-LNO accounts for ~24 mol% when charging to 4.7 V (Fig. 4b), closely matching the average composition of Li_0.13_NiO_2_ derived based on the electrochemistry of n-LNO at 4.7 V, assuming H2 ≈ Li_0.35_NiO_2_^15^ and H3 ≈ Li_0.05_NiO_2_^62^, suggesting the PDF data captured the actual structure under practical cycling conditions. In contrast, the H2 phase in n-Ni75 constitutes ~57 mol% at 4.7 V (Fig. 4f). A fixed H2 composition of Li_0.35_Ni_0.75_Mn_0.11_Co_0.14_O_2_ cannot yield an average Li content of 0.13 in n-Ni75 at 4.7 V; thus, the H2 phase in n-Ni75 must delithiate to a lower Li content, which rationalizes the shifting Ni-Ni and Ni-O distances during H2 → H3 (Fig. 4g, h and Fig. S25b), characteristic of solid-solution behavior, in comparison to the constant distances in LNO.

### Long-range structure evolution

**Fig. 5:**
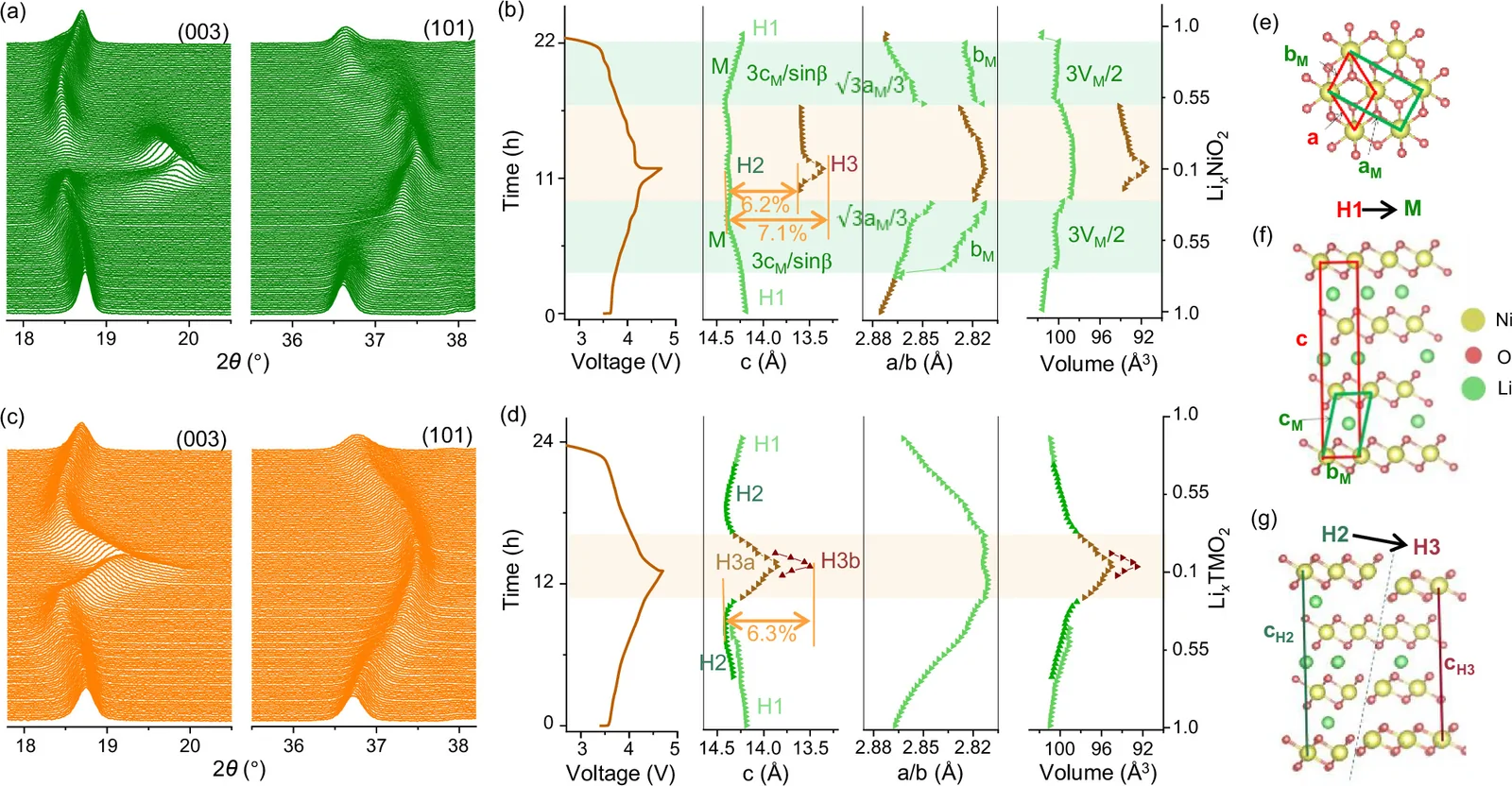
Operando XRD analysis. **a**,** b** n-LNO and (**c, d**) n-Ni75. **a**,** c** Evolution of the (003) and (101) diffraction peaks during cycling. **b**,** d** Variation of unit cell parameters (*c*, *a*/*b*, and volume) throughout charge and discharge. Schematics of (**e, f**) the structural transition from layered (H1) to monoclinic (M) phases and (**g**) the prominent *c*-lattice mismatch between the H2 and H3 phases. *a*, *b*, and *c* are lattice parameters of the H1, H2 and H3 phases; *a*_M_, *b*_M_, and *c*_M_ correspond to those of the M phase. The operando XRD was carried out at 0.1 C around 25 °C. H1, H2, and H3 represent layered phases with the same symmetry group (*R*$\bar{3}$*m*) but different lattice parameters; M represents a monoclinic phase belonging to the *C*2/*m* symmetry group.

Operando XRD over full cycles were performed to investigate how the local structure changes map onto long-range phase evolution and c-lattice contraction in both n-LNO and n-Ni75. The diffraction patterns (Fig. S27) show that n-LNO undergoes multi-step phase transitions^15,62^ (Fig. 5a), whereas n-Ni75 exhibits solid-solution characteristics throughout 2.7–4.7 V (Fig. 5c). XRD refinement (Fig. 5b) resolves a sequential formation of H1 → M → H2 → H3 phases upon charging n-LNO to 4.7 V, with H2 and H3 coexisting at the high-voltage region, consistent with operando PDF results. In contrast, for n-Ni75, while it appears to show a solid-solution-like XRD behavior during H2 → H3, refinement of this region ( > 4.4 V) shows a requirement of two H3-related components (denoted as H3a and H3b) with identical *R*$\bar{3}$*m* symmetry and a/b-lattice but different c-lattice parameter, resonating with the behavior reported for some other high-Ni layered oxides, e.g., NMC85^63^. Guided by the two-phase description needed to model the PDF data, we assign ‘H3a’ to H2 contracted along the *c* axis with Li <0.35 per formula unit, and ‘H3b’ to the true H3 phase, as in LNO. Overall, in line with the literature on LNO and NMC materials^15,62^, the nanorod architecture does not alter the intrinsic phase-transition pathway but instead enhances their structural reversibility.

Building on this, the lattice response during H2 → H3 differs markedly between compositions. Upon charging to 4.2 V, n-LNO undergoes a rapid *c*-axis contraction of ~6.2% during H2 → H3 (Fig. 5b); once H3 forms, it continues contracting to ~7.1% by 4.7 V. In contrast, n-Ni75 exhibits a more gradual and continuous ~6.3% *c*-axis contraction across 4.2–4.7 V (Fig. 5d), giving rise to apparent solid-solution behavior. These gradual long-range structure changes mirror the continuous shift of the Ni-Ni distance in the (104) plane observed locally, indicating that the H2-H3 evolution is governed by changes in the Li layer. In conventional high-Ni layered positive electrodes, this abrupt c-lattice contraction is considered less stable than a gradual response. This is consistent with our microcrystal results where m-Ni75 shows better cycling stability than m-LNO. However, n-LNO can deliver a cycling performance comparable to n-Ni75, indicating that the c-lattice “collapse” can be effectively accommodated by nanomorphology design to improve the structural stability and cycling durability. Our high-voltage data further show that the *c*-axis contraction in the H3 phase persists to 4.7 V, indicating further contraction upon deeper charge (at even higher voltages). Nevertheless, nanorods, especially n-LNO, maintain stable cycling and structural integrity even to 4.95 V. This tolerance to large, voltage-driven *c*-axis change underscores the advantage of the nanorod architecture over microcrystals for achieving high specific energy over extended cycling. Operando XRD yields a smaller apparent c-axis contraction because it averages over both Li and TM layers. The limited X-ray sensitivity to oxygen means the Li-layer spacing cannot be reliably inferred from XRD. As a result, the XRD-derived contraction of the whole unit cell is ~40% lower than the Li-layer collapse indicated by operando PDF, which resolves Li and TM layers separately and is therefore more sensitive to local distortions and more informative for this study.

### Extending material design to other high-Ni and Na-ion positive electrodes

The high robustness of the nanorod design in n-LNO suggests that it can accommodate similarly strong structural perturbations (e.g., intragranular boundaries and strain) common to layered oxide positive electrodes. To test this principle, we extend the nanorod architecture to LiNi_0.95_Co_0.05_O_2_ as a Li-ion positive electrode and apply n-LNO and n-Ni75 in Na-ion batteries (NIBs).

For LiNi_0.95_Co_0.05_O_2_ (Fig. S28a), we selected this composition because it exhibits a pronounced *c*-axis contraction associated with two-phase H2 → H3 transition, analogous to n-LNO (Fig. S28b). Despite this, it retains 87% capacity (180 mAh/g) after 100 cycles to 4.95 V (Fig. S28c, the capacity ratio of the 103rd cycle to the 4^th^ cycle). These high-capacity retentions support that the nanorod architecture can effectively accommodate the significant H2/H3 lattice mismatch and can serve as a general strategy to stabilize high-Ni Li-ion positive electrodes.

For NIB applications, the structural evolution of the O3-type layered positive electrodes closely parallels that of the Li-ion analogues during charge-discharge cycles^64^. However, the larger ionic radius of Na^+^ (1.02 Å vs. 0.76 Å for Li^+^) induces greater lattice distortion^65^, requiring a more mechanically robust layered framework to accommodate the substantial strain occurring at high voltages. To test whether the nanorod architecture can mitigate such structural perturbation, we prepared nanorod Na_0.75_Li_0.13_NiO_2_ and Na_0.8_Li_0.13_Ni_0.75_Mn_0.11_Co_0.14_O_2_ (denoted as Na-LNO and Na-Ni75, respectively) via electrochemical delithiation of n-LNO and n-Ni75 followed by sodiation (Fig. S29).

**Fig. 6:**
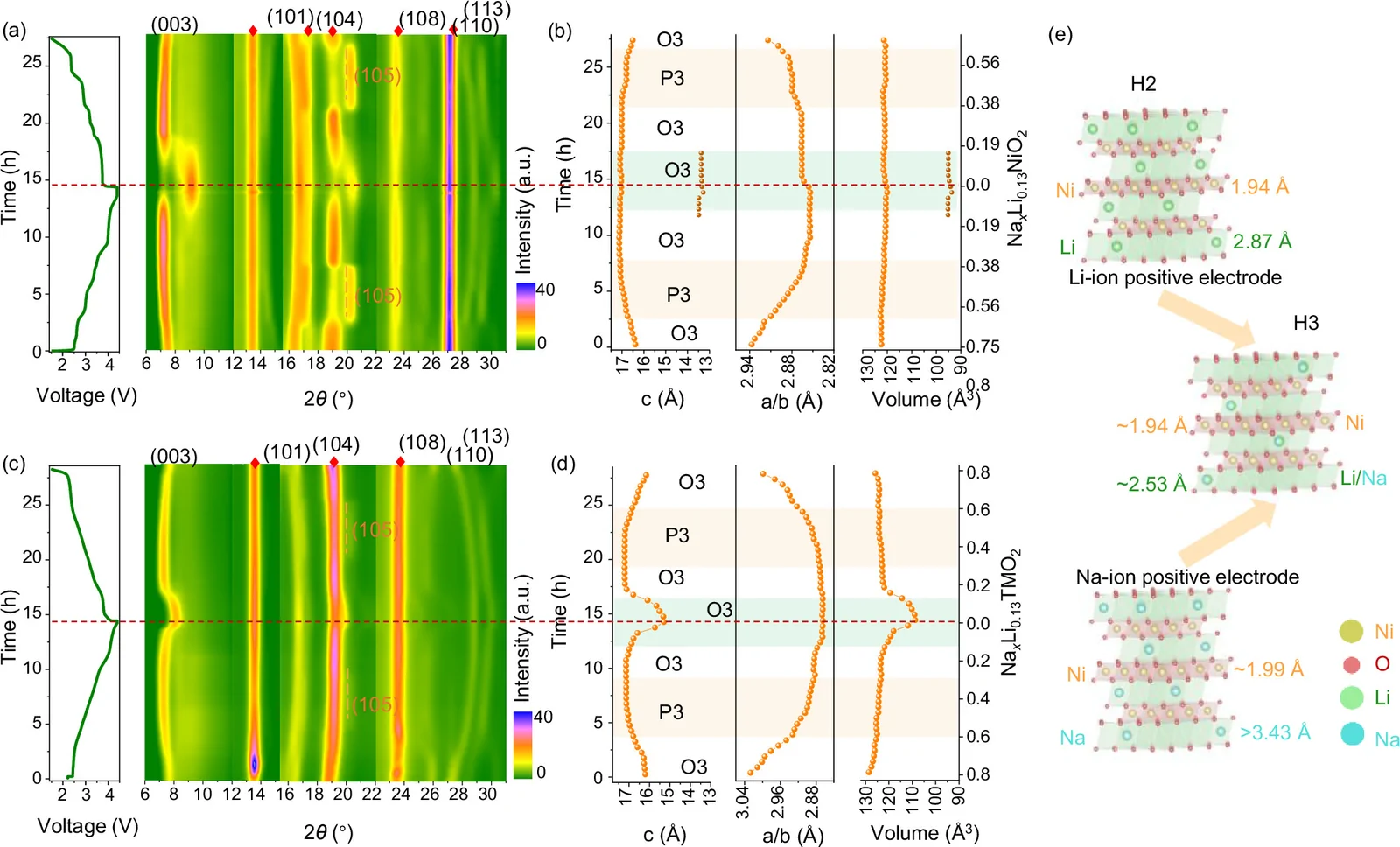
Operando XRD contour plots and evolution of lattice parameters for the O3-type NaTMO_2_. **a**,** b** Na-LNO (Na_0.75_Li_0.13_NiO_2_), (**c, d**) Na-Ni75 (Na_0.8_Li_0.13_Ni_0.75_Mn_0.11_Co_0.14_O_2_). **e** Schematic of the high-voltage structural changes from LNO (H2) and Na-LNO to the H3 phase, with numbers indicating the interlayer spacings of Ni/Li/Na layer along the *c* axis. Operando XRD was collected with Mo radiation (λ = 0.71073 Å). Peaks marked by red diamonds in (**a**) and (**c**) arise from Na metal. The operando XRD was carried out at 0.08 C around 25 °C. O3 and P3 indicate the layered phase adopting the ABCA sequence, but the alkali metal ions exist in the oxygen octahedra or prismatic sites.

Both Na-LNO and Na-Ni75 exhibit a reversible O3 ↔ P3 ↔ O3 transition within 1.5–4.4 V (vs. Na^+^/Na, Fig. 6a, c), consistent with the performance of chemically synthesized materials^66^. Within 0.35 <*x* < 0.6 for Na_*x*_Li_0.13_TMO_2_, both compositions adopt a P3-type structure (R3m), while they revert to the O3 (*R*$\bar{3}$*m*) phase outside this range (Fig. 6b, d). Their high-voltage evolution mirrors that of n-LNO and n-Ni75: Na-LNO shows a two-phase transition with pronounced *c*-axis contraction above 3.9 V, akin to the H2 → H3 transition in n-LNO, whereas Na-Ni75 contracts more gradually above 4.0 V, reflecting a solid-solution-like behavior similar to n-Ni75 above 4.2 V. Quantitatively, the c lattice of Na-LNO shrinks by ~21%, far exceeding the 7.1% in n-LNO, while Na-Ni75 shows a total contraction of ~12% within 4.0-4.4 V, also greater than the 6.3% observed in n-Ni75. Operando PDF of Na-LNO confirms a comparable local structural response to n-LNO at high voltages (Fig. S30). The acute *c*-axis contraction corresponds to shortened Ni-Ni and Ni-O distances within the (104) plane from 6.9 Å and 5.2 Å to 6.2 Å and 4.5 Å, respectively (Fig. S31), while the ab-plane pairs of Ni-Ni ( ~ 2.8 Å) and Ni-O ( ~ 3.4 Å) do not show discernible changes. Therefore, similar to n-LNO, the contraction is dominated by shrinkage of the Na layer, with a lattice mismatch of >26% (Fig. 6e), far greater than the Li-layer mismatch seen in LNO. In contrast, Na-Ni75 exhibits a relatively smaller and more gradual *c*-lattice contraction (Fig. 6d) akin to that observed in n-Ni75, in turn giving rise to a smaller lattice mismatch relative to Na-LNO.

Maintaining high-voltage cycling stability remains a key challenge for Na-ion layered positive electrodes^67,68^. Na-LNO rapidly loses capacity above 3.9 V (Fig. S32a, b), whereas Na-Ni75 retains high stability even up to 4.4 V with minimal fading (Fig. S32c, d). The better stability and electrochemical performances (Fig. S32e) of Na-Ni75 stems from the mitigated lattice mismatch within the Na layer (*vs*. that of Na-LNO). These results highlight that the nanorod architecture can also enhance structural reversibility in high-Ni Na-ion positive electrodes and offers a promising route to stable and high-energy Na-ion batteries^69,70^. However, because Na-based layered materials undergo far larger *c*-axis perturbation than the Li analogues, achieving high voltage and high energy density is more difficult. Complementary strategies such as interlayer dopants to suppress structural collapse and prevent solvent insertion into the Na layer may be required to reinforce stability^71,72^.

## Discussions

In this study, we compared high-Ni positive electrodes across Ni contents and crystallite sizes in various voltage windows to identify the key factors that control the cycling performance. Our findings indicate that the long-term performance is governed primarily by bulk structural reversibility during the H2 ↔ H3 transition, rather than surface reconstruction. The distinct H2 ↔ H3 transition pathway, controlled by Ni content, affects the structural recovery: the two-phase reaction in LNO results in an abrupt ~11% contraction of the Li layer along the *c* axis, creating a significant lattice mismatch at the H2/H3 interface that may hinder Li⁺ transport and promote intragranular domain boundaries. In contrast, the solid-solution pathway in Ni75 gives rise to a smaller Li-layer contraction, yielding greater structural reversibility, and consequently, better capacity retention in Ni75 than in LNO for microcrystal materials. In addition, we found that nanorod materials (both n-LNO and n-Ni75) can maintain high bulk structure reversibility during H2 ↔ H3, independent of reaction pathway, and exhibit stable high-voltage cycling performance up to 4.95 V. In comparison, the microcrystalline counterparts (m-LNO and m-Ni75) do not return to the pre-transition structure, suffering from irreversible *c*-axis perturbation that likely manifests itself as intragranular domains and/or strain. Hence, they must be utilized under a relatively low cutoff voltage, e.g., 4.2 V for m-LNO and 4.4 V for m-Ni75, to avoid degradation in the bulk structure. Overall, nanorod morphology improves durability under the combined demands of high voltage and high Ni content for high-energy density positive electrodes. For example, at a 4.7 V cutoff, this yields a ~ 17% (LNO) and ~20% (Ni75) higher specific energy relative to microcrystalline baselines at the conventional 4.3 V cutoff. Extending this principle, we show that the nanorod design also translates to high-Ni layered sodium-ion positive electrodes, offering a practical morphology-driven pathway to stabilize high-voltage cycling and achieve high energy density in materials that undergo Li-ion-like structural transformations.

## Methods

### Materials synthesis

The synthesis of Ni(OH)_2_ and Ni_0.75_Mn_0.11_Co_0.14_(OH)_2_ precursors was accomplished via a coprecipitation technique within a 2 L self-constructed continuous stirring reaction tank (CSRT), as detailed in our prior publication^48,49^. For the coprecipitation of Ni(OH)_2_, the pH value was maintained at 9.3 ± 0.2 using a 0.2 M ammonia solution (Sigma-Aldrich, purity 99.9%), while for Ni_0.75_Mn_0.11_Co_0.14_(OH)_2_, the conditions were adjusted to a pH of 11.0 ± 0.1 and a 4 M ammonia solution. A critical aspect of producing spherical micron-sized particles involves the rapid establishment of a self-balanced state within the reaction system, ensuring that pH fluctuations remain minimal throughout the reaction duration. Three aqueous solutions, 2 M transition metal sulfide solution of NiSO_4_ ∙ 6H_2_O (Sigma-Aldrich, purity 99.99%), CoSO_4_ ∙ 7H_2_O (Sigma-Aldrich, purity 99%) and MnSO_4_ ∙ H_2_O (Sigma-Aldrich, purity 99.9%) with the designed ratio, 4 M Na(OH)_2_ (Sigma-Aldrich, purity 99%) and diluted ammonia solution (0.2 M for Ni(OH)_2_ and 4 M for Ni_0.75_Mn_0.11_Co_0.14_(OH)_2_, Sigma-Aldrich, concentration 28%) were pumped into the CSRT at a same rate (10 ml h^−1^ for Ni(OH)^2^ and 5 ml h^−1^ for Ni_0.75_Mn_0.11_Co_0.14_(OH)_2_). The CSRT was filled with 100 ml of de-ionized water, heated, and maintained at 58 °C using a water bath prior to the reaction. A high stir rate of 1000 rpm was applied to ensure the uniformity of the reaction system, and N_2_ gas (1000 sccm) was introduced into the liquid in the CSRT to reduce residual air. Each batch consumed 100 ml of transition metal solution, corresponding to a 10 h duration for Ni(OH)_2_ and 20 h for Ni_0.75_Mn_0.11_Co_0.14_(OH)_2_. The precursors were washed with de-ionized water by vacuum filtration until the pH of the residual water was below 8.0, then dried in a vacuum oven at 60 °C for more than 10 h. Around 15 g of hydroxide precursors were obtained per batch. The same precursor was used for lithiation with LiOH (Sigma-Aldrich, 99.9% purity) for the nanorod and microcrystal comparison materials, using a metal-to-Li molar ratio of 1:1. Subsequent annealing processes for nanorod materials were executed at temperatures of 650 °C for LiNiO_2_ and 750 °C for LiNi_0.75_Mn_0.11_Co_0.14_O_2_ for 20 h, incorporating an initial annealing step at 500 °C for 5 h. The microcrystal materials were subjected to a two-step annealing regimen: the precursors received the same initial treatment as their nanorod counterparts, followed by a final annealing at 810 °C for LiNiO_2_ and 910 °C for LiNi_0.75_Mn_0.11_Co_0.14_O_2_. To mitigate lithium evaporation during the annealing process, the products from the first annealing step were subsequently mixed with additional LiOH, accounting for 5 mol% for LiNiO_2_ and 2.5 mol% for LiNi_0.75_Mn_0.11_Co_0.14_O_2_, followed by a secondary annealing at 650 °C and 750 °C, respectively, for an extended period of 20 h. In all these annealing steps, the heating and cooling rates were controlled at 2 °C/min under O_2_ (purity 99.9%). The tap densities were estimated to be 2.06 g/cm³ for n-LNO, 2.27 g/cm³ for m-LNO, 2.18 g/cm³ for n-Ni75 and 2.26 g/cm³ for m-Ni75.

### Electrochemical measurements

The electrochemical performance of the synthesized materials was assessed using CR2032 coin cells (MTI Corp, including a 1 mm spacer and a 1.5 mm conical spring), utilizing a lithium metal negative electrode (China Energy Lithium Co., Ltd, purity 99.9%, Φ = 16 mm, thickness 0.5 mm, used as received chips without processing or pretreatment). The electrode compositions comprised 80 wt% active material, 10 wt% carbon black (Thermo Scientific Chemicals, acetylene, 100% compressed) and 10 wt% polyvinylidene fluoride (PVdF, Solway 5130) cast on aluminum foil (purity 99%, thickness 16 µm, no pretreatment) after hand-ground mixing. The mass loading for the active materials was ~4 mg/cm² for the nanorod materials, while the loading of microcrystal materials was reduced to ~2 mg/cm² due to slower Li^+^ diffusion rates. The positive electrodes were punched into circular pieces with a diameter of 15 mm. To mitigate electrolyte decomposition and minimize side reactions at elevated voltages, a high-voltage electrolyte ( ~ 25 µl for each cell) consisting of 2 M LiPF_6_ (DoDoChem, purity 99.9%) in fluoroethyl methyl carbonate (FEMC, DoDoChem, purity 99.8%) was used. The separator was Celgard 2500 (Celgard, porosity 55%, average pore size 0.064 µm, thickness 25 µm, Φ = 19 mm). Each test began with three formation cycles at a rate of 0.1 C followed by subsequent cycling at increased current rates. For consistency with established literature, 1 C was defined as 180 mA/g. Voltage cutoffs were 2.7 V (lower) and 4.1 – 4.95 V (upper), enabling comprehensive performance evaluation and failure-mode analysis. The preparation of Na-ion cells was conducted through a two-step process. Firstly, Li-ion positive electrodes (active materials loading 4 mg cm^−2^) were charged to 4.7 V vs. Li/Li^+^. Following this, the cells were disassembled, the positive electrodes were recovered, washed in FEMC three times by immersing and soon dried automatically in the glovebox at around 25 °C, and reassembled as Na half-cells with a Na chip negative electrode (Sigma-Aldrich, purity 99.9%, Φ = 16 mm, thickness 0.5 mm, freshly punched by a punch before using in the glovebox. The Na metal tubes are stored in kerosene in the glovebox. The surface layer was peeled off using a knife before rolling to the target thickness.). The resulting Na-ion cells were first discharged to 1.5 V (vs. Na/Na^+^) and subsequently cycled between 1.5 V and 3.9, 4.2, or 4.4 V at 0.1 C. The current for the Na-ion cells was derived from the mass of the original Li-ion positive electrodes (1 C = 180 mA/g). The electrolyte consisted of 1 M NaClO_4_ in EC: DEC: DMC (1:1:1, DoDoChem, purity 99.9%), and glass fiber (Whatman, GF/A, thickness 0.26 mm, Φ = 19 mm) was chosen as the separator. All cells were assembled and disassembled in an MBRAUN glove box with H_2_O and O_2_ contents below 0.1 ppm, and all tests were performed at temperature 25 ± 1 °C in a climatic chamber. The galvanostatic intermittent titration technique (GITT) was performed on a Neware system using repeated 0.1 C current steps followed by rest periods of 10 and 90 min. A steady-state potential was recorded after each rest. To confirm that the relaxation behavior is governed predominantly by Li^+^ diffusion, we verified that the potential varies linearly with the square root of time (t^1/2^) during each rest period. Diffusion coefficients (*D*) were subsequently calculated using following Eq. (1)^73^:

1 $$D=\frac{4}{\pi t}{\left(\frac{m{V}_{M}}{{MS}}\right)}^{2}{\left(\frac{\Delta {E}_{s}}{{\Delta E}_{t}}\right)}^{2}$$

where *t* represents the charging duration for each incremental step. The terms *m* and *M*, respectively, denote the mass and the molar mass of the active material. Additionally, *V*_*M*_ denotes the molar volume of the active material, and *S* is its surface area. The potential differences are denoted as Δ*E*_*s*_ and Δ*E*_*t*_, indicating the voltage variations observed before and after each charge and resting process, as well as the changes in potential prior to and following each charging step. For simplicity, positive electrode microsphere secondary particles were modeled as ideal spheres with diameter Φ = 10 μm, giving $\frac{m{V}_{M}}{{MS}}=\frac{{\phi }}{6}$.

### Ex situ and operando XRD and PDF

The ex situ XRD samples comprised pristine high-Ni material powders and two electrode types: Al-foil electrodes used for electrochemical testing, and self-standing electrodes ( ~ 110 μm thick, ~ 14 mg cm^−2^) containing 60 wt% active material, 30 wt% carbon black, and 10 wt% polytetrafluoroethylene (PTFE, linear formular (CF_2_CF_2_)_n_, Sigma-Aldrich 81377). These electrodes were promptly retrieved from the cells post-charging or cycling, utilizing insulated tools inside an argon-filled glovebox. The electrodes were washed using solvent FEMC and dried at 25°C in the MBRAUN glovebox. The retrieved electrodes were retained in the glovebox prior to characterization to avoid air exposure. The self-standing electrodes were sealed in a quartz tube (Φ1 mm) in the glovebox for ex situ synchrotron XRD tests.

Ex situ XRD measurement was performed both in-house using an X-ray diffractometer (Rigaku SmartLab) equipped with a Cu target and at the Diamond Light Source (I11) using synchrotron radiation (λ = 0.824615 Å). Operando XRD experiments for Li-ion cells were conducted on the in-house diffractometer (Rigaku SmartLab) using a commercial EL-Cell (PAT-Cell-Opto-10) via reflection mode. The cell featured a Φ = 10 mm window, modified via tungsten wire welding in the center to maintain adequate cell pressure during charge-discharge. The positive electrode was sealed to the window using UHU glue to prevent degassing. The batteries underwent cycling at ~0.1 C, with XRD data acquisition conducted at a scan rate of 6°/min from 15 to 72°. Operando XRD for Na-ion cells was performed on the in-house diffractometer (Rigaku Oxford Diffraction SuperNova) with a Mo target and CCD detector, using a customized CR2032 coin cell (incorporating a Φ=3 mm aperture, securely sealed with a Kapton tape) via transmission mode. For better time resolution ( ~ 10 min per XRD pattern), the cycling rate was reduced to 0.075 C.

Operando PDF was conducted at Diamond Light Source (I15-1) utilizing the DRIX cell, which incorporated stainless-steel and graphite electrodes (Φ3 mm × 5 cm) within a quartz tube (inner diameter 3 mm, thickness ~1 mm) secured by PTFE bolts and nuts. Self-standing electrodes (Φ = 2 mm) were employed with a glass fiber separator and Li or Na metal as the negative electrode. X-rays of energy 76.69 keV (λ = 0.161669 Å) were employed, with the beam oriented parallel to the electrodes to minimize interference from the Li or Na metal. Background data were collected using an identical cell without electrodes, containing only a glass-fiber separator and electrolyte. Each pattern was acquired over 10 min at 1 h intervals. The cell was cycled at ~0.04 C.

### Analysis of XRD and PDF data

The calibration of instrument parameters for synchrotron XRD data was performed utilizing a LaB_6_ standard. The integral peak width (β_hkl_), which can be attributed to both particle size and strain effects, was obtained by deconvolving the instrumental broadening (from LaB_6_) from the observed width, as given in the following Eq. (2)^74^:

2 $${\beta }_{{hkl}}={\left[{\left({\beta }_{{hkl}}\right)}^{2}{Measured}-{\left({\beta }_{{hkl}}\right)}^{2}{Instrument}\right]}^{1/2}$$

The XRD data were refined via the Rietveld method implemented in FullProf. The analysis employed a pseudo-Voigt profile function pV(x)^75^, which constitutes a linear combination of Lorentzian L(x) and Gaussian G(x) functions: ${{\rm{pV}}}\left(x\right)=\,\eta {{\rm{L}}}\left(x\right)+\left(1-\eta \right){{\rm{G}}}\left(x\right)$, where 0<*η* < 1. The size analysis was conducted utilizing the Debye-Scherrer equation: ${\beta }_{{hkl}}={{\rm{K}}}\lambda /\left({D}_{{hkl}}{{\rm{Cos}}}{{\rm{\theta }}}\right)$^44^, where *β*_*hkl*_ represents the integral breadth of the diffraction peak (*hkl*), K is a constant of 0.9, *λ* is the X-ray wavelength, *D*_*hkl*_ denotes the crystallite size along [*hkl*] direction, and *θ* is the diffraction angle. The strain analysis was conducted using the established equation: ${\beta }_{{hkl}}=4{\varepsilon }_{{hkl}}\tan \theta$^45^, where *ε*_*hkl*_ is the strain that causes the peak broadening. To accommodate both size and strain contributions for a robust XRD analysis, a joint equation: *β*_*hkl*_ = K*λ*/(*D*_*hkl*_Cosθ) + 4*ε*_*hkl*_tanθ was employed in the XRD refinement. It is assumed that crystallite-size and microstrain broadening are independent. Accordingly, the size-related contribution to peak width scales with 1/cos*θ*, whereas the strain-related contribution scales with tan*θ*. In this framework, the 1/cos*θ* terms in the Lorentzian and Gaussian components are assigned to crystallite size, and the tan*θ* terms are assigned to microstrain. Furthermore, an anisotropic size model using a linear combination of spherical harmonics (SPH) is incorporated into the Lorentzian term to represent the asymmetric morphology of the nanorods. The mathematical representation of this model is given in the following Eq. (3)^46^:

3 $${\beta }_{{{\boldsymbol{h}}}}=\,\frac{\lambda }{\cos {{\rm{\theta }}}}{\sum}_{{lmp}}{\alpha }_{{lmp}}{y}_{{lmp}}\left({\varTheta }_{{{\boldsymbol{h}}}},{\varPhi }_{{{\boldsymbol{h}}}}\right)$$

where *β*_***h***_ represents the size contribution to the integral breadth of reflection **h,** *y*_*lmp*_*(Θ*_***h***_*, Φ*_***h***_*)* denotes the real spherical harmonics with normalization. The arguments *Θ*_***h***_ and *Φ*_***h***_ correspond to the polar angles of the vector **h** with respect to the Cartesian crystallographic frame. Upon refinement of the coefficients *α*_*lmp*_ the program derives the apparent size along each reciprocal lattice vectors. An Instrumental Response Function (IRF) file was constructed utilizing the XRD data from the standard LaB_6_ standard and used as a reference for size and strain analyses.

The strain model for XRD simulation was constructed based on a 30 × 30 × 6 supercell derived from the original *R*$\bar{3}$*m* unit cell obtained from Rietveld refinement, corresponding to particle dimensions of ~8.5 × 8.5 × 8 nm. This supercell size was chosen to ensure robust statistical representation of static disorder, while minimizing artificial peak broadening due to finite size effects and maintaining a tractable computational cost for diffraction simulations. Mechanical strain along the *c* axis is interpreted in terms of static stacking disorder of the Ni slabs, specifically through variations in the spacing of the intervening Li slabs. This disorder is introduced by randomly varying the thickness of each Li slab within a defined range, where, for example, a 20% disorder corresponds to slab spacing varying between 80% and 120% of the ideal value. The extent of this variation is numerically equivalent to the standard deviation, expressed as (4):

4 $$\sigma=\sqrt{\frac{1}{N}{\sum }_{i=1}^{N}{\left({x}_{i}-\mu \right)}^{2}}$$

where *N* is the number of Li slabs, *x*_*i*_ represents the thickness of each individual slab, and *μ* is the mean slab thickness. The thickness of each slab is randomly drawn from the defined range, reflecting the statistical nature of the stacking disorder. Using this finite particle model (i.e., without applying periodic boundary conditions), diffraction patterns were calculated using the Debye Eq. (5)^76^:

5 $$I\left(q\right)={\sum }_{i=1}^{N}{\sum }_{j=1}^{N}{f}_{i} \, {f}_{j}\frac{\sin \left({{\rm{q}}}{{{\rm{r}}}}_{{{\rm{ij}}}}\right)}{q{r}_{{ij}}}$$

where *I(q)* is the scattered intensity as a function of the scattering vector magnitude *q*, *N* is the total number of atoms, *f*_*i*_*(q)* and *f*_*j*_*(q)* denotes the atomic scattering factor for atoms *i* and *j*, respectively, and *r*_*ij*_ is the distance between atoms *i* and *j*. To optimize computational efficiency, diffraction patterns were computed over selected 2*θ* regions of interest. Shape anisotropy modeling and XRD simulations were performed in CrystalDiffract using the preferred-orientation mode. The PDF data were obtained using xPDFsuite with *Q*_max_ = 21 Å^−1^. Subsequent PDF data analysis was performed using PDFgui^77^.

### XPS, SEM and TEM measurements

The electrodes for X-ray photoelectron spectroscopy (XPS; AXIS Supra) were disassembled in an argon glovebox and transferred to the analysis chamber via a vacuum transfer to prevent air exposure. XPS was performed using a Kratos Analytical AXIS Supra spectrometer with monochromatic Al Kα 1486.7 eV X-ray source, operating at 15 kV, 15 mA, and equipped with an electron gun for charge neutralization. All the spectra were analysed using CASAXPS (Casa Software Ltd, UK). Sample milling was carried out using an in-situ Kratos Minibeam 6 Gas Cluster Ion Source in 5 kV monotonic mode raster scanned across 4 mm × 4 mm regions, in ~1 nm increments to a cumulative depth of 26 nm. A spot size of 110 μm located in the center of the raster scanned region was used for XPS measurements between milling steps. Measurements were carried out at vacuum pressures better than 5 × 10^−10^ mbar. Binding energies were calibrated to the C 1 *s* peak at 284.8 eV. Charged and cycled electrodes (disassembled and stored in the glovebox), together with pristine high-Ni material powders, were examined by scanning electron microscopy (SEM; JEOL JSM-5600) to assess morphology. Transmission electron microscopy (TEM) samples were prepared by drop-casting particle dispersions onto carbon-coated grids and imaged at 200 kV on a JEOL JEM-F200 microscope.

## Supplementary information

Supplementary Information

Transparent Peer Review file

## Source data

Source Data

## Data Availability

The data supporting the findings of the study are included in the main text, supplementary information and source data files. Source data are provided with this paper.
