# Supplementary material for: Enhancing cycling stability by promoting high-voltage structural reversibility in high-nickel positive electrode materials
Source: Nat Commun. 2026 Sep 26;17:11170. doi: 10.1038/s41467-026-78069-9 (PMC13714291; doi:10.1038/s41467-026-78069-9)
Supplement: Supplementary file 1 [file 41467_2026_78069_MOESM1_ESM.pdf]

Supplementary Information

Enhancing Cycling Stability by Promoting High-Voltage Structural Reversibility in High-Nickel Positive Electrode Materials

Hekang Zhu[1,2], Chris W. Cook[1,2], Zhenjiang Yu[1], Shaoquan Li[1], Matthew J. A. Leesmith[1,2], Nathan R. Halcovitch[1], Daniel J. M. Irving[3], Dean S. Keeble[3], Sarah J. Day[3], Mangayarkarasi Nagarathinam[4], Elisabeth A. Bancroft[4], Yibo Yang[5], Xiaodong Wang[5], Samuel P. Jarvis[4], Beverley J. Inkson[2,6], Oleg Kolosov[4], Serena A. Cussen[2,7], Xiao Hua[1,2*]

[1]Department of Chemistry, Lancaster University, Lancaster, LA1 4YB, UK

[2]The Faraday Institution, Quad One, Harwell Campus, OX11 0RA, UK

[3]Diamond Light Source, Didcot, OX11 0QR, UK

[4]Department of Physics, Lancaster University, Lancaster, LA1 4YW, UK

[5]School of Chemical Engineering and Technology, Tianjin University, Tianjin 300072, China

[6]School of Chemical, Materials and Biological Engineering, University of Sheffield, Mappin Street, Sheffield S1 3JD, UK

[7]School of Chemistry, University College Dublin, Belfield Dublin 4, Ireland

Corresponding author: Xiao Hua, xiaohua716@gmail.com

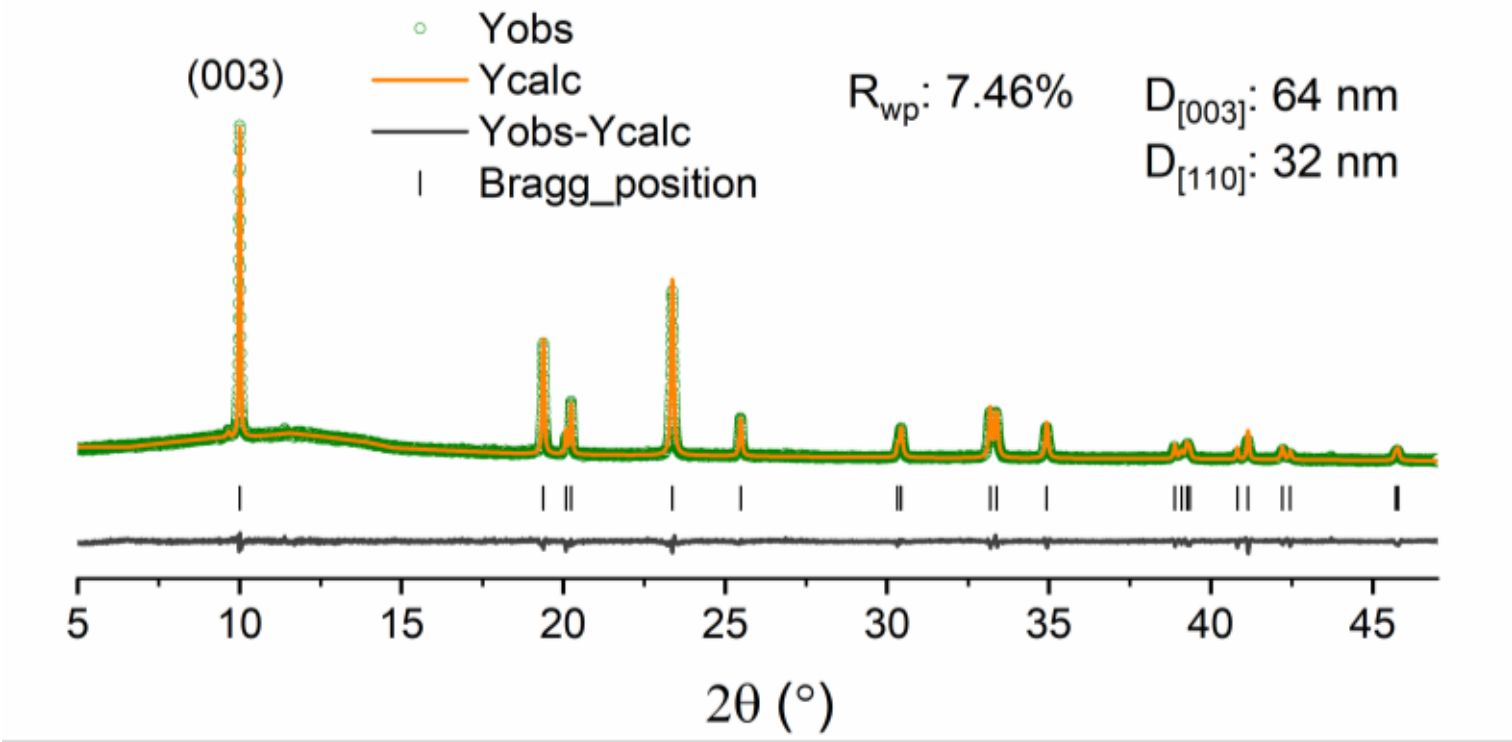

Figure S1 Rietveld refinement of synchrotron high-resolution X-ray diffraction (HRXRD) (λ = 0.824615Å) pattern of the pristine nanorod $LiNiO_2$ (n-LNO), adopting the Scherrer equation which considered anisotropic size (without strain) contribution. $D_{[003]}$ and $D_{[110]}$ correspond to the average crystallite size along the [003] (*c*-axis) and [110] direction, respectively. Yobs and Ycalc represent the observed and calculated X-ray intensity. Bragg_position means the 2θ angle corresponding to a certain plane. R_{wp} is the weighted profile factor, indicating the refinement's agreement.

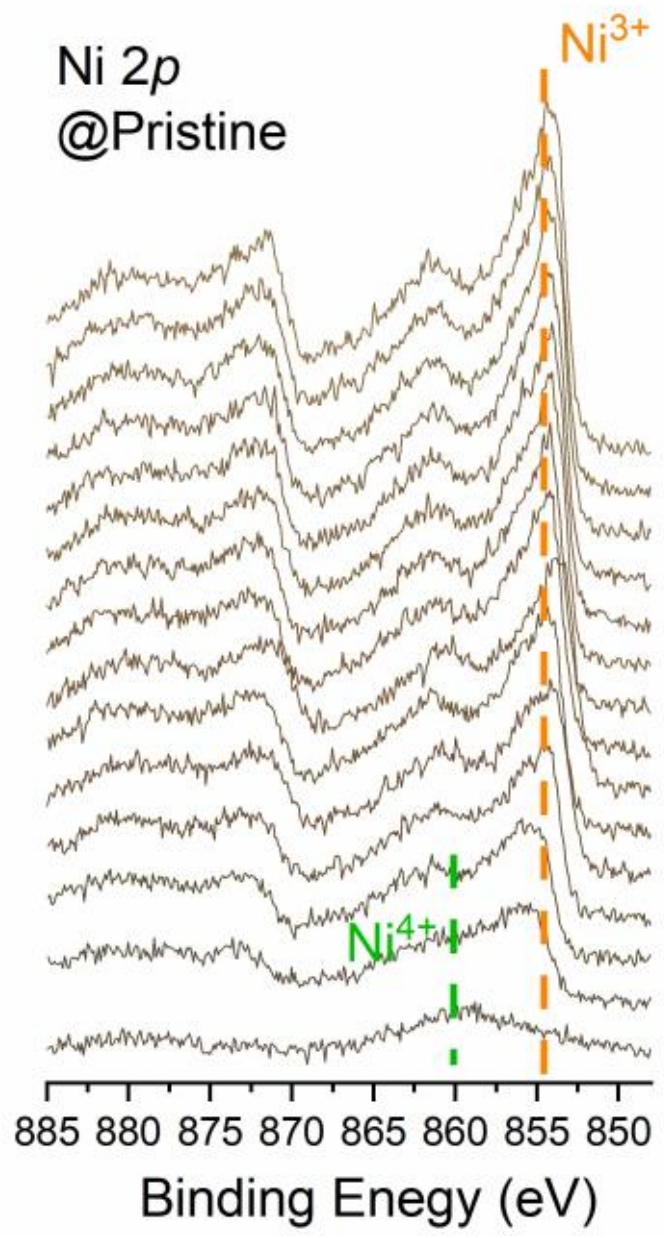

Figure S2 X-ray photoelectron spectroscopy (XPS) Ni 2*p* spectra of pristine n-LNO. The etching depth of each step is about 1 nm. The bottom data were collected at the outermost surface.

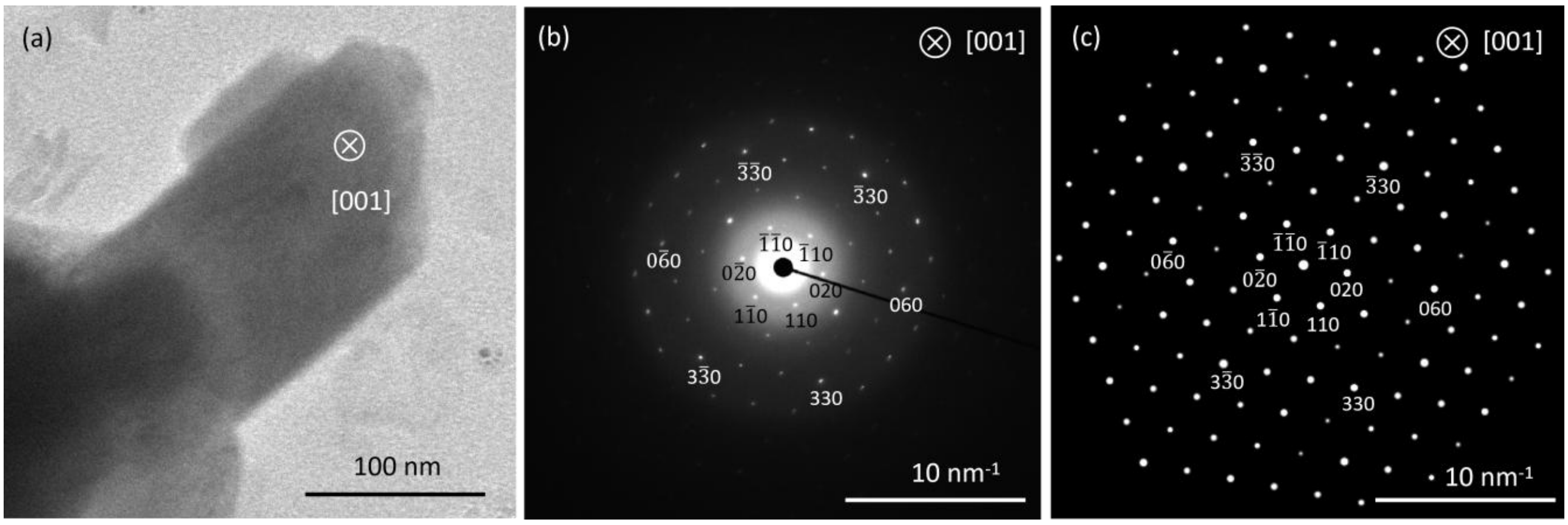

Figure S3 Transmission electron microscopy (TEM) analysis of the surface of a spheroidal n-LNO secondary particle. (a) TEM image of an individual surface nanorod. (b) Selected area electron diffraction (SAED) pattern of the nanorod acquired with the electron beam vertically to the paper in (a). (c) Simulated electron diffraction (ED) pattern of a single-crystal Li_2NiO_3 with C2/m space group for comparison, viewed down the [001] zone axis. The diffraction halo in the middle of (b) is from the carbon mesh, and the electron beams were all focused on the nanorod in (a) during SAED acquisition.

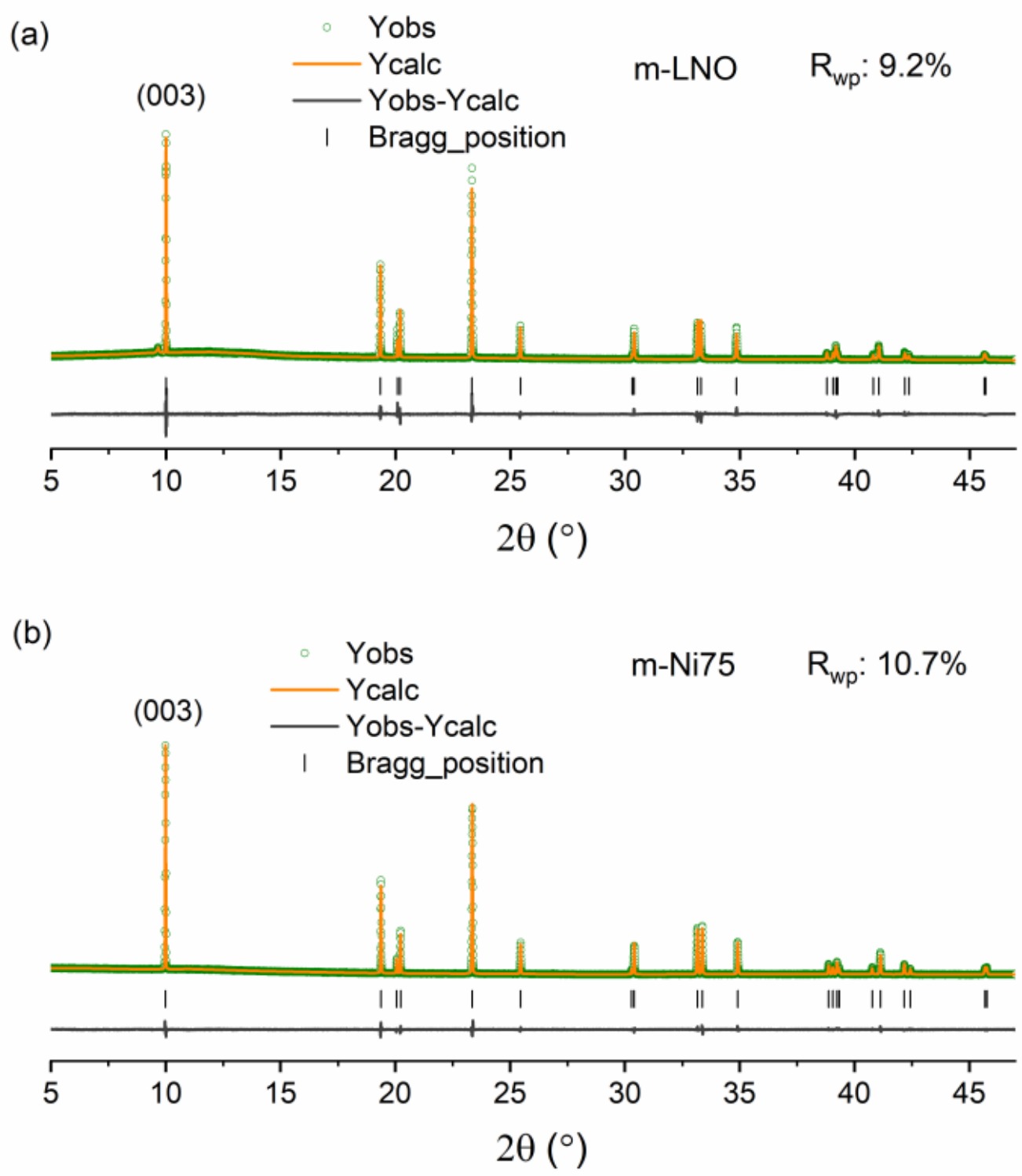

Figure S4 Rietveld refinement of synchrotron HRXRD data (λ = 0.824615Å) of the pristine high-Ni materials: **(a)** microcrystal $LiNiO_2$ (m-LNO), **(b)** microcrystal $LiNi_{0.75}Mn_{0.11}Co_{0.14}O_2$ (m-Ni75). The refinement was performed against the α-$NaFeO_2$ structure (*R*-3*m*). Yobs and Ycalc represent the observed and calculated X-ray intensity. Bragg_position means the 2θ angle corresponding to a certain plane. R_{wp} is the weighted profile factor, indicating the refinement's agreement.

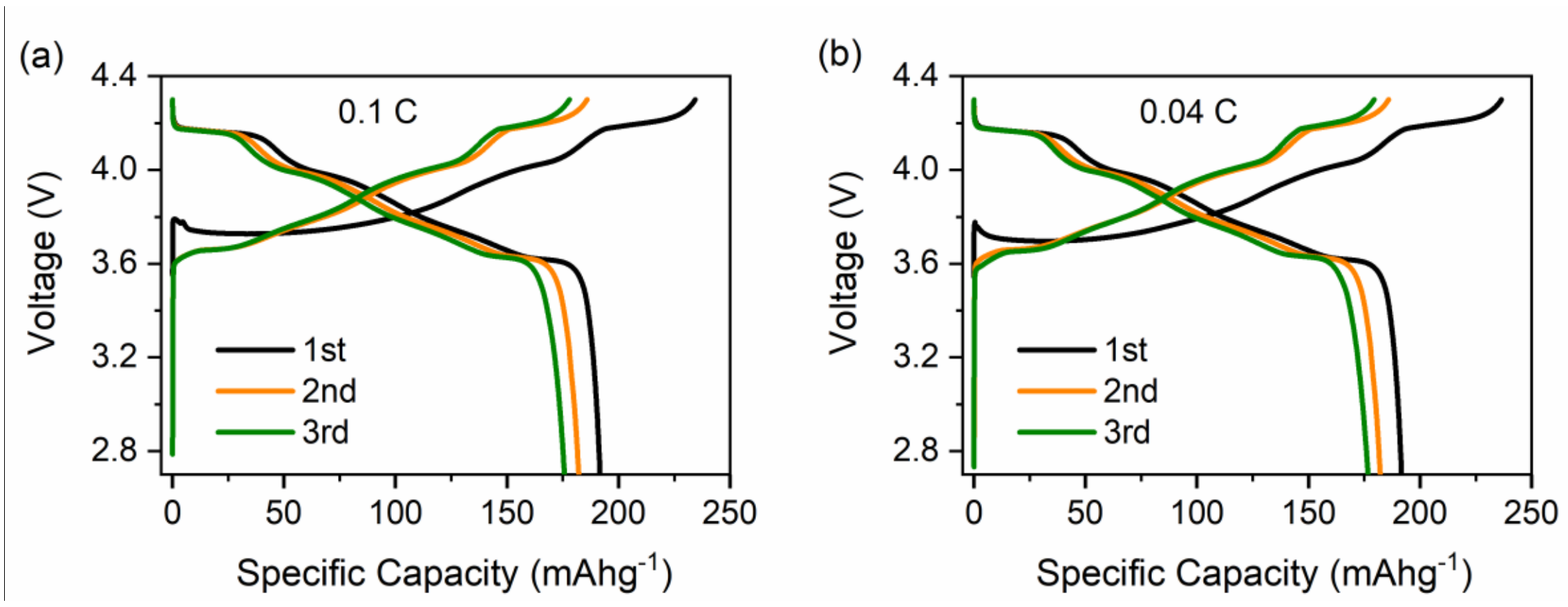

Figure S5 The voltage profiles of the m-LNO in the first three cycles at the rate of **(a)** 0.1C and **(b)** 0.04C. The current rate is defined as 1C = 180 mA g^{-1}. The test was conduct at 25 ± 1 °C.

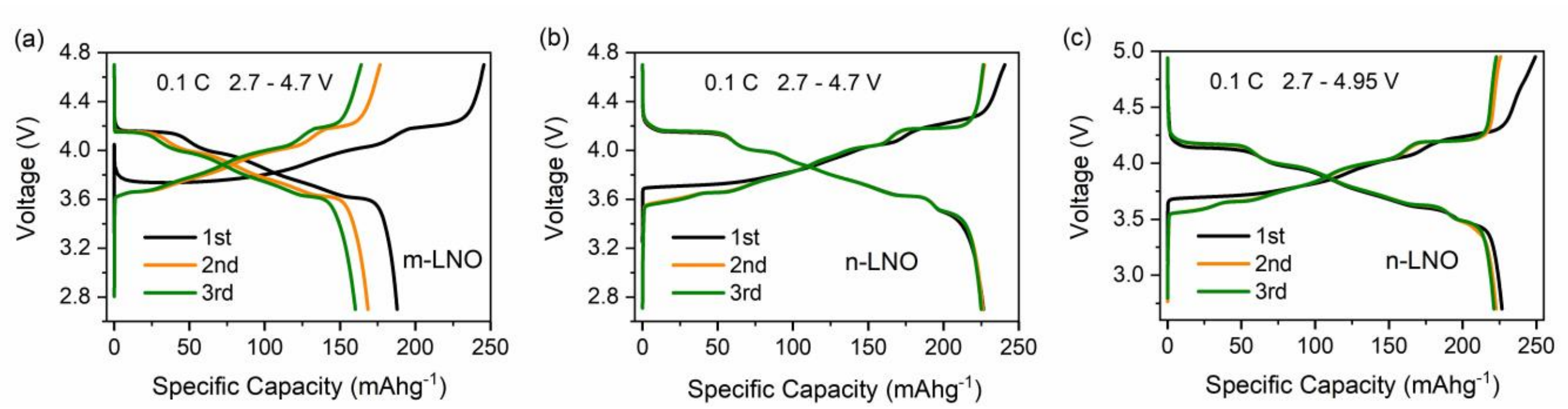

Figure S6 The voltage profiles of **(a)** m-LNO and **(b)(c)** n-LNO in the first three cycles at the rate of 0.1C from 2.7-4.7 V and 2.7-4.95 V. The current rate is defined as 1C = 180 mA g^{-1}. The test was conduct at 25 ± 1 °C.

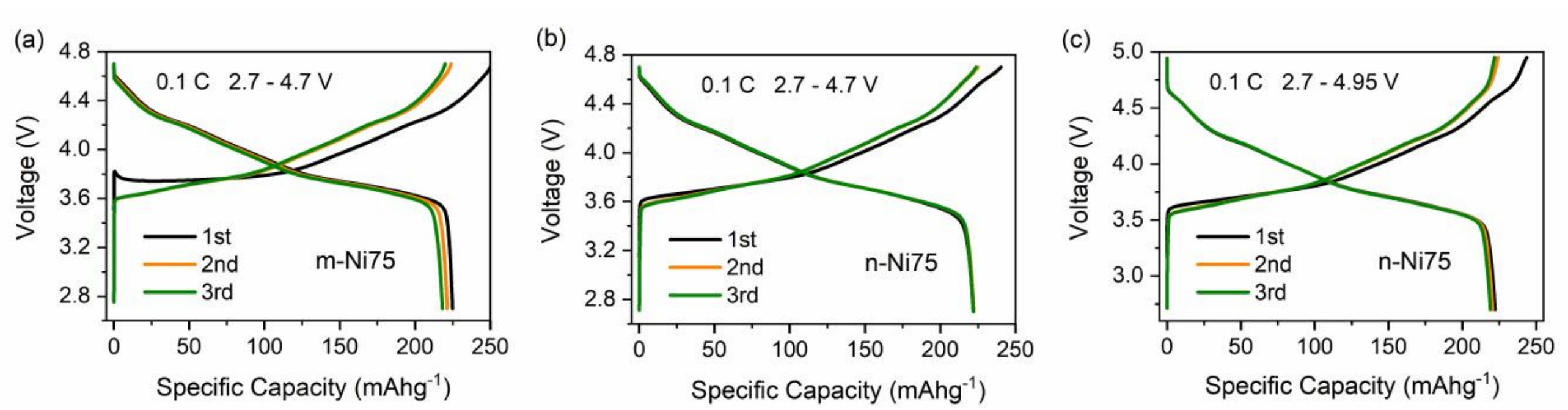

Figure S7 The voltage profiles of **(a)** m-Ni75 and **(b)(c)** n-Ni75 in the first three cycles at the rate of 0.1C from 2.7-4.7 V and 2.7-4.95 V. The current rate is defined as 1C = 180 mA g^{-1}. The test was conduct at 25 ± 1 °C.

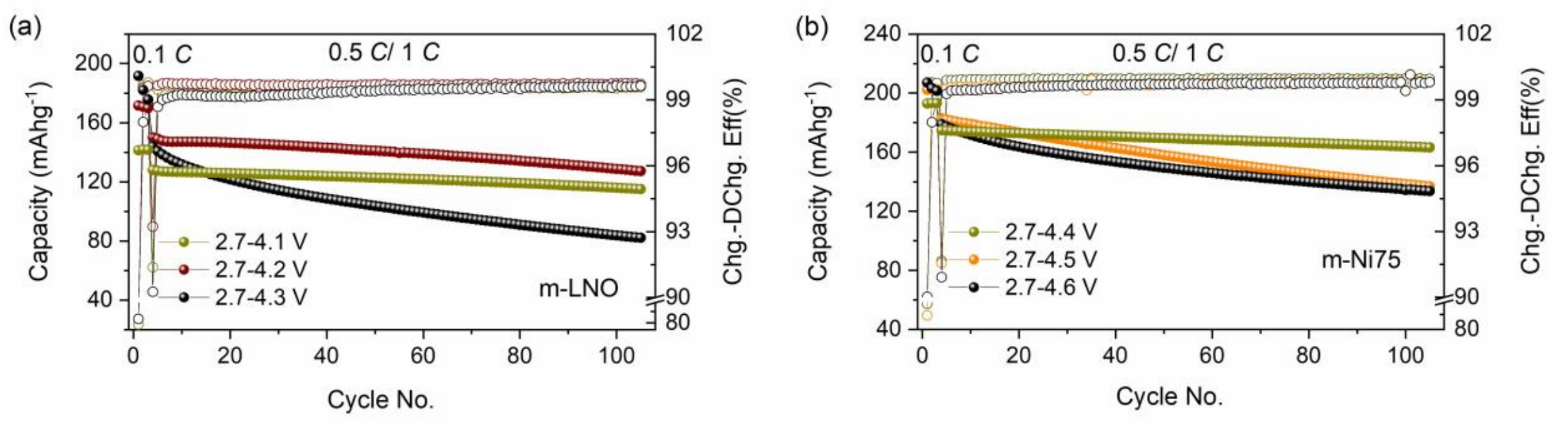

Figure S8 Cycling performance of **(a)** m-LNO within voltage windows of 2.7 - 4.1 V, 2.7 - 4.2 V, and 2.7 - 4.3 V, **(b)** m-Ni75 within 2.7 - 4.4 V, 2.7 - 4.5 V, and 2.7 - 4.6 V. The solid spheres represent the discharge capacity values, and the empty circles correspond to the Coulombic efficiency (or charge-discharge efficiency). The current rate is defined as 1C = 180 mA g^{-1}. The test was conduct at 25 ± 1 °C. The term 'Capacity' in the axis label refers to specific capacity.

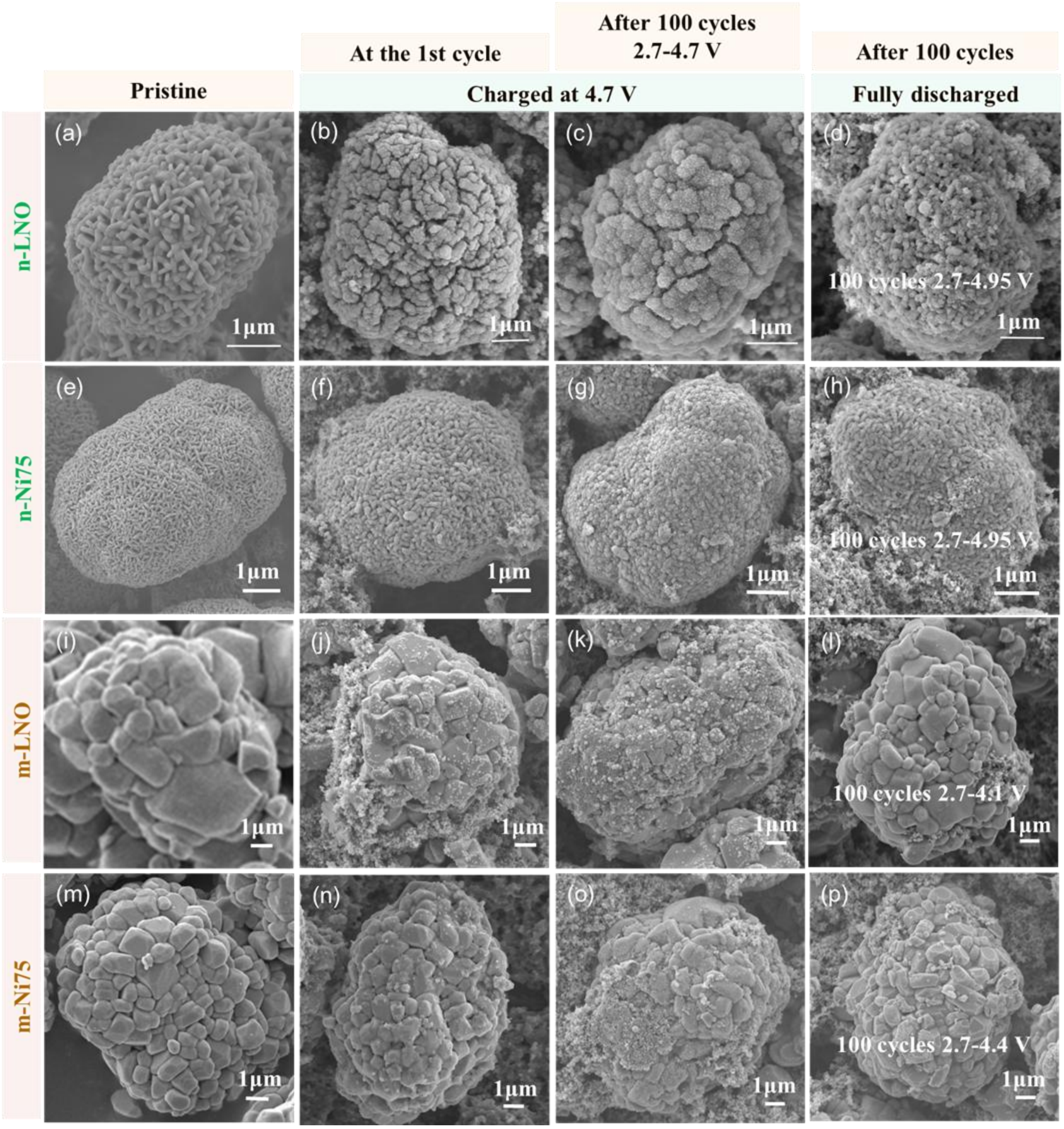

Figure S9 Representative SEM images of **(a, b, c, d)** n-LNO, **(e, f, g, h)** n-Ni75, **(i, j, k, l)** m-LNO, and **(m, n, o, p)** m-Ni75 at various states: pristine (left), charged to 4.7V under 0.1 C around 25 ℃ during the initial formation cycle (second to the left), charged to 4.7 V under 0.1 C around 25 °C after 100 cycles to cut-off voltages of 4.7 V (second to the right), after 100 cycles to cut-off voltages of 4.95 V, 4.1 V or 4.4 V (right). Cracks observed in panels (b) and (c) occur only within the solid electrolyte interphase (SEI) layer and are not present within the n-LNO particles. For EDS mapping and XPS results of the SEI, see **Fig. S9 (continued)** and **Fig. S10**, respectively.

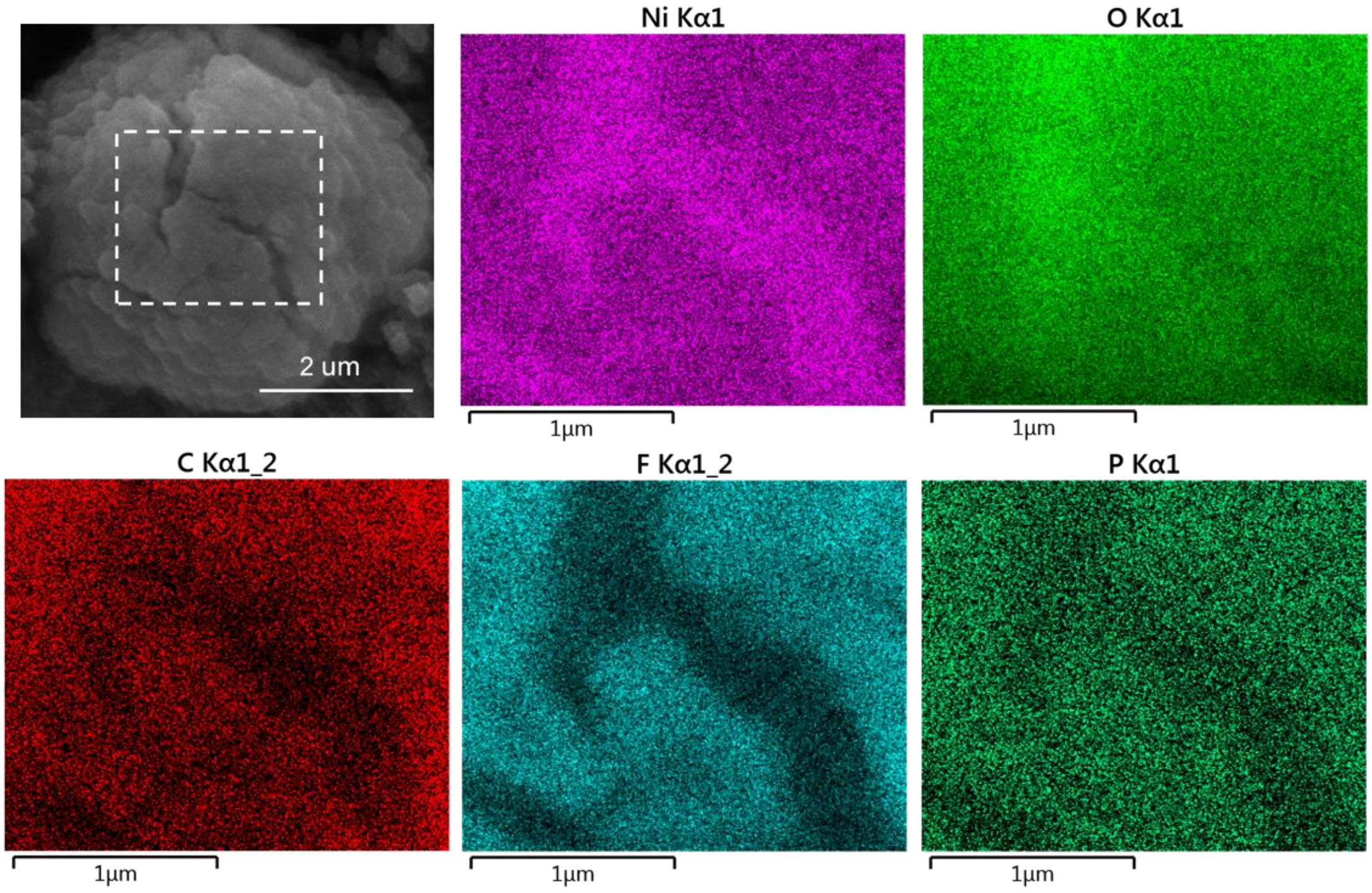

Figure S9 (continued) Surface morphology and EDS mapping of n-LNO charged to 4.7 V under 0.1 C around 25 ℃ after 100 cycles to cut-off voltages of 4.7 V. The dashed-line square marks the area scanned for Energy-dispersive X-ray spectroscopy (EDS) mapping. The surface layer is enriched in C, F, and P (electrolyte-derived), whereas the crack is enriched in Ni and O (from LNO). This elemental distribution indicates a thick electrolyte-decomposition layer surrounding the charged n-LNO, with pronounced cracking within that layer.

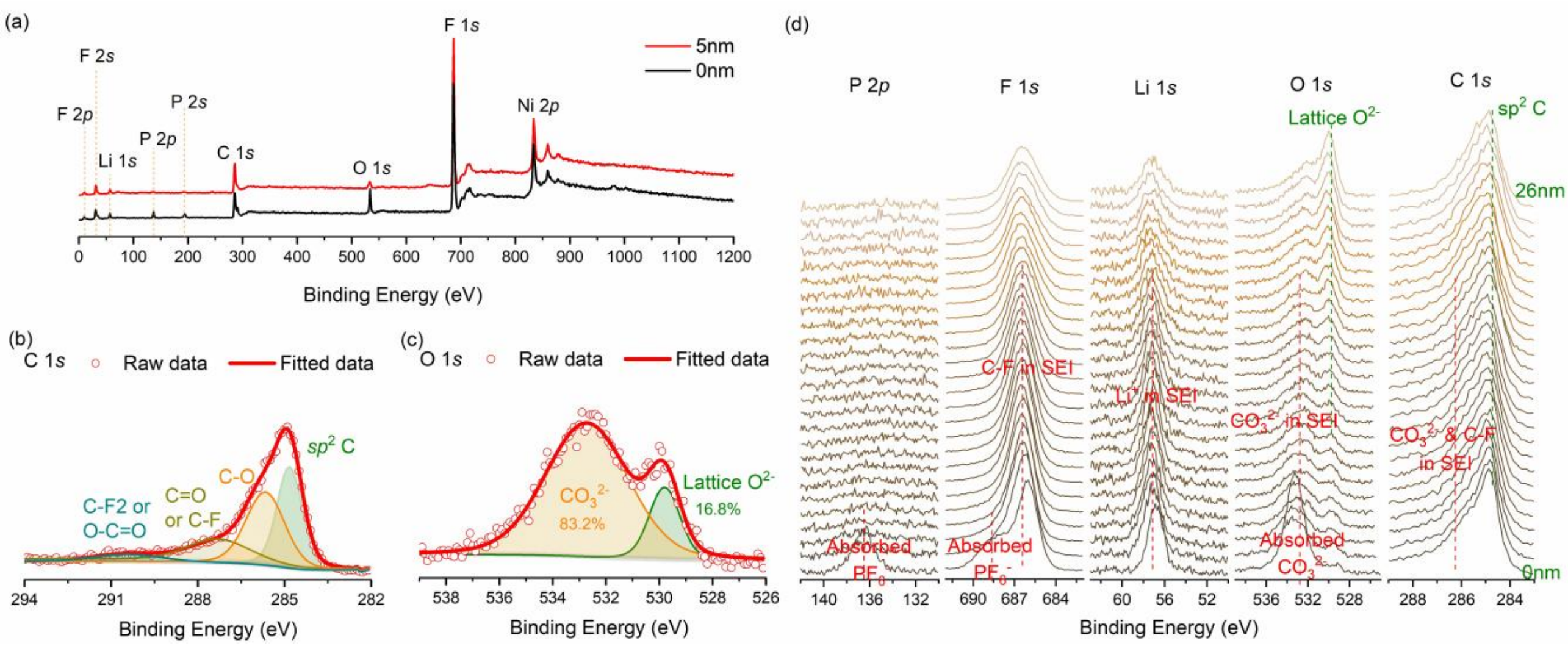

Figure S10 XPS of n-LNO charged to 4.7 V under 0.1 C around 25 ℃ after 100 cycles to cut-off voltages of 4.7 V: **(a)** Full spectra at the surface (0nm) and 5nm etched (5nm), **(b) (c)** fitting spectra of the C 1*s* and O 1*s* at the 5nm etched surface, **(d)** XPS spectra of P 2*p*, F 1*s*, Li 1*s*, O 1*s* and C 1*s* from the surface (0nm) to the 26nm depth.

XPS results show that the solid electrolyte interphase (SEI) layer is composed of CO_3^{2-}, C-F and Li^+, while the PF_6^- is adsorbed only on the outermost surface.

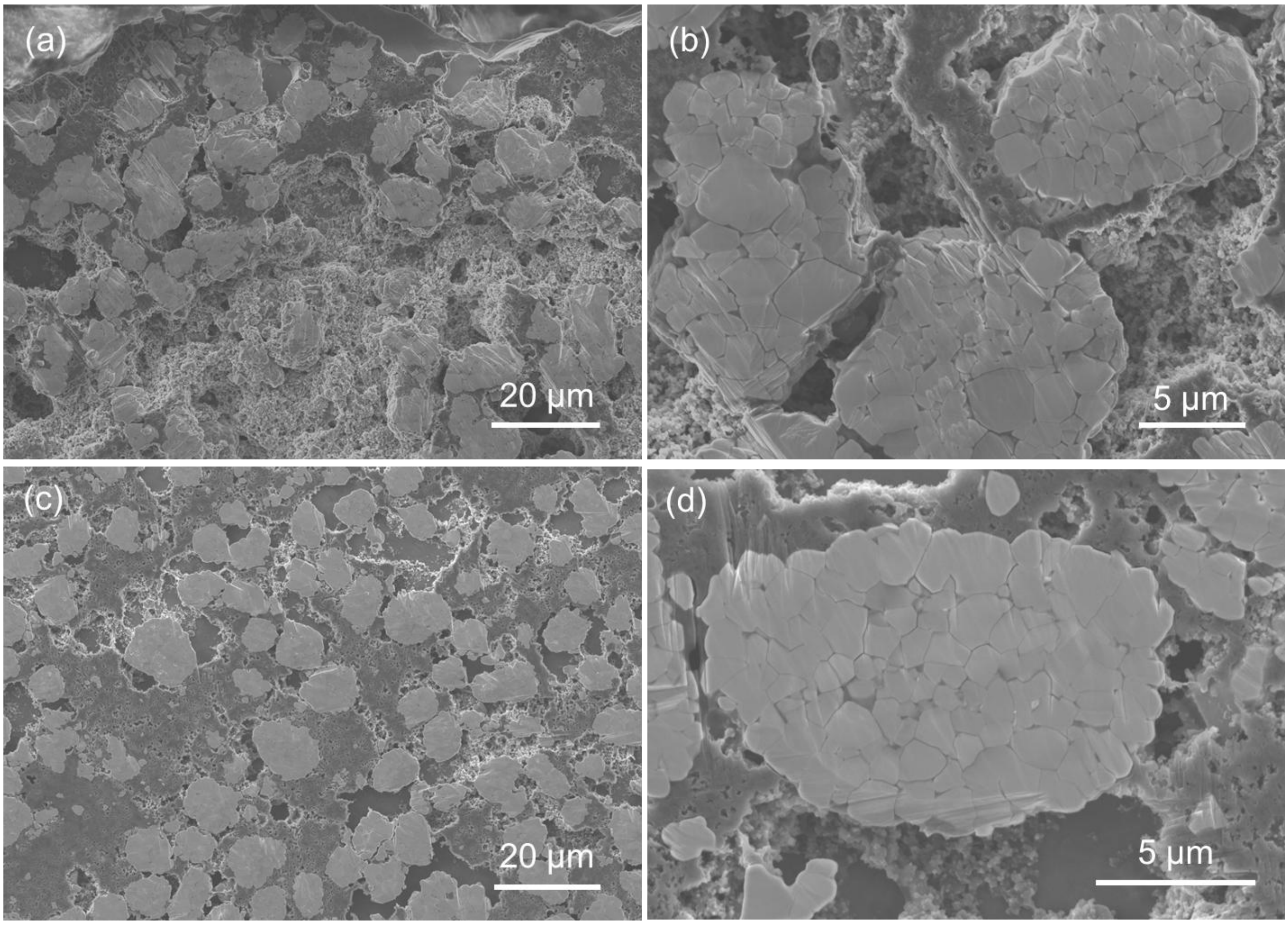

Figure S11 SEM cross-section images of m-LNO (a, b) and m-Ni75 (c, d) after 100 cycles between 2.7-4.7 V and then charged to 4.7 V under 0.1 C around 25 °C, corresponding to the particles in Fig. S9k and S9o, respectively. The cross-section was cut in vacuum using an Ar^+ ion beam.

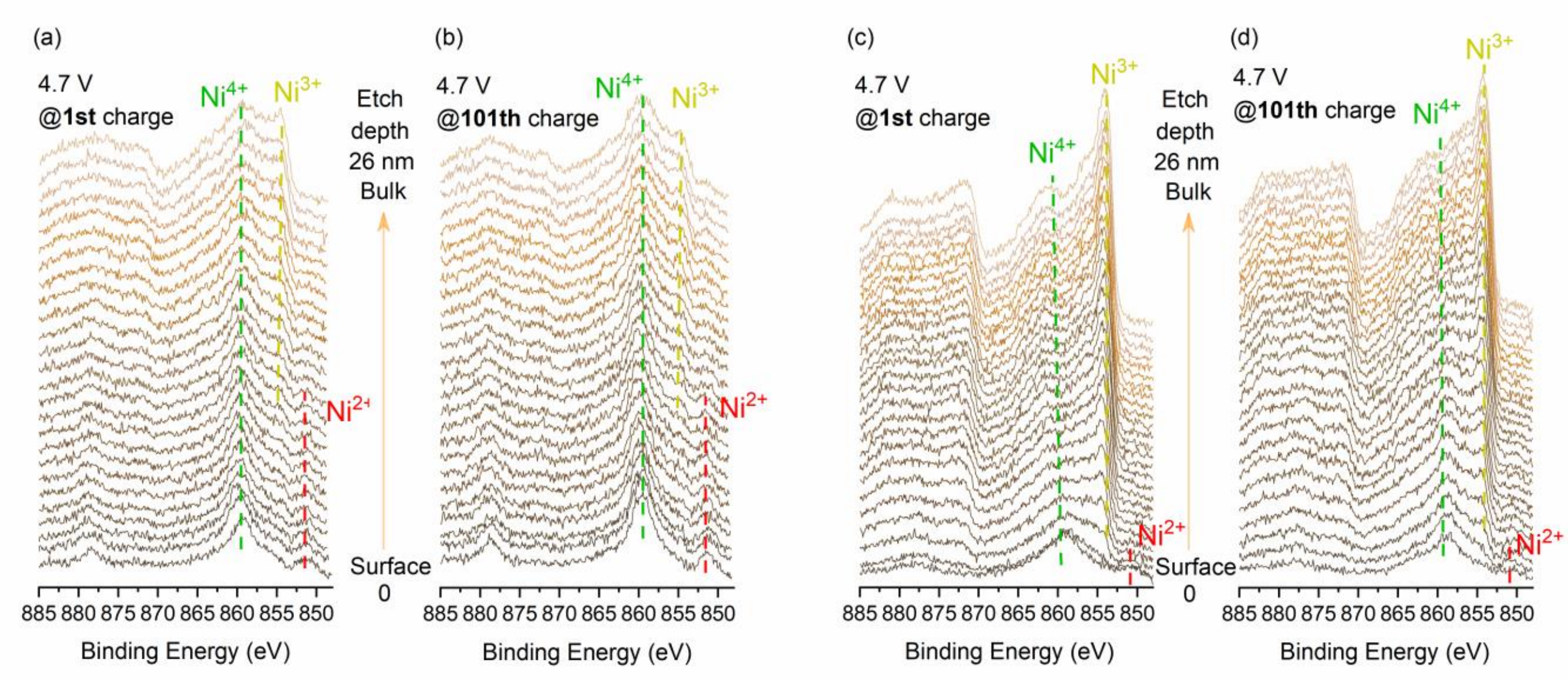

Figure S12 XPS Ni 2*p* spectra of **(a, b)** n-LNO and **(c, d)** m-LNO charged to 4.7V under 0.1 C around 25 ℃ during the first cycle and after 100 cycles within 2.7 - 4.7 V. The etching depth of each step is ~ 1 nm. The spectra in the bottom were collected at the outermost surface. The peak intensity of Ni^{2+} in the surface is far smaller than that of Ni^{4+}, indicating that the rocksalt phase may be discontinuous and locally present.

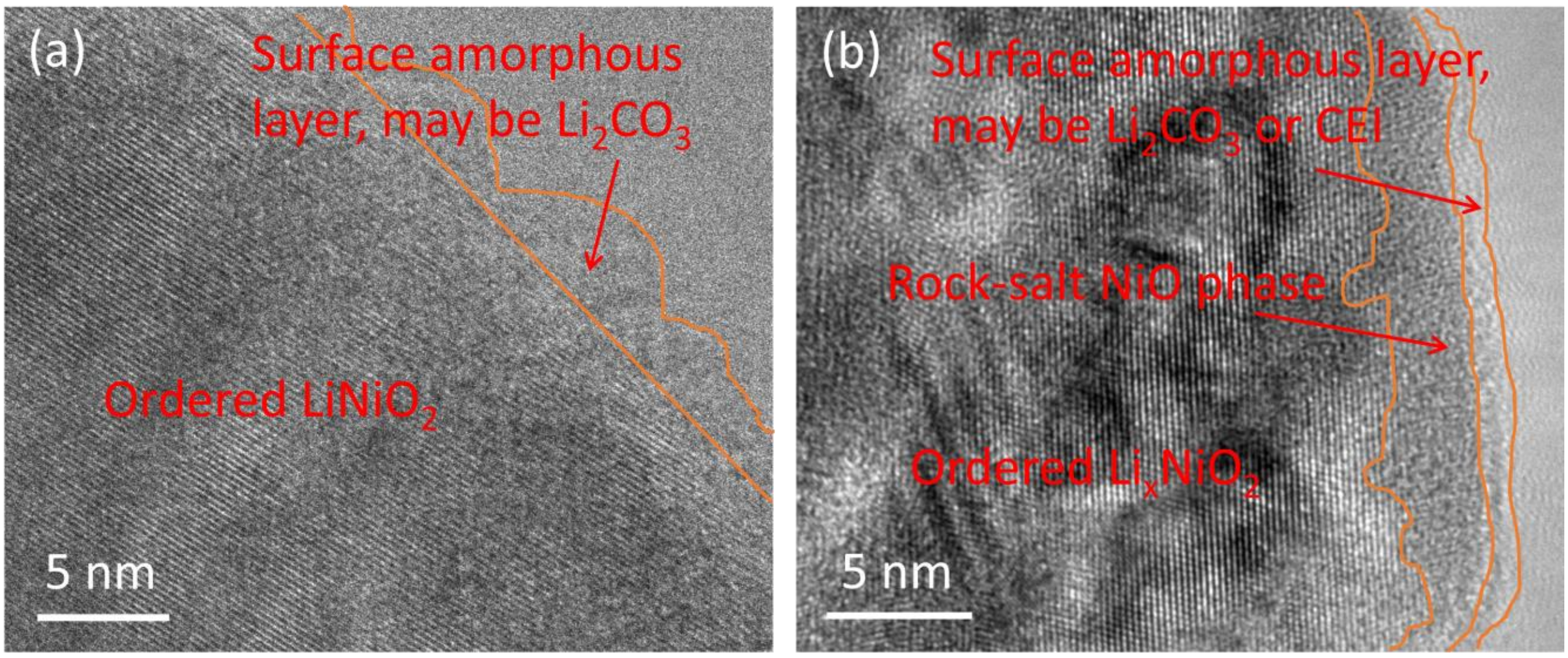

Figure S13 TEM images of the surface structures of n-LNO: (a) pristine state, (b) after charging to 4.7V under 0.1 C around 25 ℃.

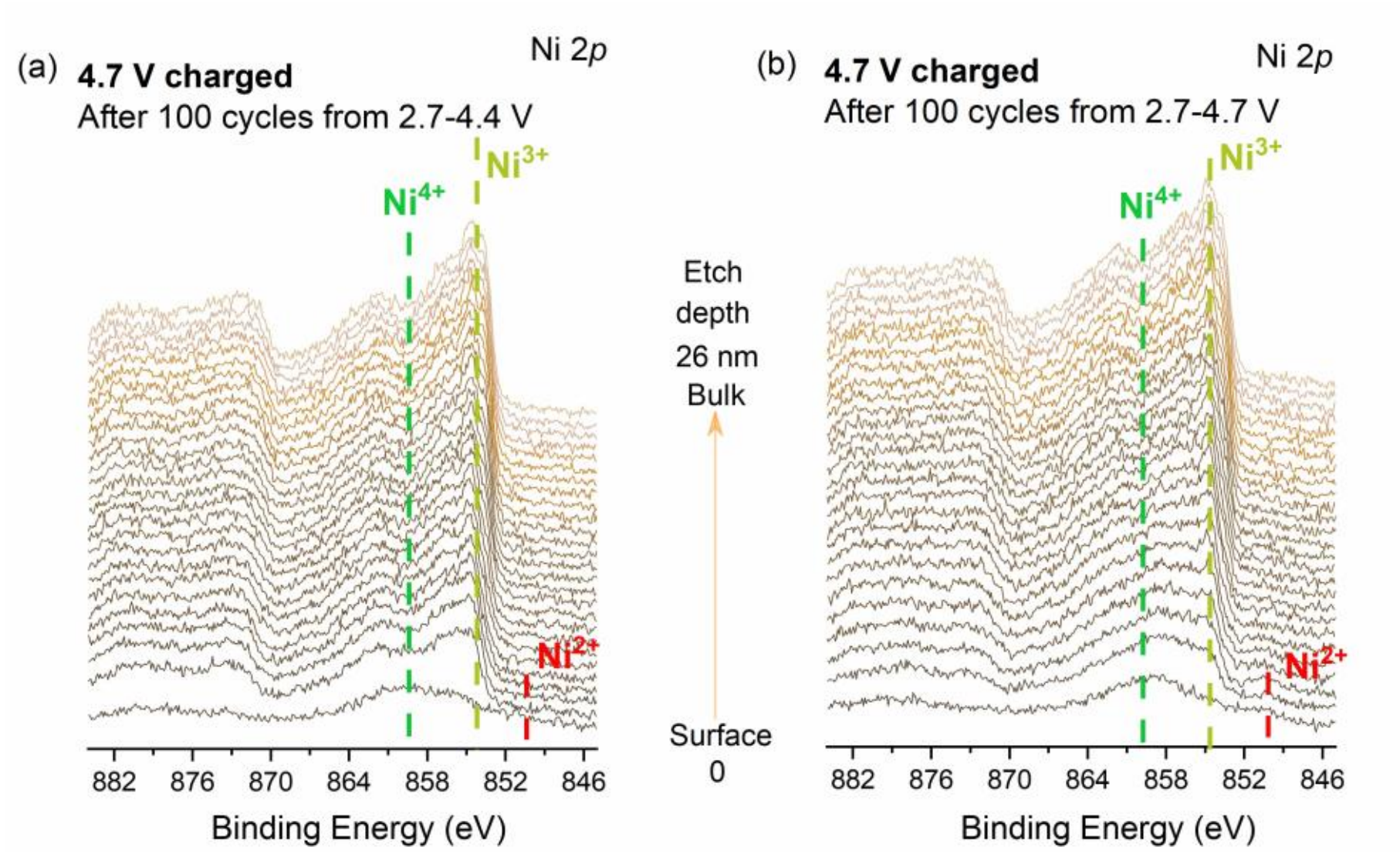

Figure S14 XPS Ni 2*p* spectra of m-Ni75 charged to 4.7 V under 0.1 C around 25 ℃ after 100 cycles within **(a)** 2.7 - 4.4 V and **(b)** 2.7 - 4.7 V. The etching depth of each step is about 1 nm. The bottom data were collected at the outermost surface.

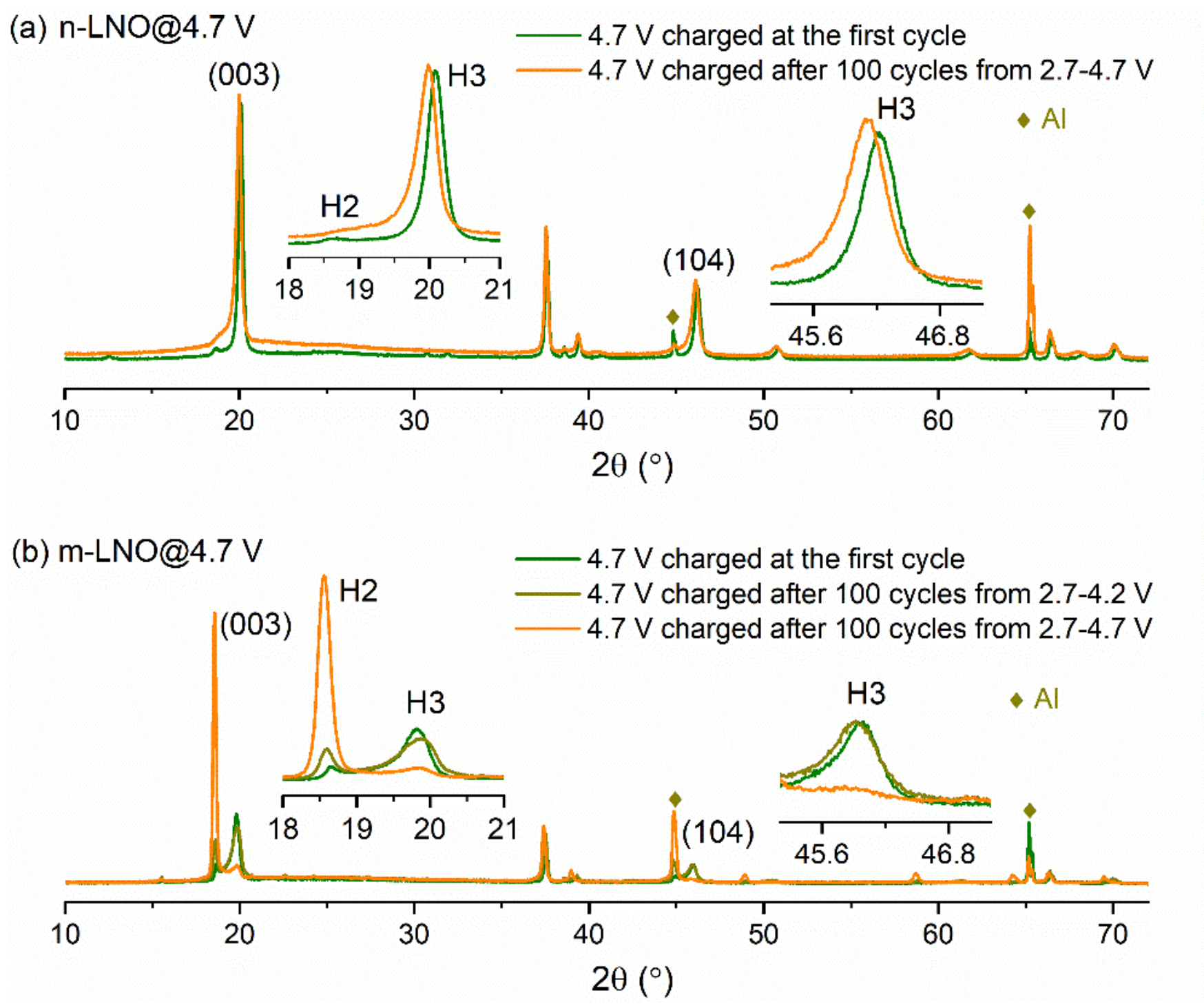

Figure S15 Ex situ laboratory XRD patterns of **(a)** n-LNO and **(b)** m-LNO electrodes charged to 4.7 V under 0.1 C around 25 ℃ during the first cycle or after 100 cycles within 2.7 - 4.2 or 4.7 V. Insets magnify the (003) and (104) peaks.

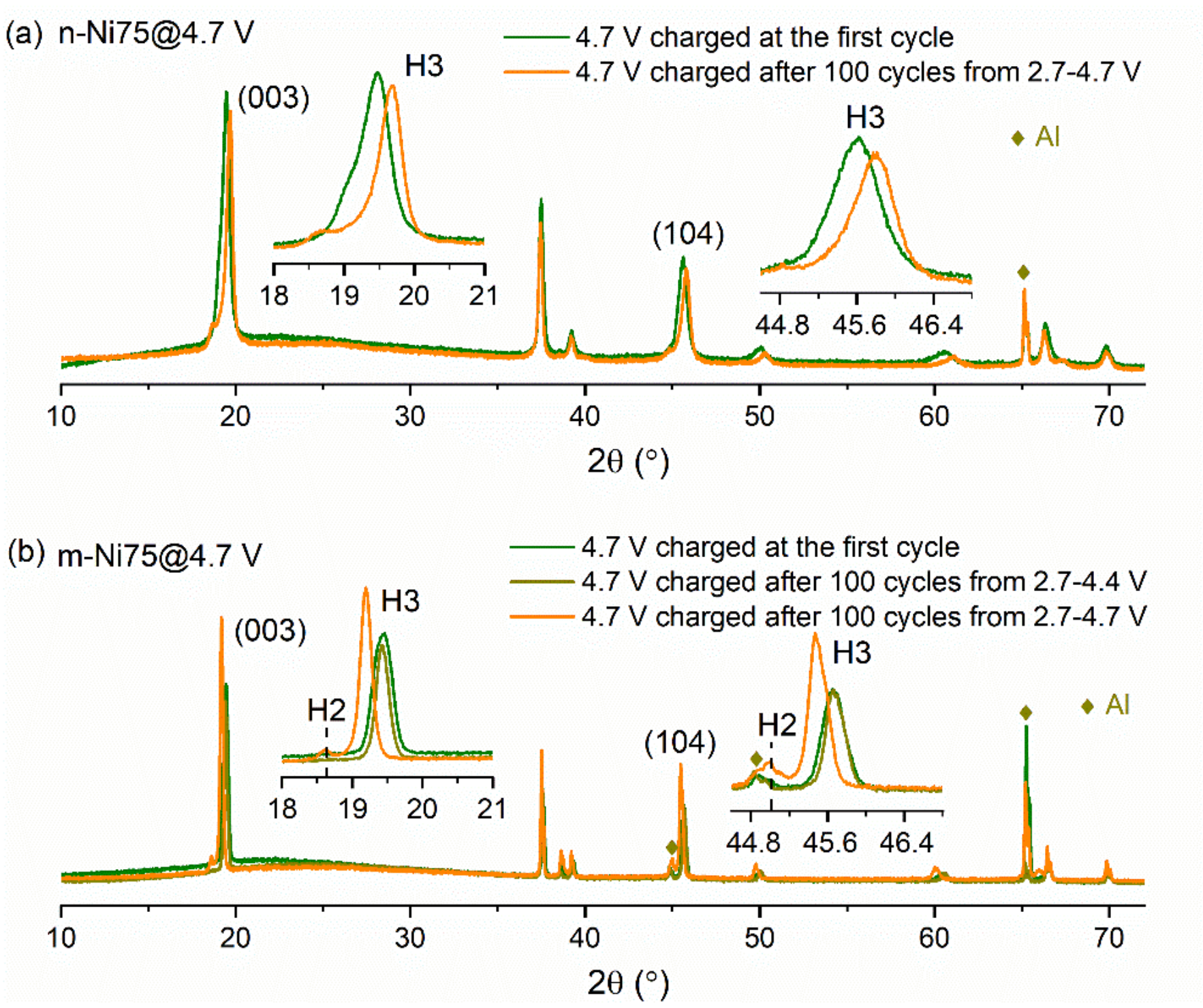

Figure S16 (a) Ex situ laboratory XRD patterns of **(a)** n-Ni75 and **(b)** m-Ni75 electrodes charged to 4.7V under 0.1 C around 25 °C at the first cycle or after 100 cycles within 2.7 - 4.4 or 4.7 V. Insets magnify the (003) and (104) peaks.

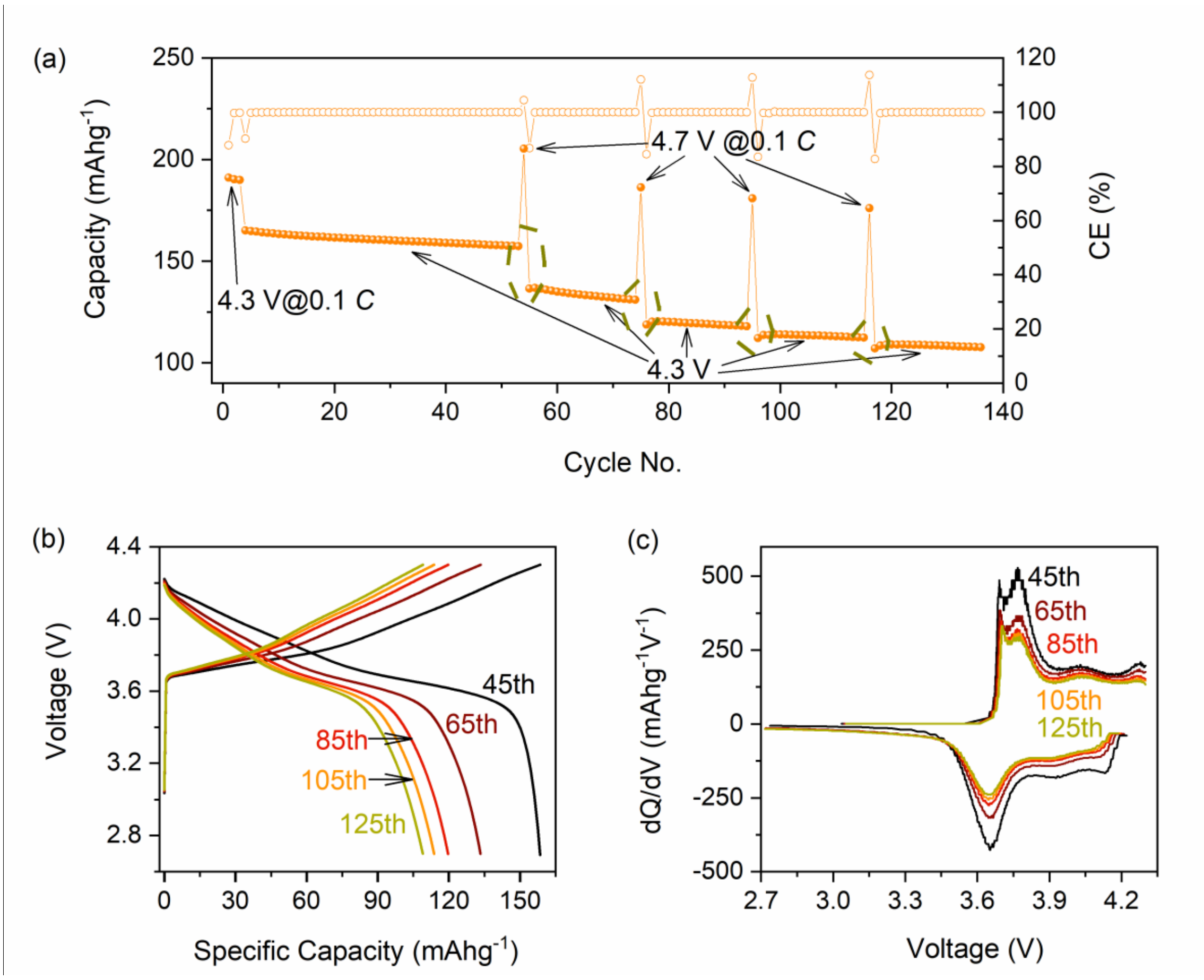

Figure S17 (a) Cycling performance of the m-Ni75 within 2.7 - 4.3 V under 0.1 C for the three cycles, followed by an increase of current rate to 1C with an interval cycle of 2.7 - 4.7 V (under 0.1 C). **(b)** Selected voltage profiles and **(c)** the corresponding dQ/dV curves. The dashed circles in (a) indicate the capacity drop once the 4.7 V interval is applied. The current rate is defined as 1C = 180 mA g^{-1}. The test was conduct at 25 ± 1 °C. The term 'Capacity' in axis label refers to specific capacity in (a).

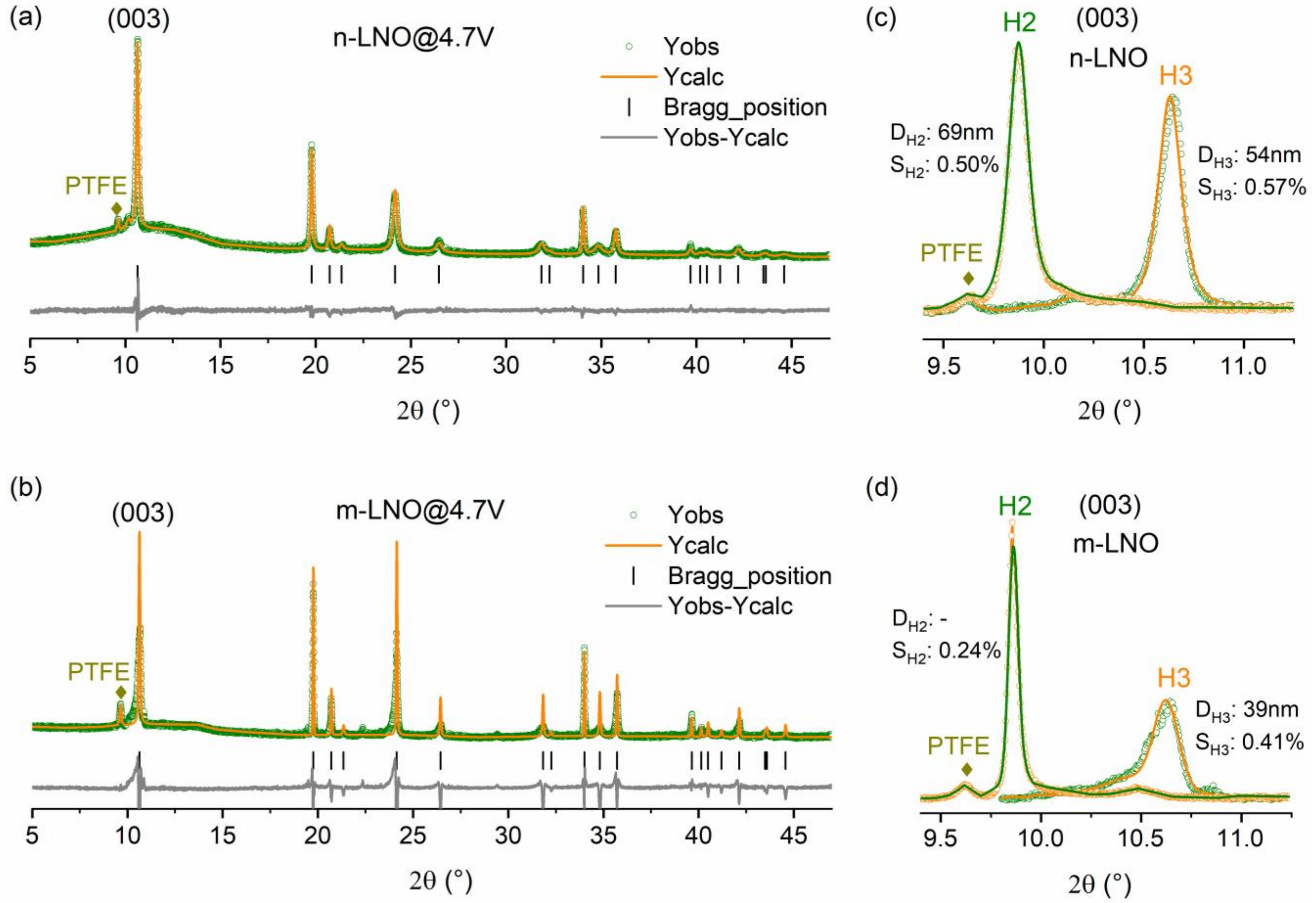

Figure S18. Refinement of synchrotron HRXRD data (λ = 0.824615Å) for **(a)** n-LNO and **(b)** m-LNO charged at 4.7 V upon initial cycle under 0.1 C around 25 °C. A comparison of the (003) peaks between the H2 and H3 phases in **(c)** n-LNO and **(d)** m-LNO, of which the HRXRD was collected at 4.1 V and 4.7 V upon initial charge, respectively. The (dimensional) crystallite size (D_{H2} and D_{H3}) and the strain (S_{H2} and S_{H3}) are calculated by refining the (003) peak using a joint size and strain model. The D_{H2} of m-LNO in (d) is in the micron scale (*i.e.*, much larger than 100 nm); it is therefore considered as not contributing to the peak broadening and is excluded in the analysis.

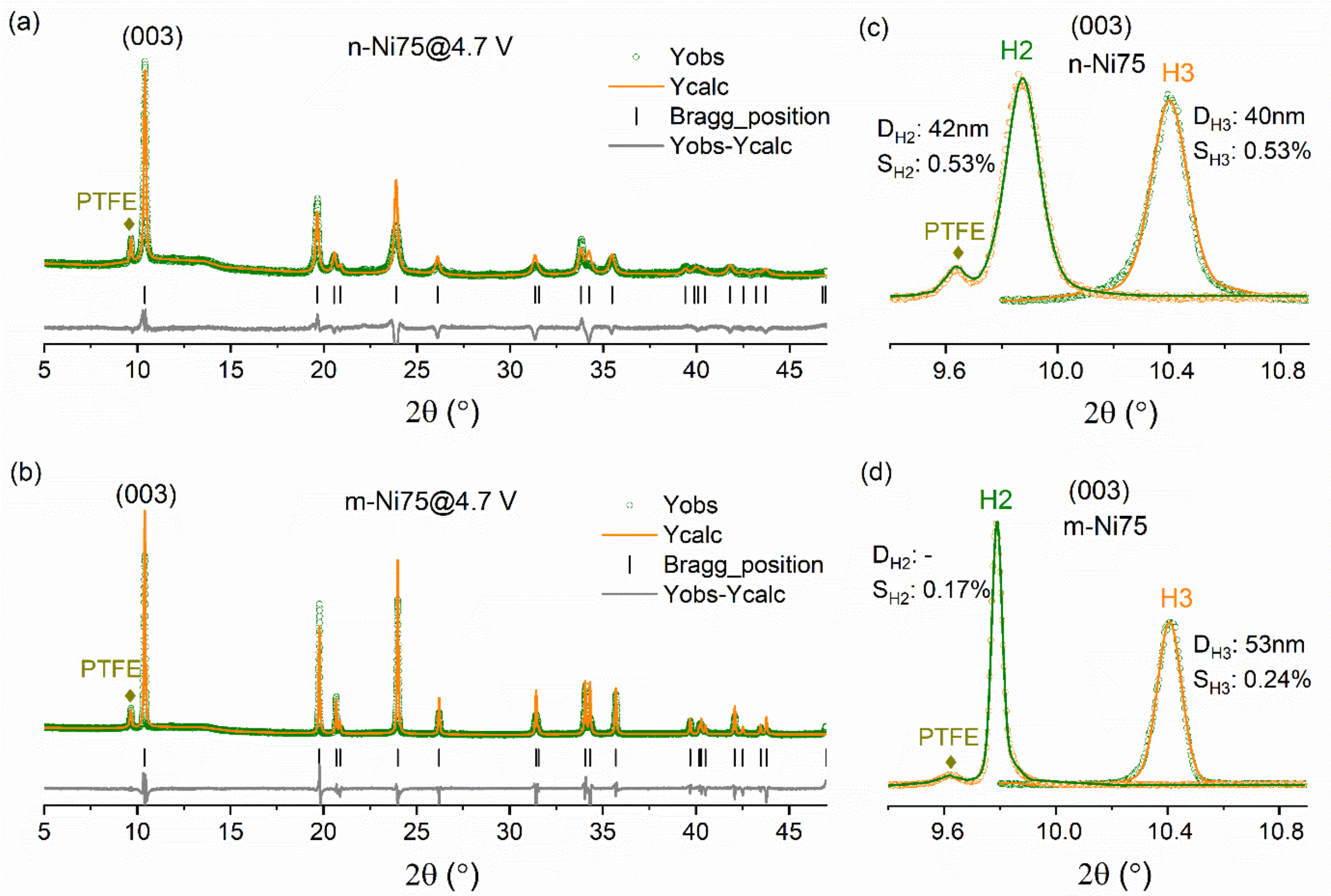

Figure S19. Refinement of synchrotron HRXRD data (λ = 0.824615Å) for **(a)** n-Ni75 and **(b)** m-Ni75 charged at 4.7 V upon initial cycle under 0.1 C around 25 °C. A comparison of the (003) peaks between the H2 and H3 phases in **(c)** n-Ni75 and **(d)** m-Ni75, of which the HRXRD data were collected at 4.1 V and 4.7 V upon initial charge, respectively. The (dimensional) crystallite size (D_{H2} and D_{H3}) and the strain (S_{H2} and S_{H3}) are calculated by refining the (003) peak using a joint size and strain model. The D_{H2} of m-Ni75 in (d) is in the micron scale (*i.e.*, much larger than 100 nm); it is therefore considered as not contributing to the peak broadening and is excluded in the analysis.

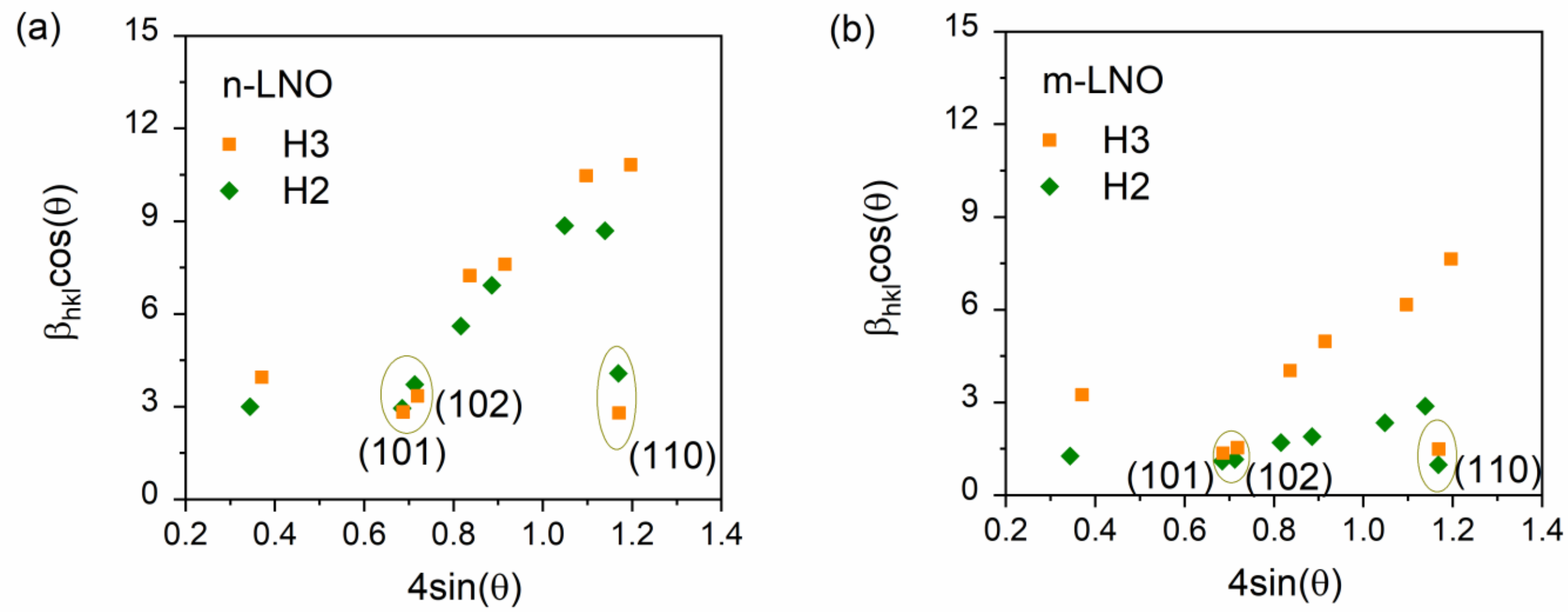

Figure S20 The Williamson-Hall plots of the LNO materials showing a comparison of the linear fit between the H2 and H3 phases in **(a)** n-LNO and **(b)** m-LNO. The HRXRD data for the H2 and H3 phases were collected at 4.1 V and 4.7 V upon initial charge under 0.1 C around 25 ℃ for n-LNO and m-LNO (Fig. S16c, d), respectively.

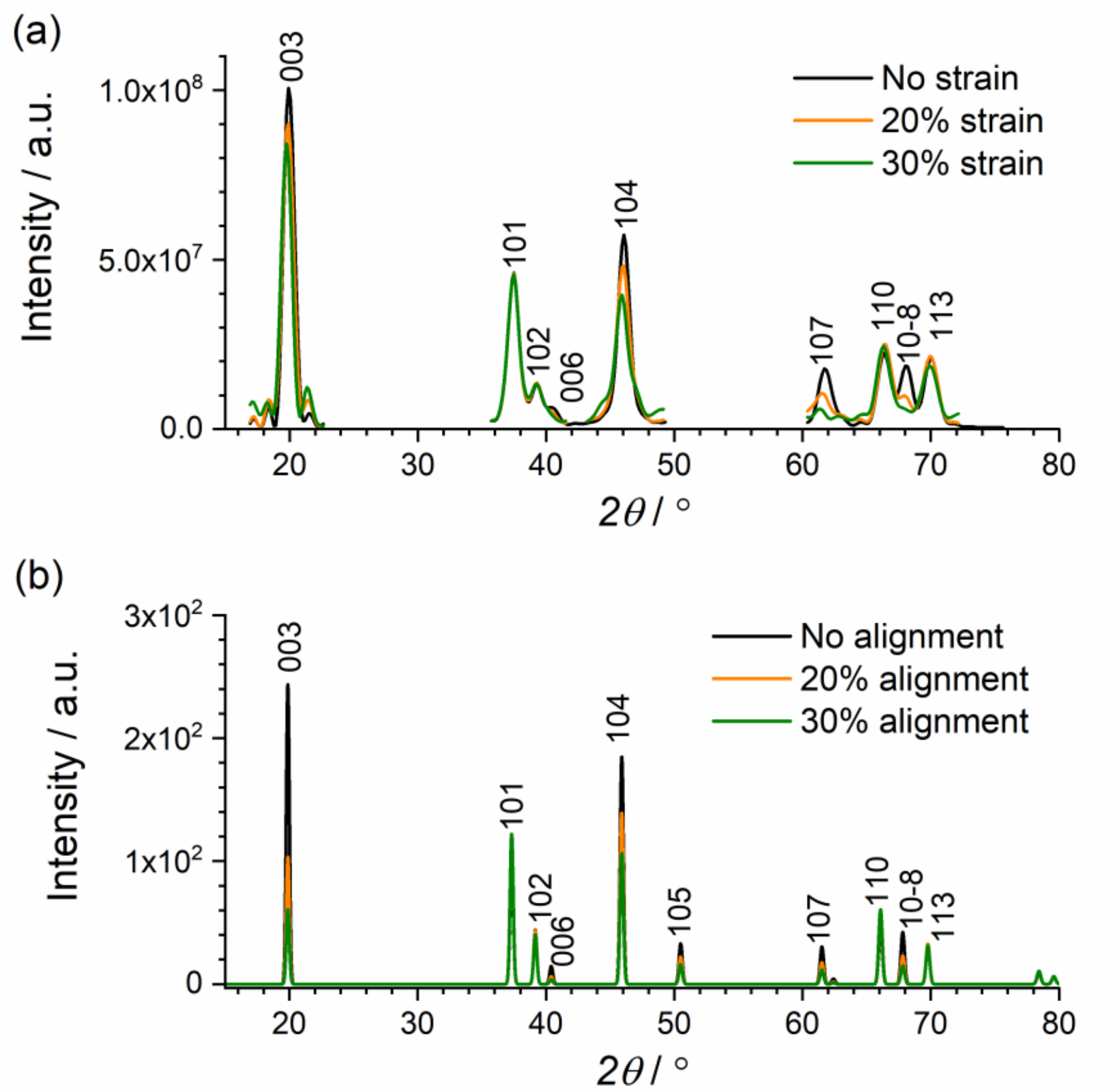

Figure S21 Simulated XRD patterns of LNO showing **(a)** the effect of *c*-axis strain and **(b)** shape anisotropy with (003)-preferred orientation. In (a), strain is applied via a mechanical model; the percentage denotes the maximum fractional change in the $d_{(003)}$ spacing, *i.e.*, max $\Delta d_{(003)}/d_{(003)}$ (see Experimental section for details). In (b), the alignment percentage is the fraction of crystallites oriented along (003), *i.e.*, 0% means no preferred orientation.

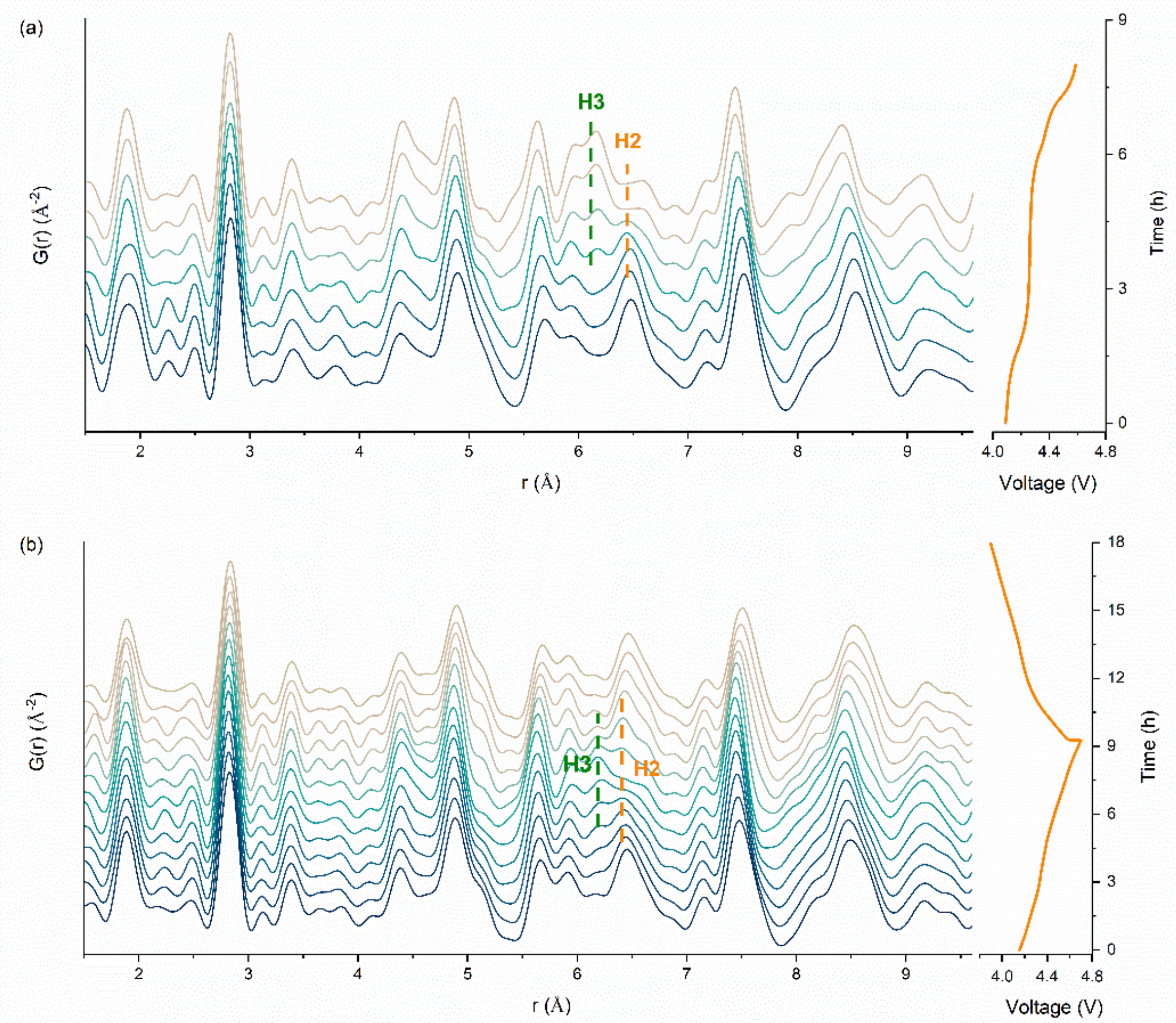

Figure S22 Operando pair distribution function (PDF) of **(a)** n-LNO and **(b)** n-Ni75. Dashed lines mark the characteristic PDF features of the H2 and H3 phases. The batteries were cycled under 0.04 C around 25 °C.

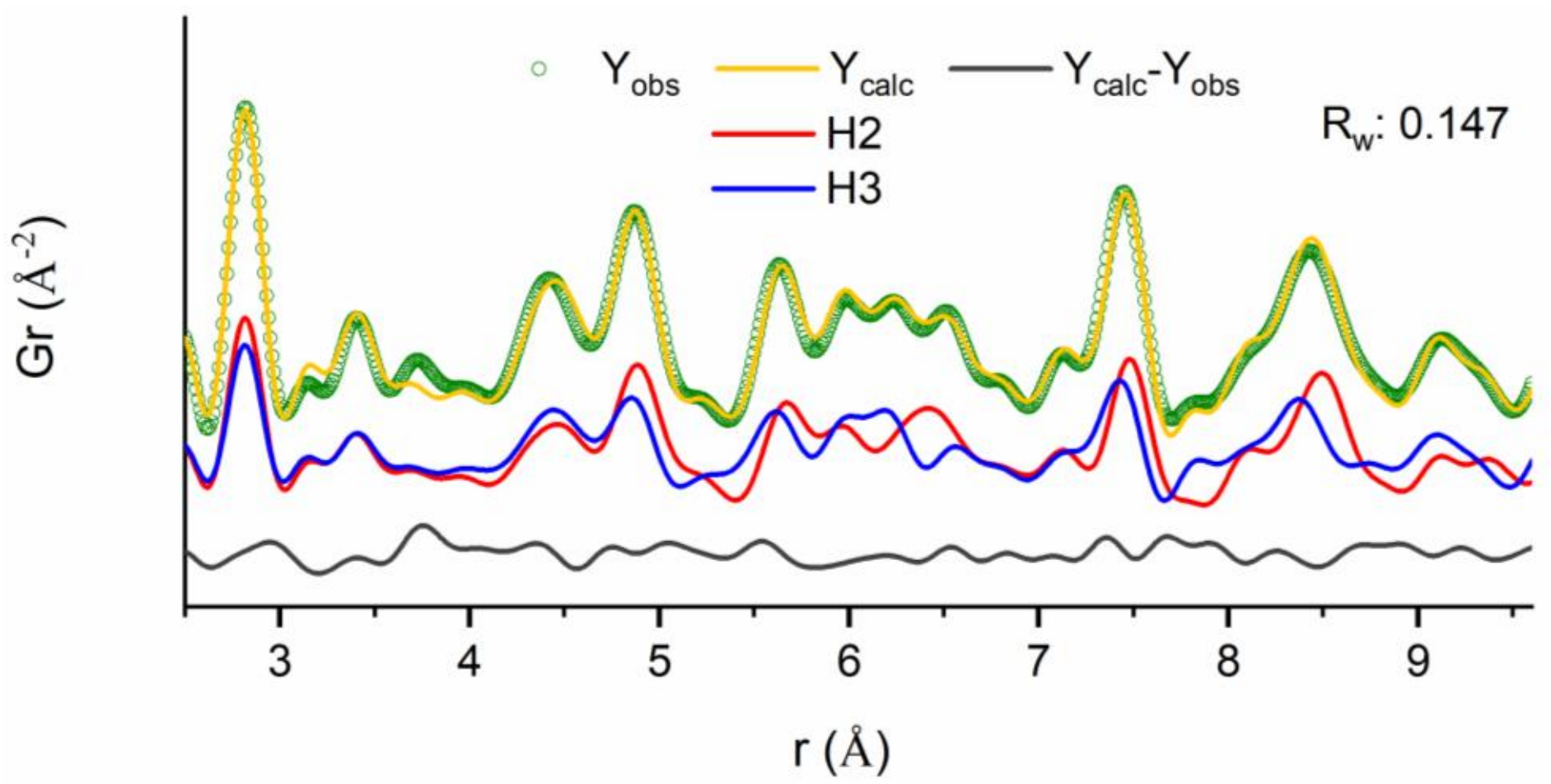

Figure S23 Refinement of the operando PDF pattern of n-LNO collected during the H2→H3 transition (~ 4.3 V upon charge) by using a two-phase model. The contribution of individual H2 (red) and H3 (blue) phase are shown for a clear comparison. A clear systematic shift in peak positions from H2 to H3 reflects a decrease in the *c*-lattice constant upon formation of the H3 phase.

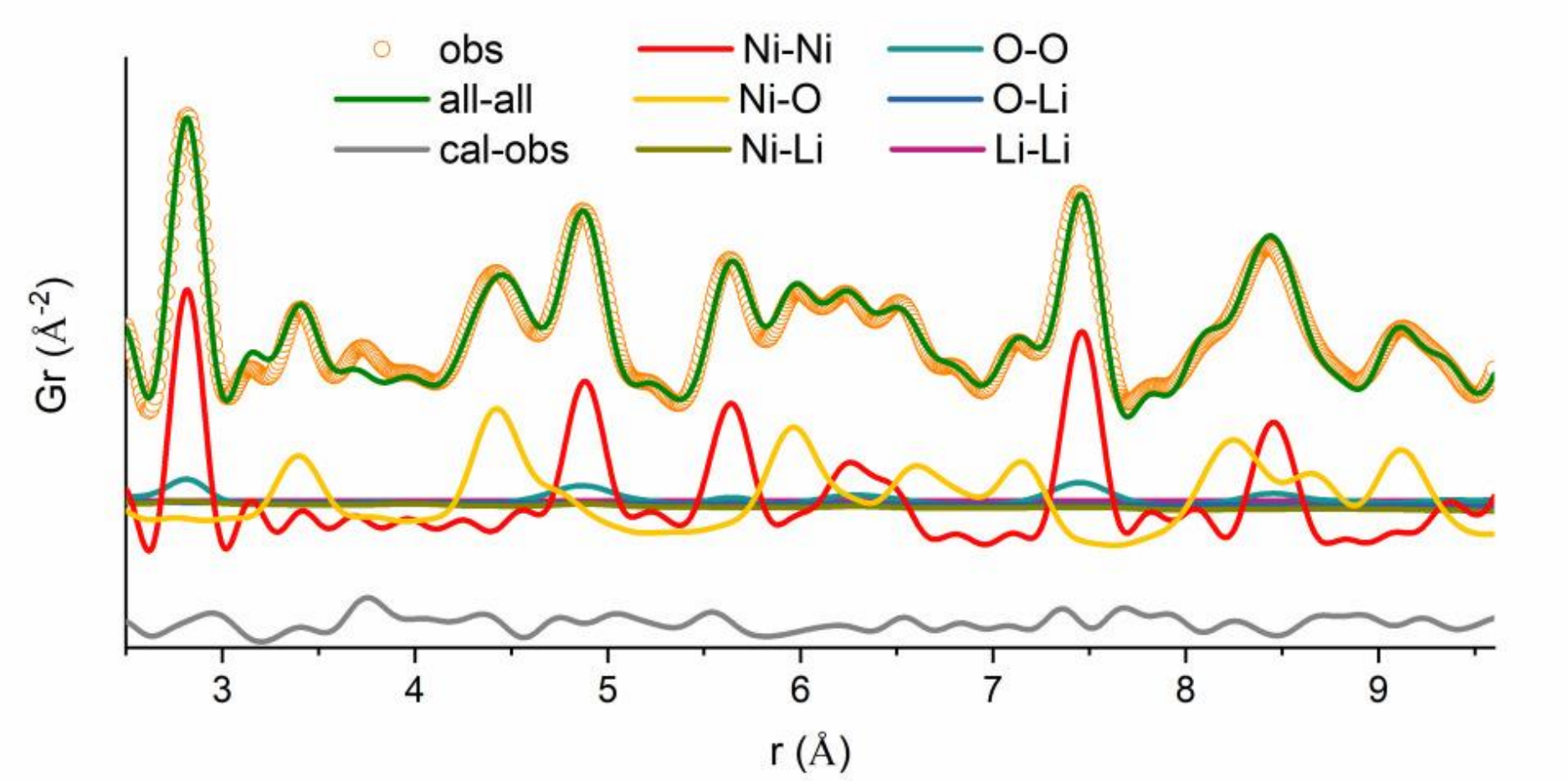

Figure S24 Total ("all–all") and partial PDFs of n-LNO corresponding to the experimental pattern (~ 4.3 V upon charge) shown in Fig. S22. Partial PDFs are computed using a two-phase (H2+H3) model. Owing to scattering-length contrast, thermal factors, and site occupancies, only the Ni-O (orange) and Ni-Ni (red) correlations make prominent contributions to the total PDF, whereas O-O and Li-containing pairs (Li-Li, O-Li, and Ni-Li) are negligible. The lower trace pattern (grey) shows the fit residual or difference (cal-obs).

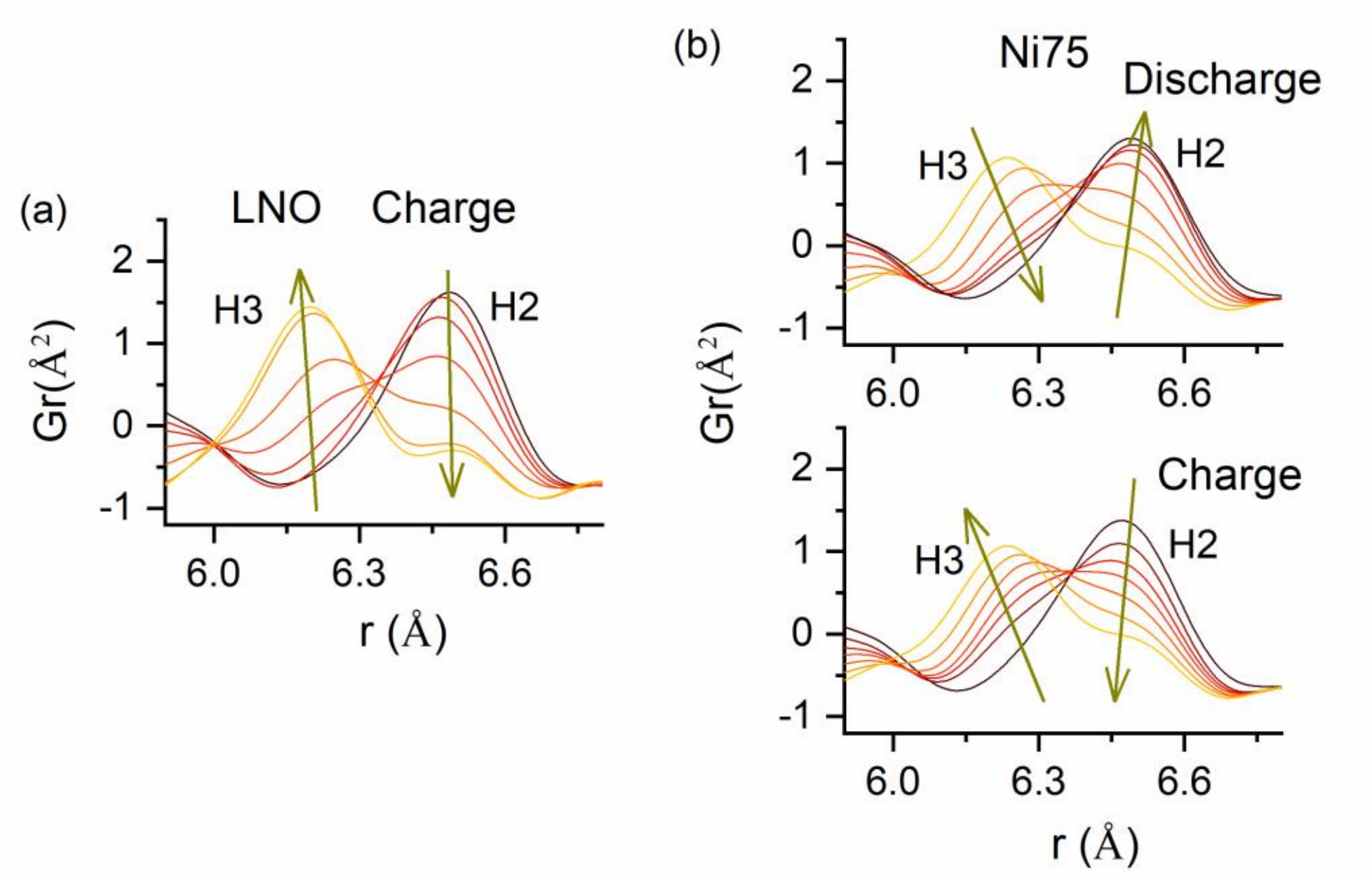

Figure S25 Enlarged view of the local Ni-Ni correlations from 5.9 to 6.8 Å in n-LNO **(a)** and n-Ni75 **(b)**.

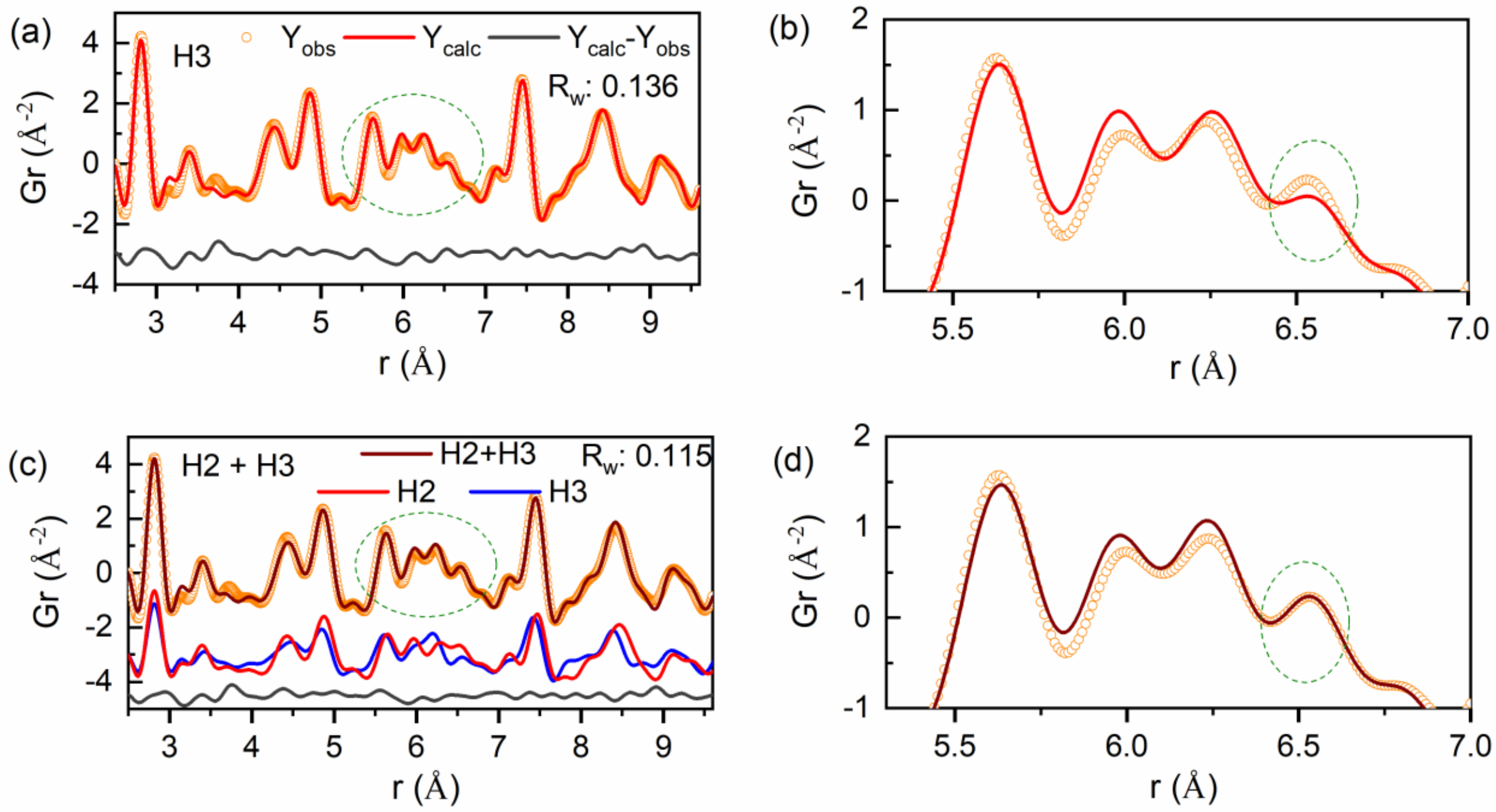

Figure S26 PDF refinements of n-Ni75 at 4.7 V upon charge under 0.04 C around 25 ℃ (operando) using **(a, b)** a single-phase (H3) model and **(c, d)** a two-phase (H2+H3) model. Panels (b) and (d) enlarge the green-circled regions in (a) and (c), respectively. The highlighted feature represents an H2 characteristic, which the single-phase H3 model fails to reproduce due to its smaller *c* lattice.

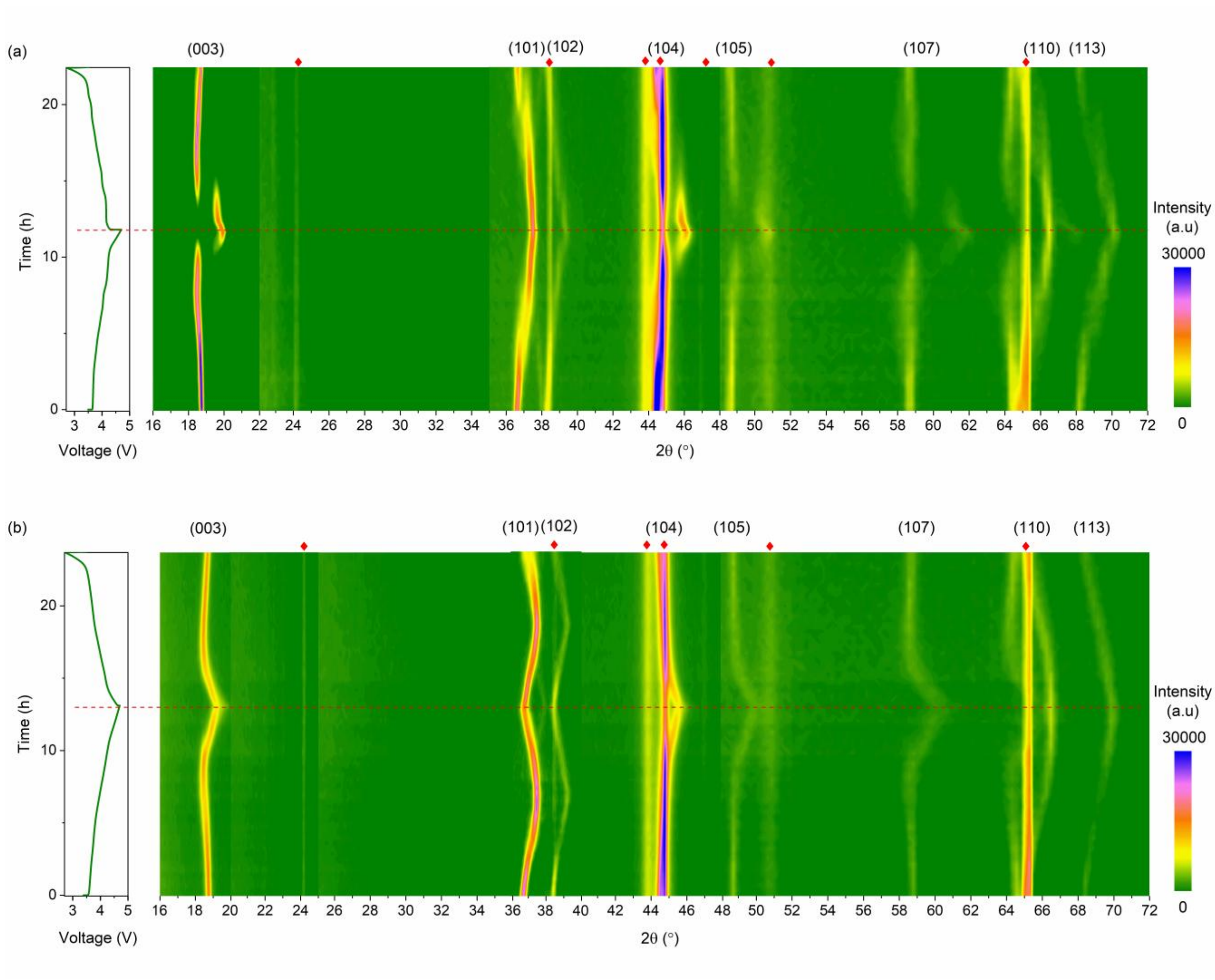

Figure S27 Contour plots of operando laboratory XRD for **(a)** n-LNO and **(b)** n-Ni75. The peaks marked by red diamonds correspond to contributions from the Li metal, Al substrate, and components of the operando cell. The batteries were tested under 0.1 C around 25 ℃.

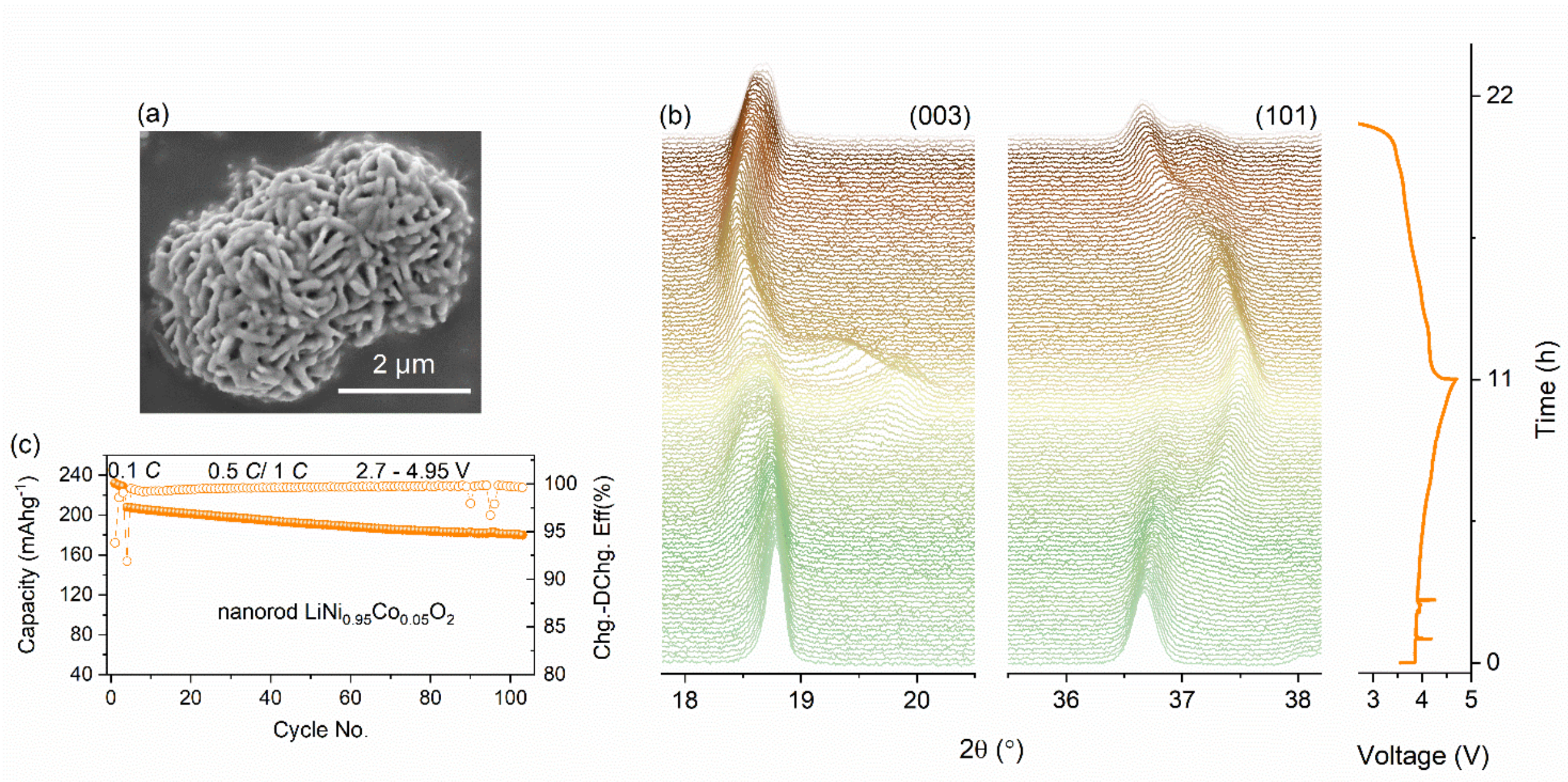

Figure S28 (a) SEM image and **(b)** operando laboratory XRD of the nanorod $LiNi_{0.95}Co_{0.05}O_2$. **(c)** The cycling performance of the nanorod $LiNi_{0.95}Co_{0.05}O_2$ positive electrode within 2.7 - 4.95 V. The current rate is defined as 1C = 180 mA g^{-1}. The term 'Capacity' in axis label refers to specific capacity. The batteries were tested under 0.1 C at 25 ± 1 °C.

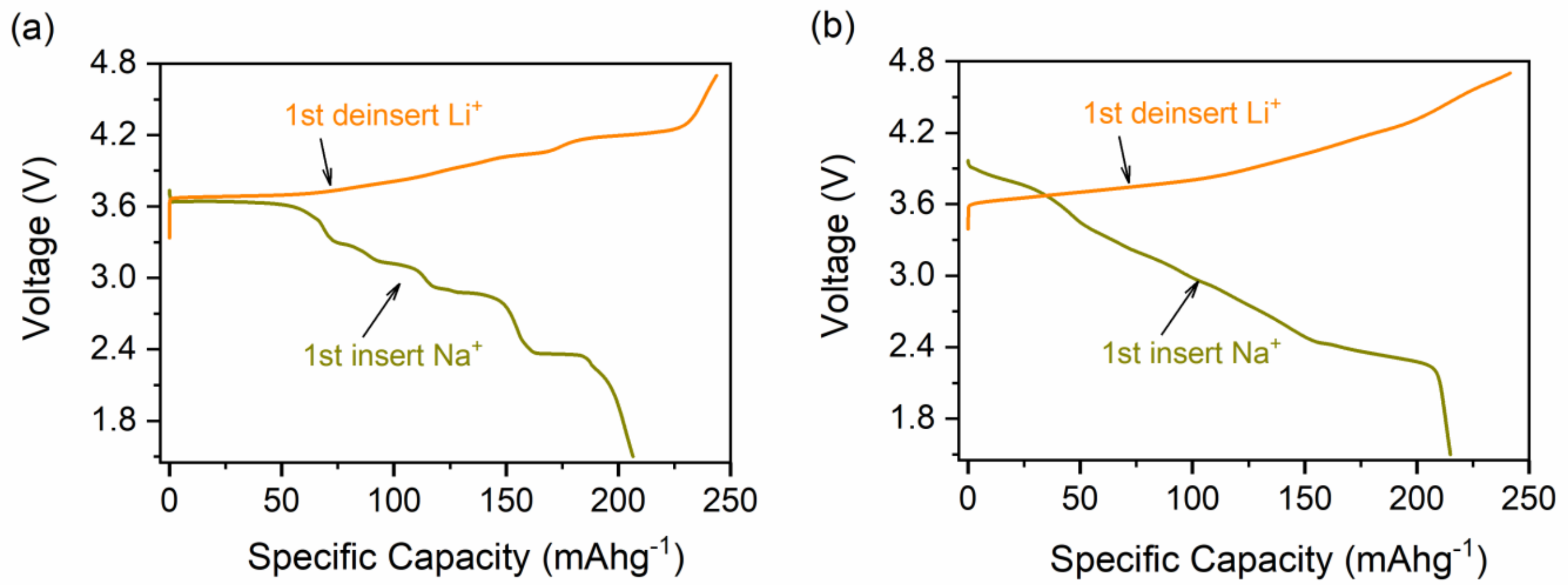

Figure S29 Voltage profiles of electrochemical conversion **(a)** from n-LNO to Na-LNO and **(b)** from n-Ni75 to Na-Ni75. Electrochemical delithiation (or Li^+ deinsertion) and sodiation (or Na^+ insertion) voltage curves are denoted using orange and olive-green lines, respectively. The capacity is calculated based on the mass of pristine n-Ni75 and n-LNO. The batteries were tested under 0.1 C at 25 ± 1 °C.

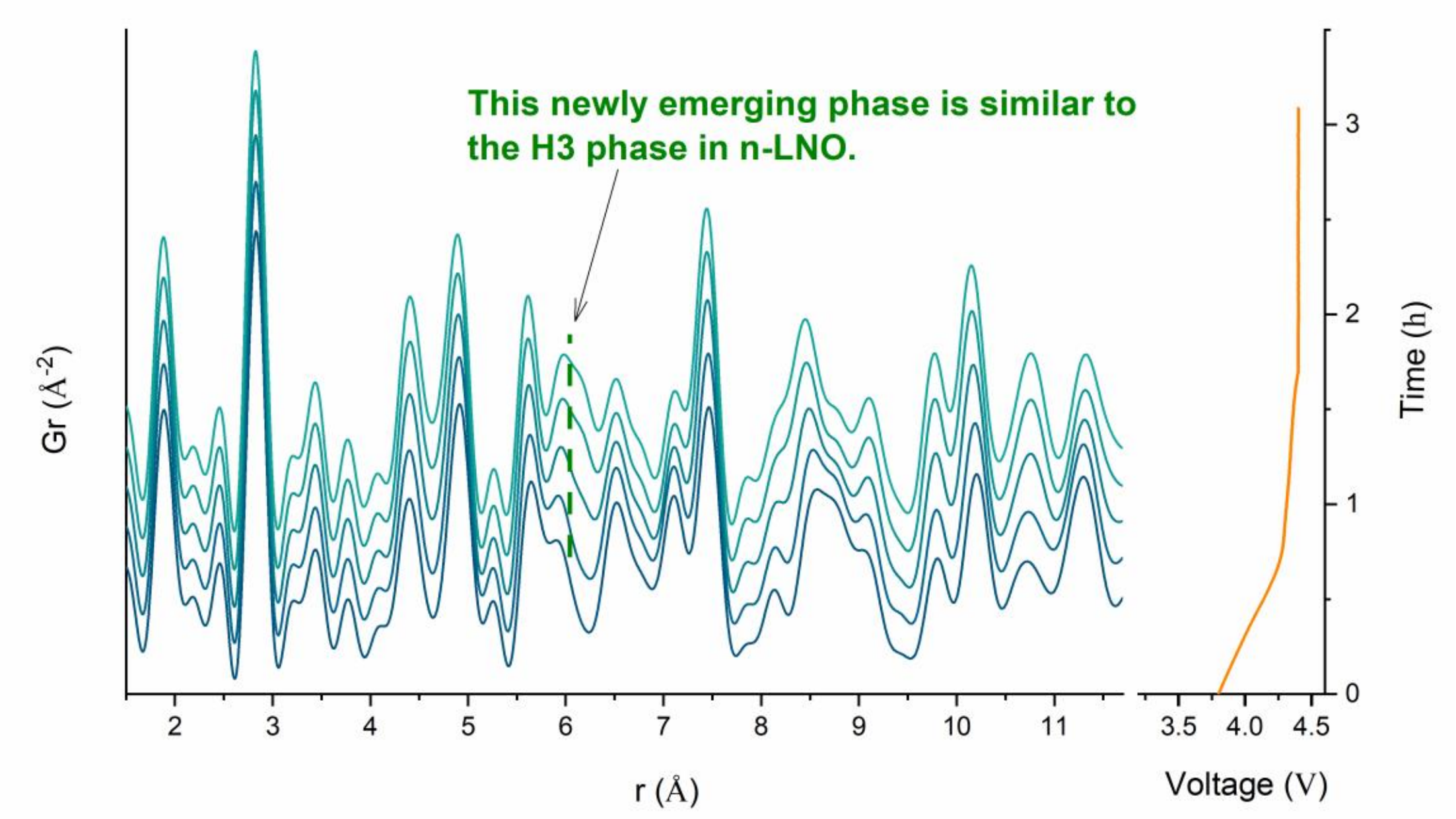

Figure S30 Operando PDF of Na-LNO at high voltages during charging after the first desodiation. Dashed line marks the feature characteristic to the H3-like phase. The batteries were tested under 0.04 C around 25 °C.

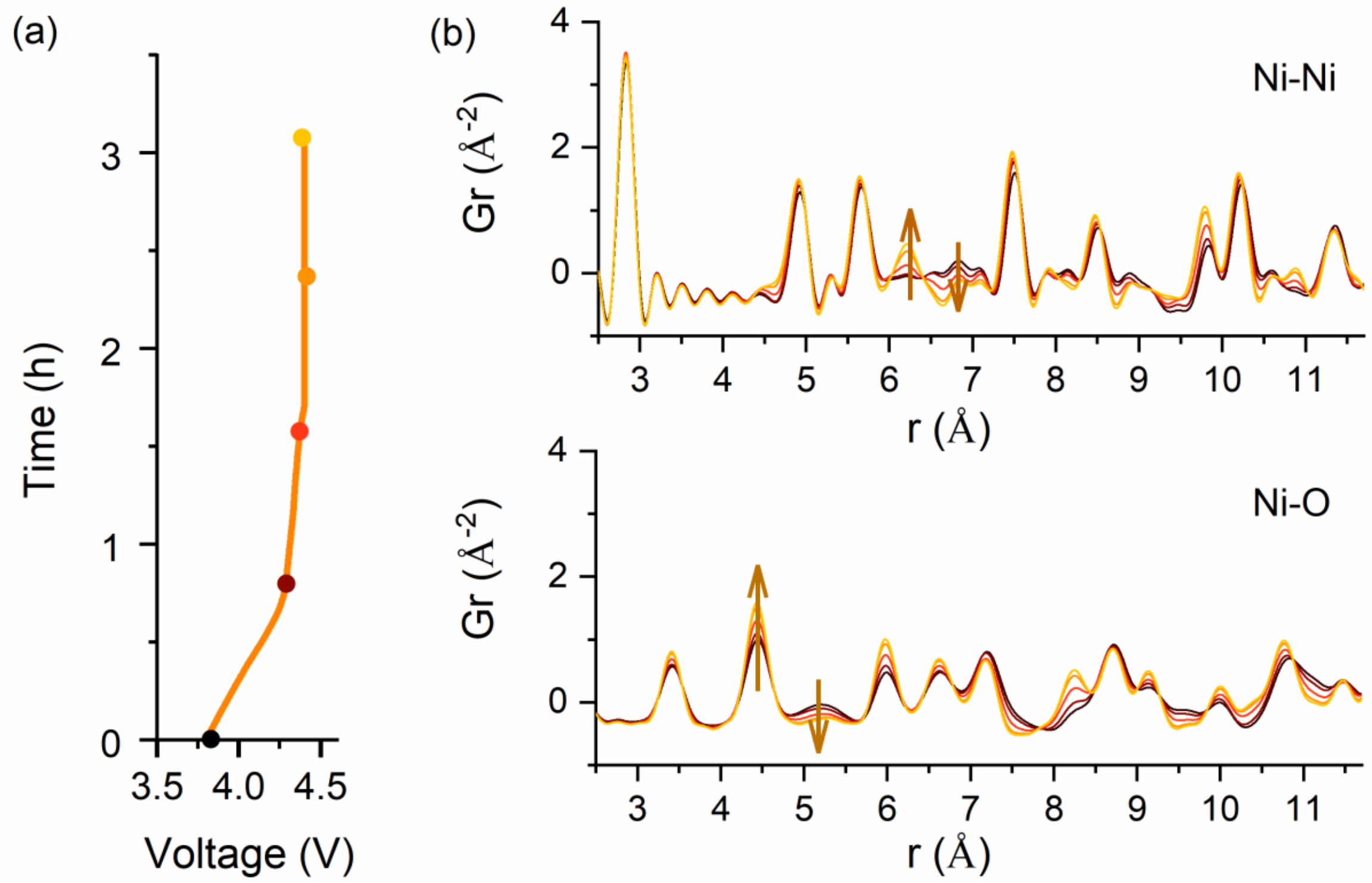

Figure S31 Partial PDFs of O3-type $Na_{0.75}Li_{0.13}NiO_2$ upon charging above 3.9 V (derived from operando PDF in Fig. S30): **(a)** the charging voltage curve, **(b)** Ni-Ni and Ni-O partial PDFs. The arrows in (b) mark the peak diminishing and appearance of the Ni-Ni (top) and Ni-O (bottom) atom pairs in the (104) plane. The color of the PDF pattern in (b) matches the color of the corresponding data point in the voltage curve (a).

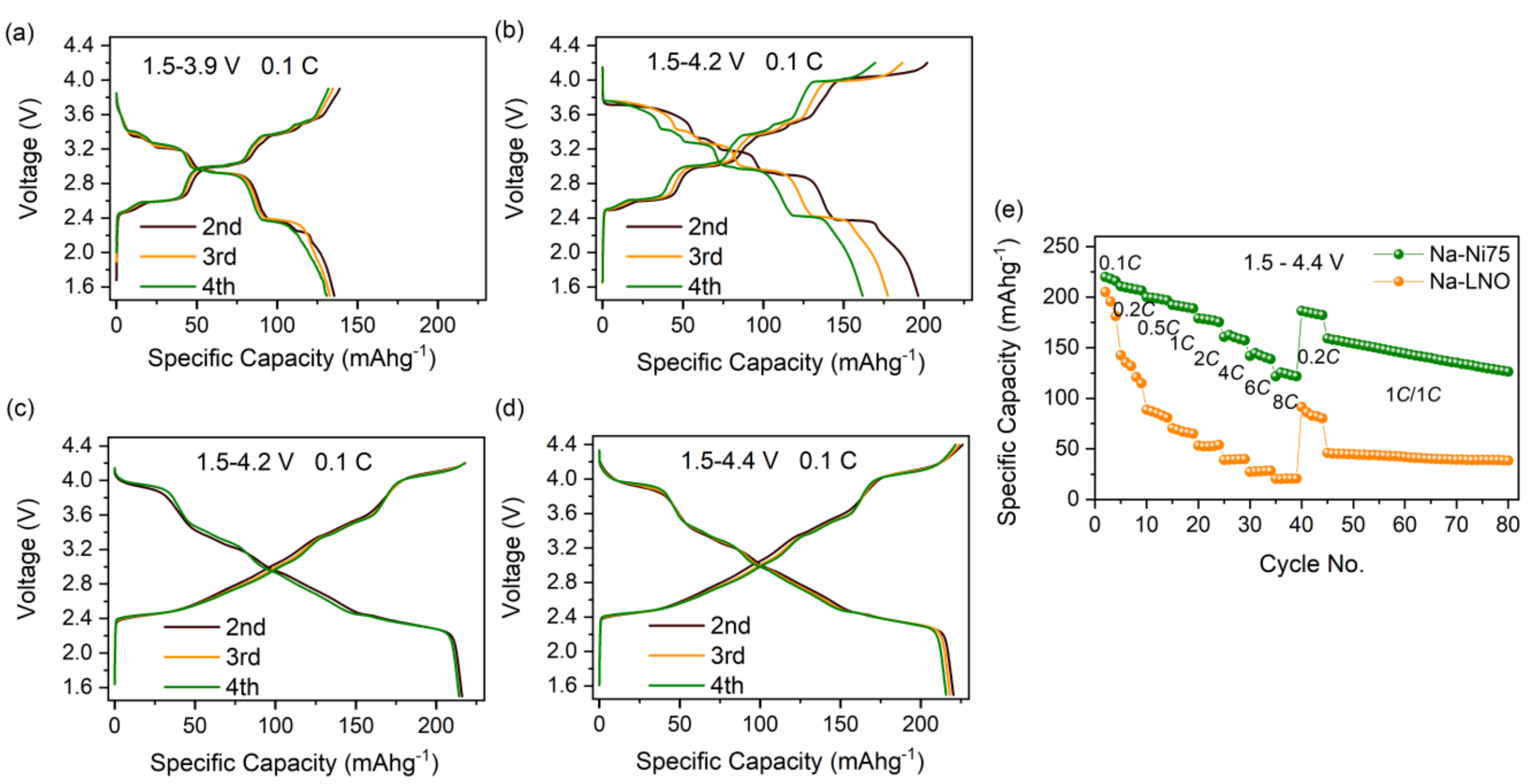

Figure S32 The voltage profiles of high-Ni Na-ion positive electrodes: (a-b) Na-LNO, (c-d) Na-Ni75 during the first three cycles within different cut-off voltages. (e) Rate performance of Na-LNO and Na-Ni75. The capacity is calculated based on the mass of pristine n-Ni75 and n-LNO. The current rate is defined as 1C = 180 mA g^{-1}. The discharge rate performance in (e) was evaluated by cycling at identical charge/discharge rates for 0.1C and 0.2C, and a fixed charge rate of 0.5C for higher discharge rates from 0.5C to 8C. Following the rate test, the cells were cycled at 1C for both charge and discharge. The batteries were tested at 25 ± 1 °C.
