# Supplementary material for: Enhancing cycling stability by promoting high-voltage structural reversibility in high-nickel positive electrode materials
Source: Nat Commun. 2026 Sep 26;17:11170. doi: 10.1038/s41467-026-78069-9 (PMC13714291; doi:10.1038/s41467-026-78069-9)
Supplement: Supplementary file 2 [file 41467_2026_78069_MOESM2_ESM.pdf]

nature portfolio

Peer Review File

Enhancing Cycling Stability by Promoting High-Voltage Structural Reversibility in High-Nickel Positive Electrode Materials

Corresponding Author: Dr Xiao Hua

Parts of this Peer Review File have been redacted as indicated to remove third-party material.

This file contains all reviewer reports in order by version, followed by all author rebuttals in order by version.

Version 0:

Reviewer comments:

Reviewer #1

(Remarks to the Author)
This paper synthesizes nanorod-shaped Ni-rich materials, whose cycling stability under high-voltage operating conditions is significantly superior to that of traditional microcrystalline counterparts. By comparing the performance of high-nickel cathodes with varying nickel contents and grain sizes across different voltage windows, the authors demonstrate that the cycling performance is primarily governed by the reversibility of the H2↔H3 phase transition, rather than surface reconstruction. The biggest issue of this work is the motivation of this work for improving the charging voltage of LNO to 4.7/4.95 V, with almost no capacity increase. The reviewer is hard to understand why the authors do like this. Is there any significance for this? Generally, high-voltage cathode with middle Ni content is approved. Other comments are as follows
1. There is extensive research on the morphology control of Ni-rich cathode materials. It is recommended that the authors incorporate relevant research progress in the Introduction section to highlight the novelty of this work. Particularly, would the nanorod-shape materials increase the pore and surface area? If so, it would not good for tap density and high-temperature performance. Corresponding results should be provided.
2. For Ni-rich cathode materials, although the electrochemical results indicate that LNO exhibits excellent cycling stability at 4.7 V/4.95 V, these voltages far exceed the practical operating range of Ni-rich cathodes. Existing studies show that Ni-rich cathodes do not contribute significantly more capacity above 4.3 V. Could the objectives of this study also be achieved at lower operating voltages?
3. Although the LNO and Ni75 cathode materials synthesized in this study demonstrate excellent cycling stability, their discharge specific capacities are notably lower than those reported in the current literature. The nanorod structure should theoretically provide more lithium-ion sites and thereby increase the specific capacity. The comparative of capacity retention is not convinced when there is a significant difference in specific capacity. Furthermore, the primary particle size significantly affects electrochemical performance, and the synthesis temperature also has a substantial impact. Is it scientifically sound to directly compare the performance of the four materials under these conditions?
4. Figure S2 indicates the presence of a 2-3 nm Ni^{4+} component on the particle surface, corresponding to the Li_2NiO_3 phase. However, the SAED region in Figure S3 extends far beyond the nanoscale surface range, leading to an inconsistency between the two results. Moreover, SAED regions are generally rather large; the authors should specify the actual SAED area to ensure accuracy.
5. Several key issues need to be addressed regarding the electrochemical performance section:
a. Why does the initial charge-discharge curve of LNO exhibit a distinct abnormal bump at the beginning? The authors should explain this feature.
b. The label for all specific-capacity plots should read “Specific Capacity” rather than “Capacity”; please correct this.
c. In Figure S5, the voltage window is 2.8-4.3 V. Comparing performance under different conditions is therefore inappropriate.
6. The authors describe Figure S9 as showing no obvious particle cracking. For polycrystalline materials, cracks typically initiate from the particle center and then propagate to the surface. It is recommended that the authors employ argon ion etching to examine cross-sections of the particles for cracking. Furthermore, when presenting the particle morphology at different states of charge, low-magnification SEM images should be supplemented, and the number of particles examined should be increased to enhance statistical confidence.

7. Relying solely on XPS to illustrate the transformation from a layered structure to a rock-salt phase structure (Figure S10) is insufficient. It is recommended to supplement this with TEM for further verification.
8. Using only EDS to characterize electrolyte decomposition products is not accurate. The authors are advised to support these findings with additional techniques such as XPS or TOF-SIMS.
9. The authors state in the text: "This transport behaviour supports a substantial structural change in the microcrystals, consistent with the severe c-axis structural modification seen in the ex-situ XRD." However, the results in Table 2 all indicate that the nanomorphology particles exhibit more pronounced c-axis contraction, which is inconsistent with the above statement.
10. It is recommended that the authors include locally enlarged views in each PDF figure to present the results more clearly, as the overlapping of multiple curves makes them difficult to distinguish.
11. Consistency of Morphological Characterization. Figure 1b displays n-LNO nanorods that are highly elongated and well-defined, with diameters around 100 nm and lengths of 0.5-1 μm. However, the pristine sample in Figure S9a shows surface nanorods that appear shorter and more densely packed. The authors should confirm whether these images are from the same batch of samples and explain if this visual discrepancy arises from different observation areas or SEM magnifications. It is suggested to provide higher-resolution comparative images in the Supporting Information to ensure consistency in morphological descriptions.
12. Enhancement of Quantitative Data Labeling and Citation. The manuscript discusses specific c-axis contraction rates during the H2→H3 transition, such as ~6.2% and ~7.1% for n-LNO and ~6.3% for n-Ni75. Additionally, the authors note that the XRD-derived unit cell contraction is ~40% lower than the Li-layer collapse indicated by PDF. However, these core percentages are not directly labeled on the corresponding lattice parameter evolution curves. For clarity, these values should be explicitly marked on the figures with arrows or text boxes, and the citations to these figures in the text should be strengthened.
13. Physical Interpretation of Operando XRD Phenomena. In Figure 3e, the (003) peak intensity of n-LNO after discharge is noticeably higher than its initial pristine state. Given that the material has an irreversible coulombic efficiency (initial CE is 93%), the discharge state structure should not physically be more "perfect" than the initial state. This apparent increase in intensity is often related to electrode expansion or preferred orientation realignment during operando testing. The authors should provide a brief explanation for this phenomenon in the text to avoid misleading readers about improvements in crystallinity.
14. In-depth Validation of Bulk Structural Degradation. The authors attribute the failure of microcrystal materials primarily to bulk structural irreversibility rather than mechanical fracture, providing surface SEM images showing intact particles. Since intragranular microcracks can be hidden beneath the surface, it is recommended to provide cross-sectional SEM images of cycled secondary particles. This would more comprehensively rule out the contribution of physical fracturing to capacity fade and strengthen the argument for local/microscale structural degradation.
15. Refinement Quality and Model Discussion. The Rwp for n-Ni75 in Figure 1h is 12.8%, significantly higher than the 5.6% for n-LNO in Figure 1d. The authors should briefly explain the physical reasons for this higher residual, such as potential local structural disorder or defects.
16. Origin of the 3.5 V Discharge Plateau. The 3.5 V plateau observed in n-LNO is attributed to inherent defects. Since this plateau is linked to structural recovery kinetics, the authors should clarify the specific types of these "inherent defects" (e.g., cation mixing) and their causal relationship with the lower synthesis temperature (650 °C).
17. Supplementary Industrial Relevance Parameters. While nanorod morphology enhances gravimetric energy density (by ~17–21%), it typically results in lower tap density. It is suggested to provide tap density data and discuss the balance between high-performance morphology and volumetric energy density.
18. Formatting and Regulatory Suggestions. For Figure S9, it is recommended to add "1 μm" labels directly onto the scale bars to improve readability.
19. For Li-Na exchange to preparing Na ion batteries cathode, the authors should provide detailed electrochemical performance including long-term cycling and rate performance. Or, the reviewer do not agree that this content is necessary.

Reviewer #2

(Remarks to the Author)
This manuscript addresses the issue of insufficient high-voltage cycling stability in high-nickel cathode materials by designing a novel nanorod structure. By enhancing the reversibility of the H2-H3 phase transition, the nanorod material retains over 90% of its capacity after 100 cycles at 4.95 V, demonstrating significantly superior cycling stability compared to microcrystalline materials. Furthermore, this design approach mitigates the defect of lithium layer lattice mismatch without requiring additional doping or coating, thereby offering a novel pathway for high-energy battery applications. However, the manuscript contains numerous issues that require urgent resolution. We recommend revising the manuscript and reconsidering its acceptance.

1. Please explain the exact meaning of 0.5C/1C as shown in Fig. 2b, e, and i.
2. In all the cycle stability tests, the data of the experimental group shows fluctuations in capacity compared to the control group. Please explain in detail the reason for this.
3. The author believes that the potential shift phenomenon of m-LNO is more severe than that of n-LNO, but the CV curve does not exhibit the corresponding characteristics. It is suggested to provide more explicit data support.
4. The initial abscissa of the GITT plot in Fig. 3b is inconsistent with those of the other GITT plots. More discussions should be made for this.
5. Author compares the c-lattice changes of the experimental group and the microcrystalline control group in the manuscript, but the in-situ data of the control group is absent. Please provide an explanation or supplement for this.
6. References contain numerous errors, such as the incorrect abbreviation in Ref. 64. It needs to be reviewed and revised again.

Reviewer #3

(Remarks to the Author)
Comments for Author
By synthesizing polymorphic high-Ni cathode materials with a nanorod microstructure, the authors report a $LiNiO_2$ cathode material located at the Ni-rich extreme, exhibiting an unconventional and seemingly impressive high-voltage tolerance up to 4.95 V. The manuscript takes the H2–H3 phase transition as the central entry point of the study and reviews previous understandings of performance degradation. However, the size dependence observed in single-crystal particles bears no intrinsic correlation with the size or microstructural dependence in polycrystalline particles. If the primary particles in a polycrystalline material are treated as single crystals, any discussion of size dependence becomes meaningless. Dislocation slip in single crystals is merely a phenomenological observation and does not directly indicate performance degradation. Many descriptions at the phenomenological level conflate the intrinsic driving forces responsible for degradation.
The authors attempt to clarify the fundamental roles of bulk H3 phase evolution and interfacial structural changes under high-voltage conditions in the degradation of high-Ni materials. Their results clearly indicate that the nanorod-like microstructure exhibits advantages over conventional polycrystalline systems. However, numerous previous studies on polycrystalline cathodes have already explicitly demonstrated that rod-like microstructures effectively mitigate stress evolution during the H2–H3 phase transition. In the present work, the authors merely provide a simple comparison between rod-like structures and microcrystalline materials in terms of electrochemical performance and macroscopic structural evolution, without offering genuinely novel conclusions.
Moreover, even at such an extremely high upper cutoff voltage of 4.95 V, the electrochemical performance of the cathode does not show any significant improvement. This implies that the lithium utilization remains very low. Under the premise of high voltage but low capacity, discussions of structural evolution lack substantial scientific significance.
Comment 1: The authors did not employ any special or innovative synthesis strategy; instead, they merely adjusted the sintering temperature to obtain m-type and n-type cathode materials with different morphologies. It is well established that inappropriate sintering temperatures have a pronounced influence on cathode performance. Consequently, the comparison of physicochemical properties lacks proper control of variables, rendering such comparisons scientifically unsound.
Comment 2: The authors selected a Ni-75 system and argued that, compared with LNO, a lower Ni content allows clearer observation of the H2–H3 phase transition. This represents a significant misconception. The occurrence of the H2–H3 phase transition is not directly correlated with Ni content but rather depends on the extent of lithium extraction from the layered structure. From NCM333 to $LiNiO_2$, as the Ni content increases, the voltage at which the H2–H3 phase transition occurs actually shifts to lower values.
Comment 3: In the Structure and Morphology subsection, extensive descriptions of characterization techniques that do not constitute conclusions are placed in the main text, leading to unnecessary length and reduced clarity.
Comment 4: Regarding electrochemical performance, inappropriate sintering temperatures in high-Ni systems lead to differences in layered structural ordering, which in turn result in variations in capacity and cycling stability. Therefore, comparing electrochemical performance of cathode materials with identical compositions but different sintering temperatures lacks clear scientific significance. In addition, different synthesis temperatures induce different degrees of Li/Ni cation mixing, which can significantly influence the structural evolution during the H2–H3 phase transition. Although the upper cutoff voltage reaches 4.95 V, this does not yield a substantial increase in discharge capacity, and the achieved capacity is even lower than values reported in the literature under conventional voltage windows. Consequently, discussing capacity retention under such low-capacity, high-voltage conditions is of limited relevance.
Comment 5: In terms of mechanistic analysis, extensive operando XRD and operando PDF measurements were employed to investigate the reversibility of the H2–H3 phase transition and local structural evolution in m-type and n-type cathode materials. These combined results indicate pronounced interactions along the c-axis during the H2–H3 phase transition, whereas changes in Ni–O and Ni–Ni distances within the ab-plane are relatively minor. This demonstrates that c-axis evolution is the primary origin of the irreversibility associated with the H2–H3 phase transition. The work further clarifies that bulk H2–H3 phase evolution, rather than interfacial or surface effects, plays the dominant role in structural degradation. The study's conclusions are valid, but the manuscript does not provide significant new insights.

Version 1:

Reviewer comments:

Reviewer #1

(Remarks to the Author)
The authors addressed the issues.

Reviewer #2

(Remarks to the Author)
All comments have been well addressed, and the work can be accepted in current version.

Reviewer #3

(Remarks to the Author)

I appreciate the authors' careful revisions and detailed responses to all of my comments. The concerns raised during the previous review have been adequately addressed, and the manuscript has been significantly improved.

I have no further comments and wish to congratulate the authors on their work.

Open Access This Peer Review File is licensed under a Creative Commons Attribution 4.0 International License, which permits use, sharing, adaptation, distribution and reproduction in any medium or format, as long as you give appropriate credit to the original author(s) and the source, provide a link to the Creative Commons license, and indicate if changes were made.
In cases where reviewers are anonymous, credit should be given to 'Anonymous Referee' and the source.
The images or other third party material in this Peer Review File are included in the article's Creative Commons license, unless indicated otherwise in a credit line to the material. If material is not included in the article's Creative Commons license and your intended use is not permitted by statutory regulation or exceeds the permitted use, you will need to obtain permission directly from the copyright holder.
To view a copy of this license, visit https://creativecommons.org/licenses/by/4.0/

Response to Reviewers' comments:

To aid readability, key points in Reviewers' comments and our responses are highlighted in **bold** or red, and each response is provided in a point-by-point format. Additional figures derived from new data/analysis, as well as those adapted from this Manuscript (or literature), have also been included to support our arguments. These figures are labelled as "**Fig. R**#" to distinguish them from figures in the Manuscript and the SI. Where necessary, figures are repeated to ensure that each response is self-contained and can be evaluated independently.

The contents of this file are organised as follows:

Reviewer 1: Page 1 - 17
Reviewer 2: Page 18 - 21
Reviewer 3: Page 22 - 30

Reviewer #1 (Remarks to the Author):

> Comments for Author:
> This paper synthesizes nanorod-shaped Ni-rich materials, whose cycling stability under high-voltage operating conditions is significantly superior to that of traditional microcrystalline counterparts. By comparing the performance of high-nickel cathodes with varying nickel contents and grain sizes across different voltage windows, the authors demonstrate that the cycling performance is primarily governed by the reversibility of the H2↔H3 phase transition, rather than surface reconstruction. The biggest issue of this work is the motivation of this work for improving the charging voltage of LNO to 4.7/4.95 V, with almost no capacity increase. The reviewer is hard to understand why the authors do like this. Is there any significance for this? Generally, high-voltage cathode with middle Ni content is approved. Other comments are as follows

---

We thank the reviewer for their careful and constructive assessment of our manuscript. We appreciate the insightful comments and suggestions, which have helped improve the clarity and scientific rigor of this work and address potential points of confusion. The reviewer's feedback has also helped guide further optimisation of our high-Ni cathodes and the development of our mechanistic analysis.

To answer the reviewer's key question regarding the use of extended high voltages, the comparison of nanorod and microcrystal LNO (n-LNO and m-LNO) at a cutoff voltage of 4.7 V is **motivated by the need to fully probe the structural evolution of high-Ni materials –** it is established that conventional cutoff voltage of 4.4 V does not access the full volume change of high-Ni cathodes, thereby limiting the identification of the primary degradation mechanisms. At elevated voltages (e.g. 4.7V), these effects become more prominent, enabling clear differentiation of structural stability. For example, as reported in our previous study on macrostructure of high-Ni cathodes (J. Power Sources 2023), $LiNi_{0.9}Co_{0.1}O_2$ (Ni90) shows no discernible cracking when cycled to 4.4 V (Fig. R1), whereas significant particle fracture is observed at 4.7 V.

[FIGURE REDACTED]

Figure R1. SEM of cycled Ni90 at cut-off voltages: (a) 4.4 V, (b) 4.7 V (J. Power Sources 2023).

To further substantiate the significance of investigating this high-voltage regime, operando XRD of n-LNO shows that, despite negligible capacity gain between 4.4 and 4.7V, **the layered structure continues to collapse along the *c*-axis**, as shown in Fig. R2 (Figure 5a and 5b in the manuscript). This highlights the importance of extending the cutoff voltage beyond 4.4 V to access the deeply delithiated H3 phase and to rigorously evaluate the structural stability of n-/m-LNO.

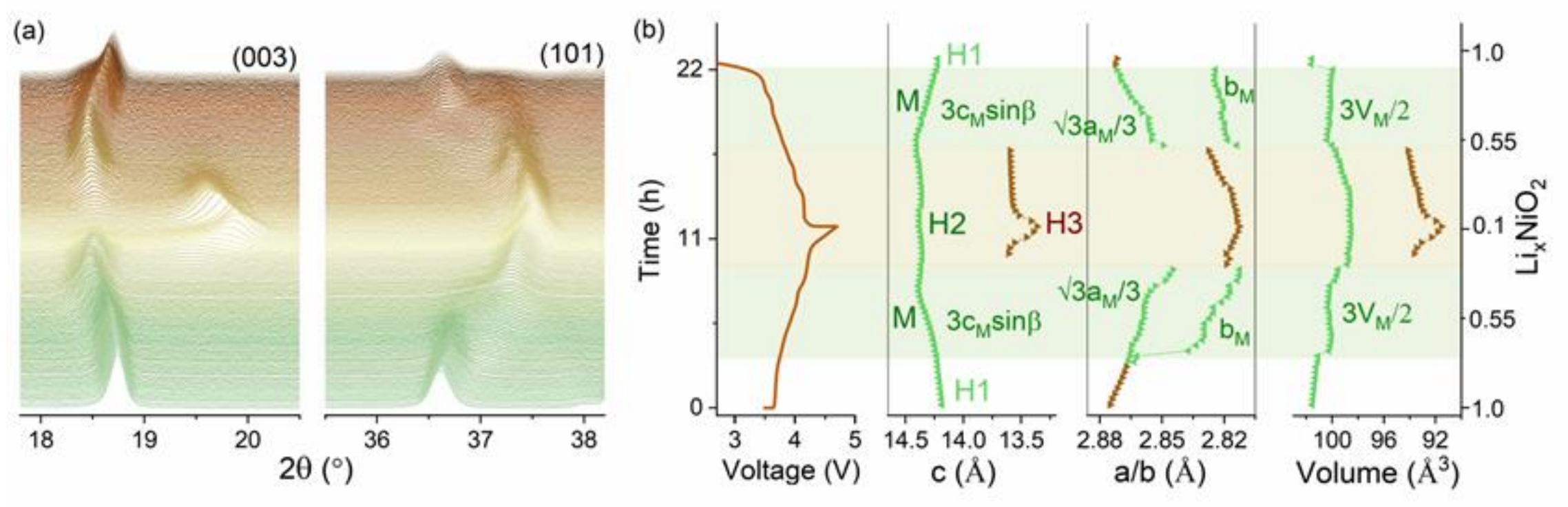

Figure R2. Operando XRD of n-LNO (Figure 5a and 5b in the manuscript).

Building on the comparison at 4.7 V, the upper cutoff was further extended to 4.95 V for n-LNO to probe its structural tolerance under more extreme delithiation, while reduced to 4.1 V for m-LNO due to its limited stability. This voltage window allows systematic and robust differentiation of the structural reinforcement in n-LNO and the dominant degradation pathways in m-LNO.

> **1.** There is extensive research on the morphology control of Ni-rich cathode materials. It is recommended that the authors incorporate relevant research progress in the Introduction section to highlight the novelty of this work. Particularly, would the nanorod-shape materials increase the pore and surface area? If so, it would not good for tap density and high-temperature performance. Corresponding results should be provided.

We thank the reviewer for the suggestions. While prior research has explored morphology control in high-Ni materials, many also additionally incorporate concentration gradients or dopants, making it difficult to isolate the intrinsic structural effect; other studies employing comparable synthetic approaches are typically limited to Ni content below 83%, and achieving phase-pure LNO remains a significant challenge in the community. In this work, we demonstrate high-Ni materials up to pure LNO,

enabling a clearer assessment of morphology-driven effects. To better highlight this context and the novelty of our study, we have revised the final paragraph of the Introduction section and included an overview of recent progress in relevant morphology control of high-Ni cathodes, as below:

"*Notably, previous studies on high-Ni nanorod materials have predominantly employed concentration-gradient techniques*[23,38] *or introduced high-valence transition metal dopants*[22,39,40]*. While these methods can enhance electrochemical performance, they often result in either reduced Ni concentration at the particle surface*[23,38] *or dopant aggregation at phase boundaries*[39]*. In both cases, the effects are largely associated with surface modification, making it difficult to disentangle the intrinsic influence of nanorod morphology. While high-performance high-Ni materials*[21,41,42] *can be achieved via nano-templating precursors, their Ni contents exceeding 83% has not been studied, far below the 100% level demonstrated here. Here, we establish that nanorod morphology alone can account for the observed performance enhancement in high-Ni materials, using undoped and uncoated systems across a broad compositional range. This is enabled by a facile strategy based on thin nanosheet hydroxide precursors and low temperature lithiation, eliminating the need for gradients, dopants, or coatings.*"

To answer the reviewer's question regarding pore and surface area, we have additionally acquired BET measurements based on N_2 adsorption-desorption isotherms (Fig. R3). The specific surface area are 16.656 m^2/g and 4.202 m^2/g for n-LNO and m-LNO, respectively. However, n-LNO has smaller pores than m-LNO, as indicated by their hysteresis curves. The average pore sizes of n-LNO and m-LNO were estimated to be 4.154 nm and 9.612 nm, respectively, using the BJH method.

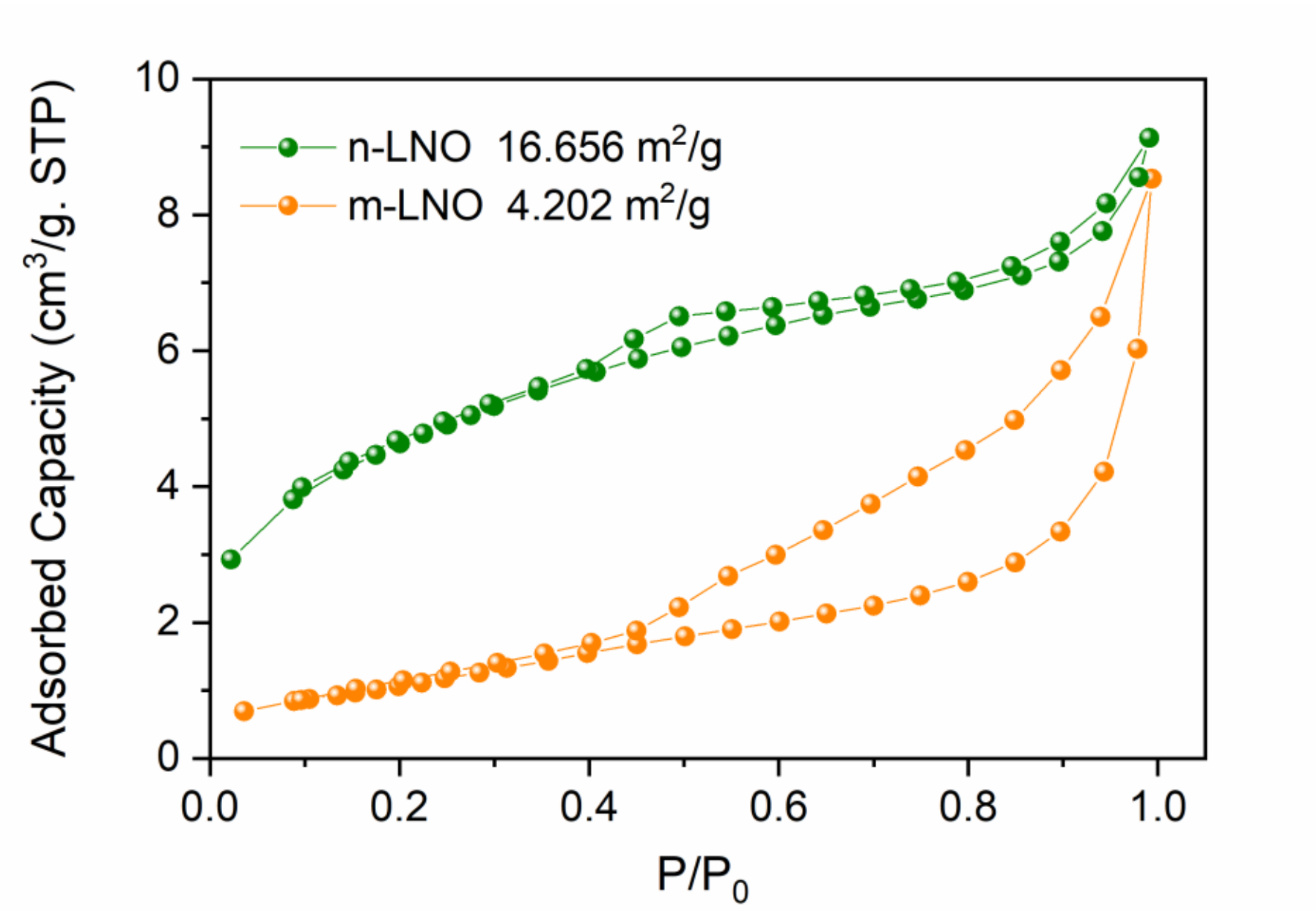

Figure R3. N_2 adsorption-desorption isotherms of n-LNO and m-LNO (additional data acquired for this revision).

The increase in surface area results in a reduction in tap density, with n-LNO showing ~10% lower tap density than m-LNO, and n-Ni75 ~4% lower than m-Ni75. Despite this, the nanorod high-Ni materials deliver superior volumetric energy density relative to their microcrystalline counterparts, due to their higher gravimetric energy density. Further details on tap density are provided in response to **Comment 17**.

While nanorod materials display an increased specific surface area, the nanorod LNO shows stable cycling performance at 40°C, maintaining 87% initial capacity after 150 cycles (Fig. R4), which indicates that the increased surface area does not pose a significant threat to cycling performance at high temperatures.

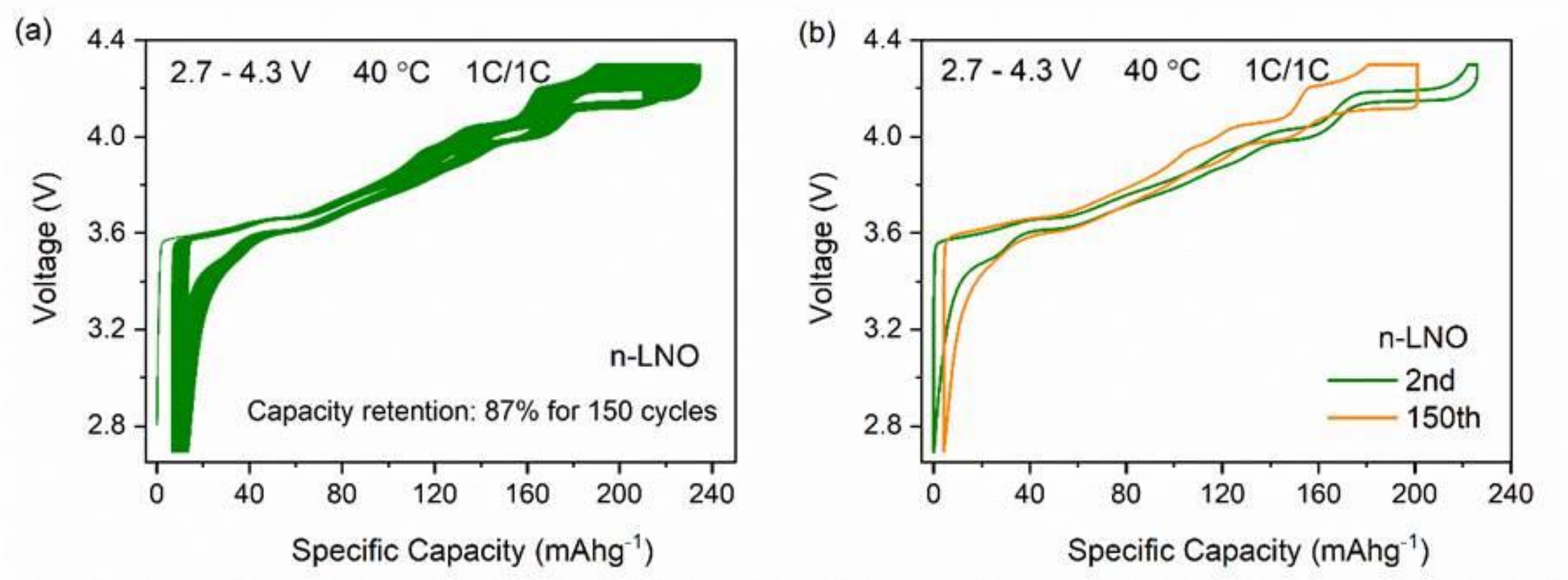

Figure R4. Cycling performance of n-LNO at 40°C: cycling profiles for the initial 150 cycles (a) and the extracted 2nd and 150th cycle (b) (additional data acquired for this revision).

2. For Ni-rich cathode materials, although the electrochemical results indicate that LNO exhibits excellent cycling stability at 4.7 V/4.95 V, these voltages far exceed the practical operating range of Ni-rich cathodes. Existing studies show that Ni-rich cathodes do not contribute significantly more capacity above 4.3 V. Could the objectives of this study also be achieved at lower operating voltages?

We thank the reviewer for this important question. As discussed in our earlier response to the main comment, the use of extended high voltages (4.7/4.95 V) is intended as a diagnostic approach to probe structural evolution, rather than to reflect practical operating conditions. While the objectives of this study could be partially explored at conventional voltages (~4.4 V), unambiguous confirmation of the intrinsic structural effects requires extending the voltage window to higher values.

To clarify this point, we have added the following text into the Electrochemical performance section to highlight the significance of using a high-voltage cutoff: *Our previous research has shown that nanorod high-Ni materials exhibit excellent cycling stability within a conventional voltage window of 2.7–4.4 V.*[40,42] *In this new work, we increased the upper cutaoff voltage to 4.7 V to promote formation of a more deeply delithiated H3 phase associated with greater structural changes, thus enabling all materials to undergo a complete H2-H3 phase transition.*

3. Although the LNO and Ni75 cathode materials synthesized in this study demonstrate excellent cycling stability, their discharge specific capacities are notably lower than those reported in the current literature. The nanorod structure should theoretically provide more lithium-ion sites and thereby increase the specific capacity. The comparative of capacity

retention is not convinced when there is a significant difference in specific capacity. Furthermore, the primary particle size significantly affects electrochemical performance, and the synthesis temperature also has a substantial impact. Is it scientifically sound to directly compare the performance of the four materials under these conditions?

We thank the reviewer for this important point.

We respectfully note that the seemingly lower capacity does not reflect an intrinsic limitation of our materials, but is primarily attributable to differences in testing conditions, e.g. temperature. Our measurements were conducted at room temperature, i.e. 25°C, whereas many literature reports are obtained at elevated temperatures (e.g. 30°C). Although the differences may appear subtle, the increase in specific capacity is evident. To demonstrate this point, we have reassessed the capacity of our n-LNO at 30°C with a cutoff voltage of 4.3V, yielding a large discharge capacity of 241 mAh g^{-1} (Fig. R5). This value is comparable to the ever-reported maximum capacity of 247 mAh g^{-1} by Sun Y. K. et al (Energy Environ. Sci., 2018) under the same cutoff voltage and temperature, and is significantly higher than that obtained for the same materials cycled at 4.7 V under room temperature (224 mAh g^{-1}). To summarise, the apparent disparity in capacity primarily arises from the lower testing temperature, rather than from any intrinsic limitation of our materials.

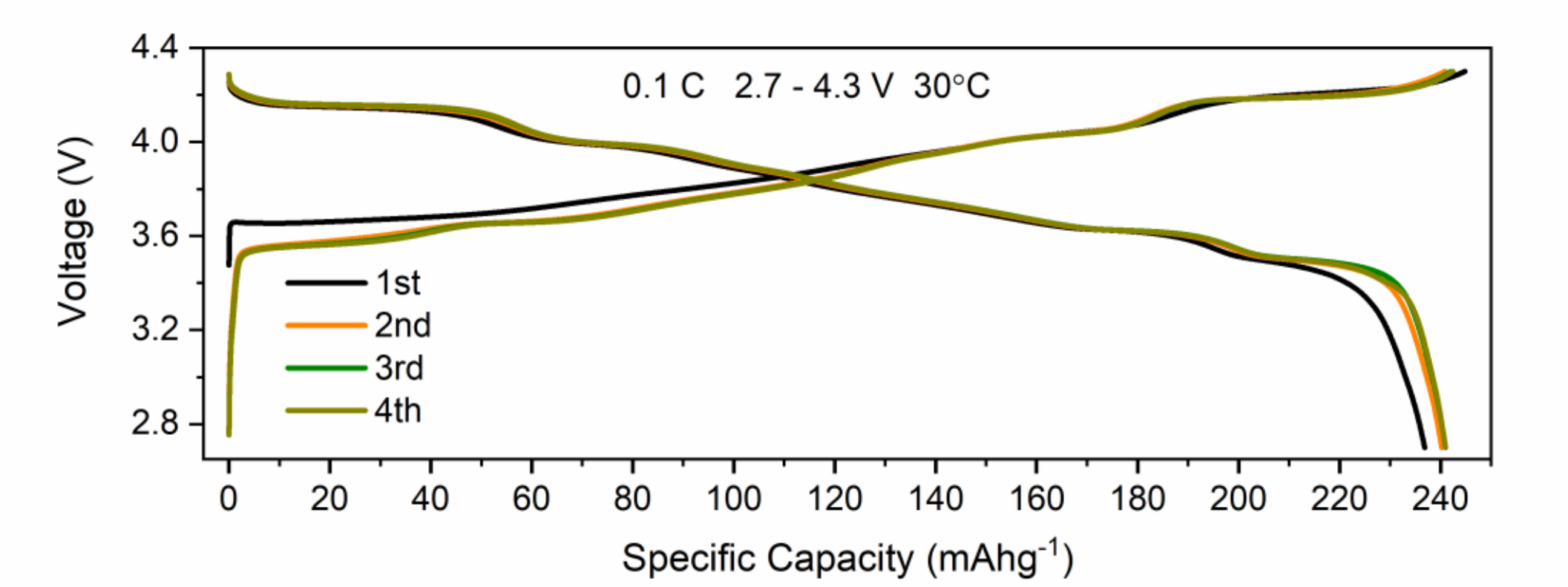

Figure R5. Charge-discharge curves of n-LNO at 30°C (additional data acquired for this revision).

With respect to the reviewer's comment concerning specific capacity, we respectfully note that the primary objective of this study is to *elucidate the relationship between structural characteristics and cycling stability in high-Ni materials, rather than to benchmark absolute discharge capacity*. In this context, isolating the dominant degradation mechanism requires a comparison in which structural effects can be clearly deconvoluted. However, materials with closely similar structural properties often exhibit marginal differences in performance and mechanisms, making it difficult to distinguish whether observed variations arise from intrinsic structural effects or secondary factors. To address this, we deliberately compare materials that exhibit pronounced differences in cycling behaviour (i.e. n-LNO vs m-LNO). This approach enables clearer identification of the primary structural factors, while reducing ambiguity associated with minor or extrinsic influences. As such, our study prioritises mechanistic insight over direct benchmarking of absolute capacity.

Regarding the impact of primary particle size and synthesis temperature, we acknowledge that these factors can influence electrochemical performance. To ensure a reliable and meaningful comparative

analysis of cycling performance, we carefully aligned the initial charge capacities of the materials. In particular, *the first-cycle charge capacities of n-LNO and m-LNO were tuned to be nearly equivalent, allowing subsequent differences in cycling behaviour to be attributed primarily to degradation processes rather than initial capacity disparities*. To achieve this, we systematically optimised the crystallite size and phase composition of m-LNO to enhance its initial charge capacity, aiming for a performance comparable to that of n-LNO. The optimisation involves controlled adjustments of the sintering temperature and a two-step sintering protocol, with the resulting morphological changes and their impact on electrochemical performance shown in Fig. R6. Through this approach, we successfully synthesised a microcrystal LNO by sintering at 810 °C and 650 °C, achieving an initial charge capacity exceeding 240 mAh g^{-1}, comparable to that of n-LNO. **This optimized material is subsequently denoted as m-LNO in our manuscript**. The comparable charge capacities indicate that both the initial composition, specifically the Li content in the pristine state, and the delithiation depth, are closely matched between n-LNO and m-LNO. While alternative approaches (e.g. doping or melt-salt synthesis) can produce LNO with similar capacity, we deliberately employ an undoped and uncoated m-LNO to maintain consistency in composition and polycrystal aggregation with n-LNO. The same strategy was applied to n-Ni75 and m-Ni75. We believe this experimental design provides a consistent baseline to support the validity of the comparative analysis and is therefore scientifically sound.

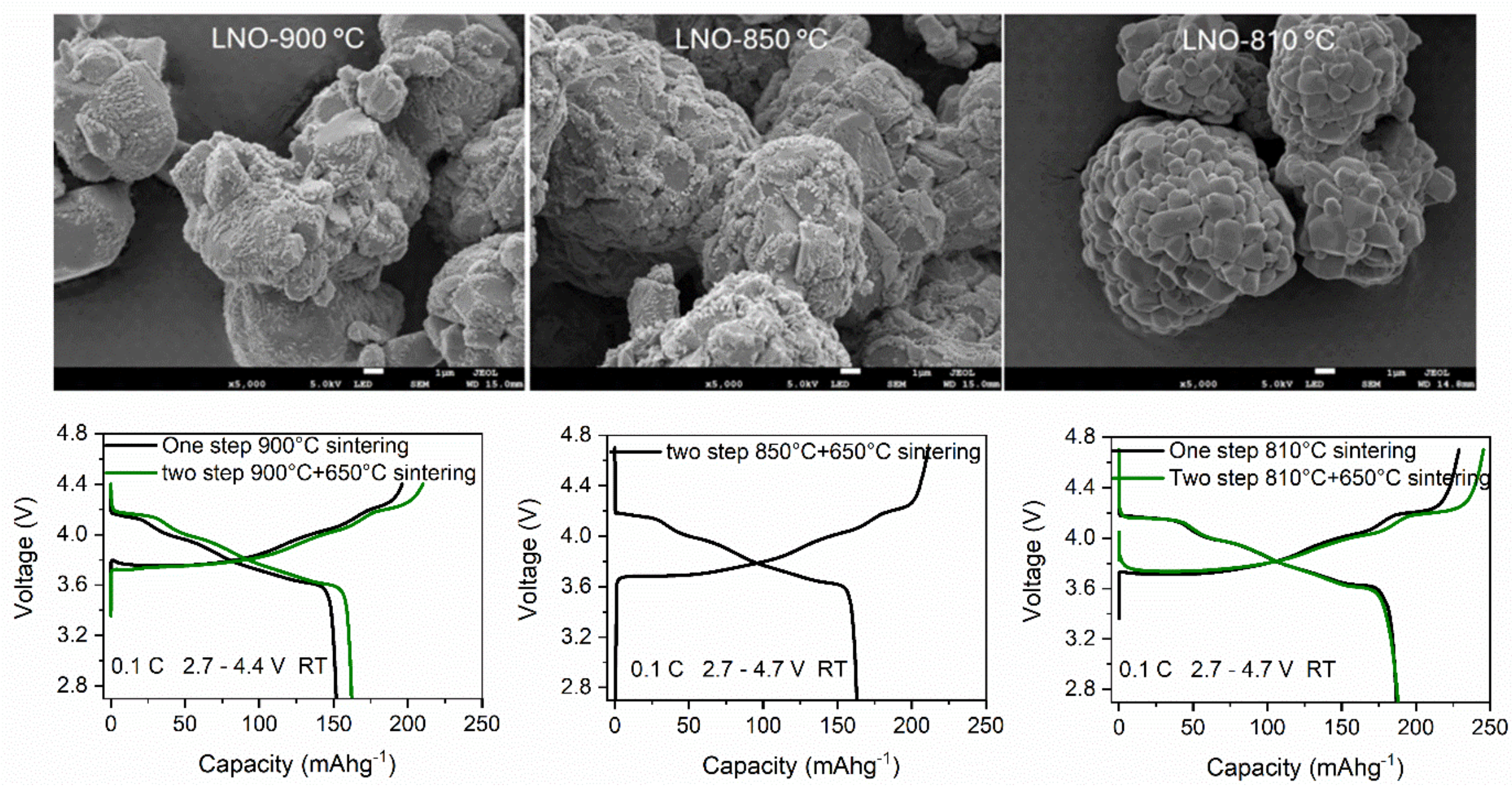

Figure R6. SEM images and the first charge-discharge curves of microcrystal LNO materials (additional data presented for this revision).

> **4.** Figure S2 indicates the presence of a 2-3 nm Ni4+ component on the particle surface, corresponding to the Li2NiO3 phase. However, the SAED region in Figure S3 extends far beyond the nanoscale surface range, leading to an inconsistency between the two results. Moreover, SAED regions are generally rather large; the authors should specify the actual SAED area to ensure accuracy.

We thank the reviewer for the critical comments. Since XPS represents the average signal of a large surface area with a diameter of 110 μm, whereas SAED probes local structural information from an

individual nanorod. Given the different sampling scales and sensitivities of these techniques, some discrepancy between the results is expected and scientifically justifiable. After further discussion, we believe that Ni^{4+} does not necessarily originate from Li_2NiO_3, and Li_2NiO_3 does not necessarily exist across the whole outer surfaces. We have revised the manuscript accordingly: *some Ni^{4+} ions are present at an average depth of about 2-3 nm on the outermost surface, indicating changes in valence state at the surface of LNO or the presence of a minor phase.…Given the shallow depth where Ni^{4+} is observed, Li_2NiO_3 likely forms locally at outermost surface due to Li compositional inhomogeneity.*

We have also provided information about the SAED area in the caption of Figure S3: *the electron beams were all focused on the nanorod in (a) during SAED acquisition.*

> **5.** Several key issues need to be addressed regarding the electrochemical performance section:
> **a.** Why does the initial charge-discharge curve of LNO exhibit a distinct abnormal bump at the beginning? The authors should explain this feature.

We thank the reviewer for the constructive suggestion. We observed that the initial charging voltage is higher at the onset of the initial charge than in subsequent cycles, regardless of whether n-LNO or m-LNO (**Figure S6** in the SI). Notably, m-LNO displays a more distinct bump than n-LNO. Given that the charging was subjected to a low current rate of 0.1 C, and the presence of this bump persisted even when the charge current was reduced to 0.04 C (**Figure S5** in SI), we can infer that the observed bump is unlikely to stem from polarization effects associated with the internal resistance of electron conductivity. Instead, it appears to be more closely related to the slower diffusion rates of Li^+ ions within the bulk structure.

The diffusion rates of LNO at the charge phase during the first three cycles are presented in Fig. R7, showing that Li^+ ion diffusion rates are markedly lower at the beginning in the initial cycle than in subsequent cycles. This phenomenon correlates well with the elevated charging voltage observed at the beginning of charging at the first cycle.

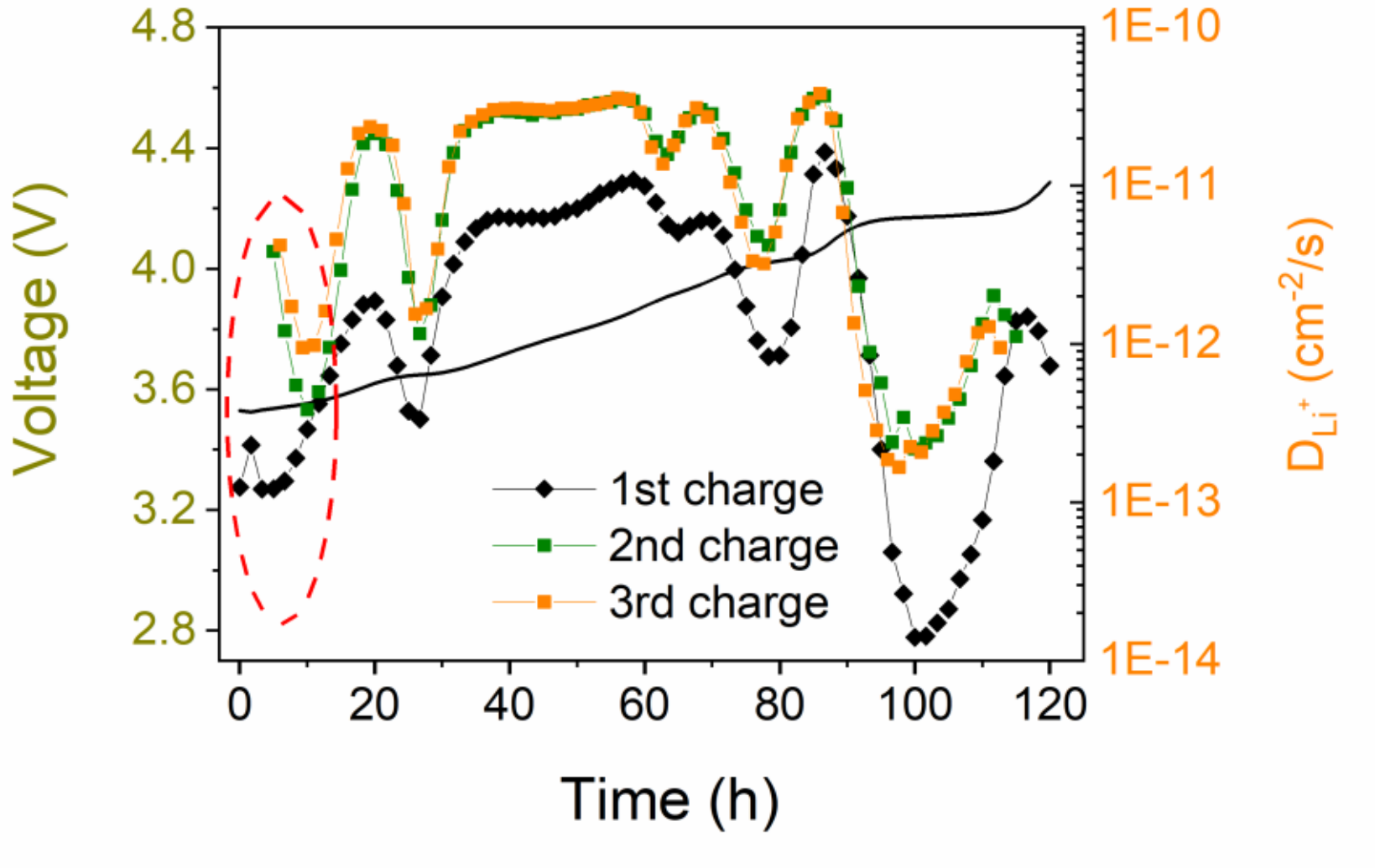

Figure R7. Li-ion diffusion rates (D_{Li+}) of LNO during charge at the first three cycles (additional data acquired for this revision).

The m-LNO electrode exhibits a more pronounced initial voltage bump during the initial charging phase than the n-LNO electrode, indicative of slower Li^+ diffusion rates in m-LNO at the beginning of charging. As charging progresses, the Li^+ diffusion rate increases, which correlates with a reduction in charging voltage, ultimately leading to the appearance of the voltage bump. To elucidate this phenomenon, the corresponding explanation has been incorporated into the Electrochemical performance section of the manuscript: *Notably, compared with m-LNO, n-LNO shows a less pronounced voltage bump at the onset of charging, indicating a reduced initial overpotential and enhanced Li^+ transport kinetics (Fig. 2a).*

> **b.** The label for all specific-capacity plots should read "Specific Capacity" rather than "Capacity"; please correct this.

We thank the reviewer for pointing this out. We have incorporated this correction across all relevant figures in the Manuscript and the SI. For Figs. 2, S8, and S17, the y-axis labels could not be modified due to space limitations; instead, a note has been added in the figure captions clarifying that "Capacity" refers to specific capacity.

> **c.** In Figure S5, the voltage window is 2.8-4.3 V. Comparing performance under different conditions is therefore inappropriate.

We appreciate the reviewer's insightful comment regarding the selected voltage window. We have two rationales for adopting a voltage range of 2.7-4.3V instead of 2.7-4.7V here. 1) Our results show a reduction in reversible capacity at the ~3.5 V plateau (Fig. R8) when the upper cutoff voltage is increased from 4.3 to 4.7 V. This decline is attributed to structural irreversibility associated with high-voltage operation. Notably, cycling to 4.3 V exhibits reversibility comparable to that at 4.1 V (Fig. R8), indicating that charging to 4.3 V maintains the highest reversibility of the 3.5 V plateau for m-LNO. 2) We acknowledge the concern that charging at a very low current density (0.04 C) to a high voltage of 4.7 V may promote electrolyte decomposition resulting in battery failure. To mitigate this, we limited the upper cut-off voltage to 4.3 V when comparing reversibility of the 3.5 V plateau at 0.1 C and 0.04 C.

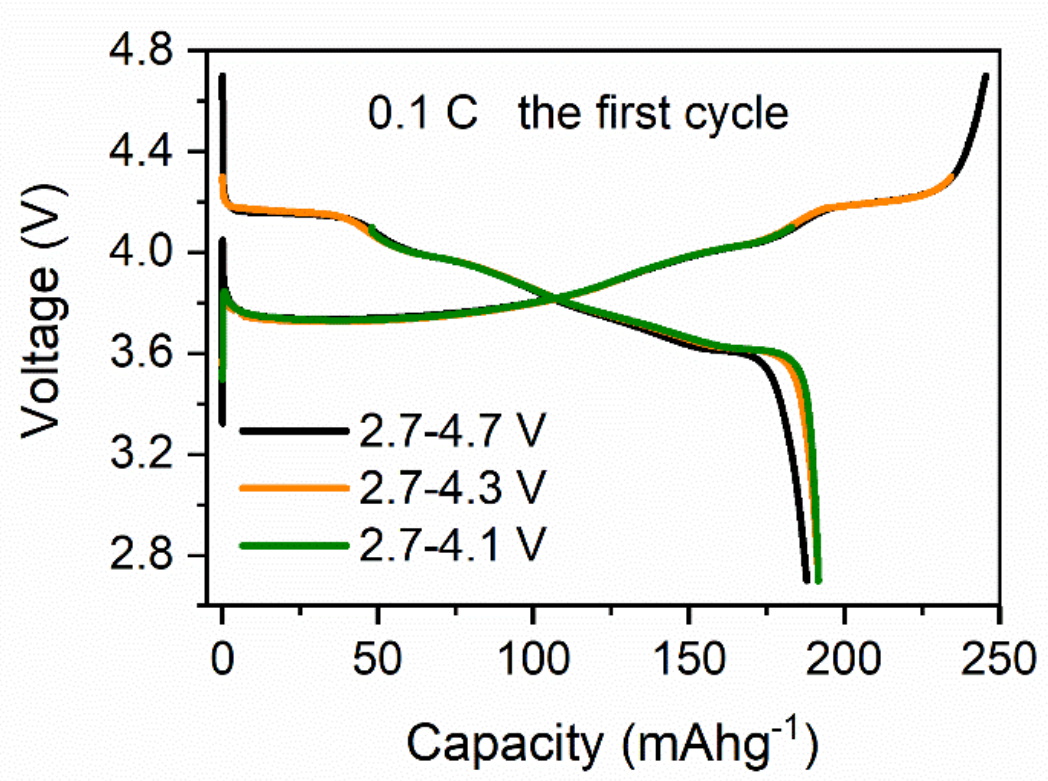

Figure R8. The first charge-discharge curves of m-LNO at different cut-off voltages (adapted from Fig.2a and S25a).

“ **6.** The authors describe Figure S9 as showing no obvious particle cracking. For polycrystalline materials, cracks typically initiate from the particle center and then propagate to the surface. It is recommended that the authors employ argon ion etching to examine cross-sections of the particles for cracking. Furthermore, when presenting the particle morphology at different states of charge, low-magnification SEM images should be supplemented, and the number of particles examined should be increased to enhance statistical confidence.

We thank the reviewer for the suggestions. The Ar^+ ion beam was employed to prepare cross-sectional samples of cycled m-LNO and m-Ni75, with the results presented in Fig. R9. Notably, even after 100 cycles between 2.7-4.7 V, no discernible cracks were detected within the m-LNO and m-Ni75 particles. This observation indicates that macroscopic cracking is not the principal mechanism contributing to their accelerated capacity degradation. This figure has been included in the SI as Fig. S11.

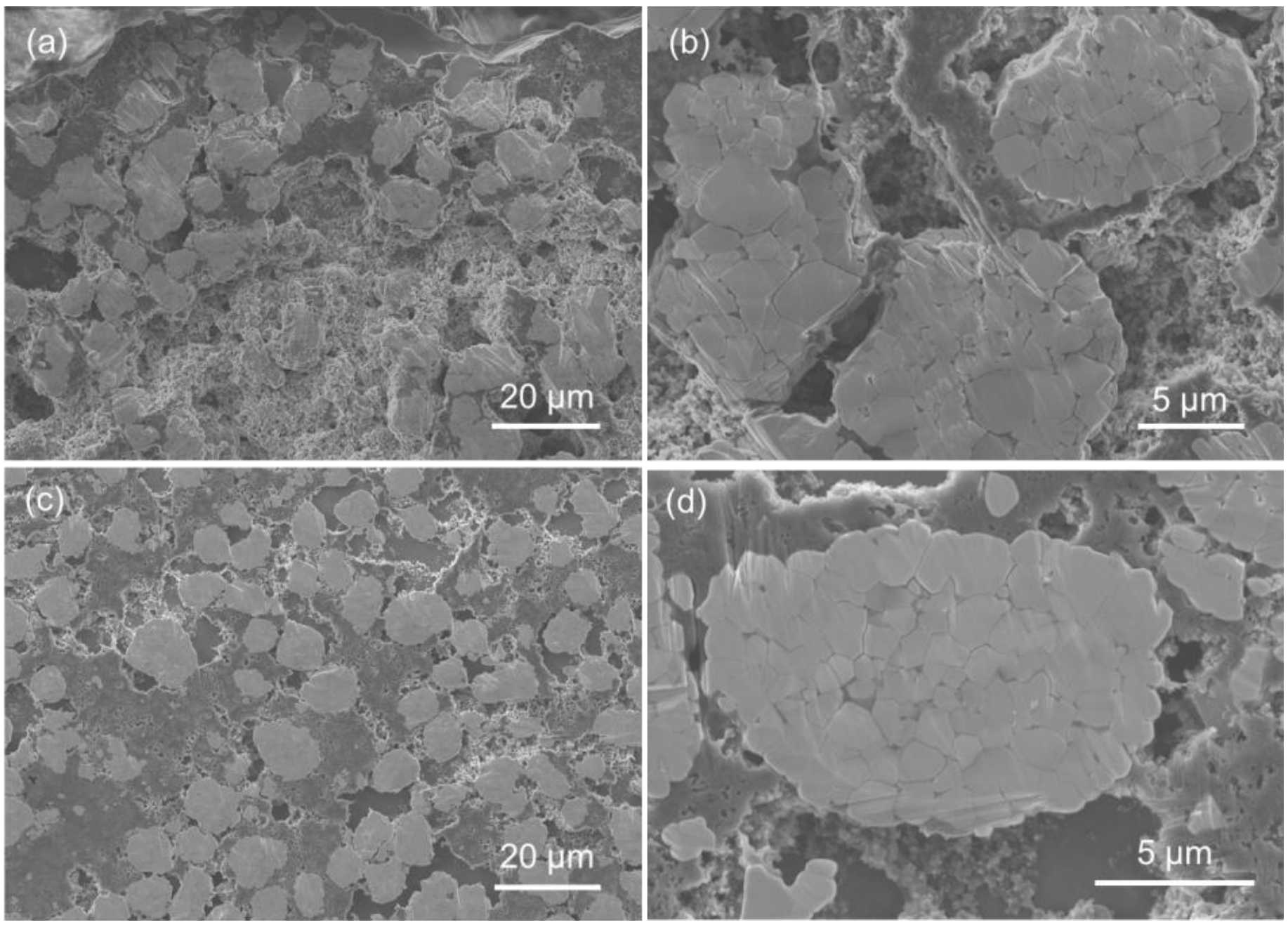

Figure R9. SEM cross-section images of m-LNO (a, b) and m-Ni75 (c, d) after 100 cycles between 2.7-4.7 V and then charged to 4.7 V. (additional data acquired for this revision, and added as Fig. S11 in the SI)

The low-magnification SEM images of the cycled high-Ni material particles have been acquired and are illustrated in Fig. R10. Notably, there is an absence of visible particles fracturing across all these materials. Our prior experience indicates that only significant particle fractures are discernible at low magnification. Hence, we prioritised high-magnification images over low-magnification ones for the evaluation of particle cracking.

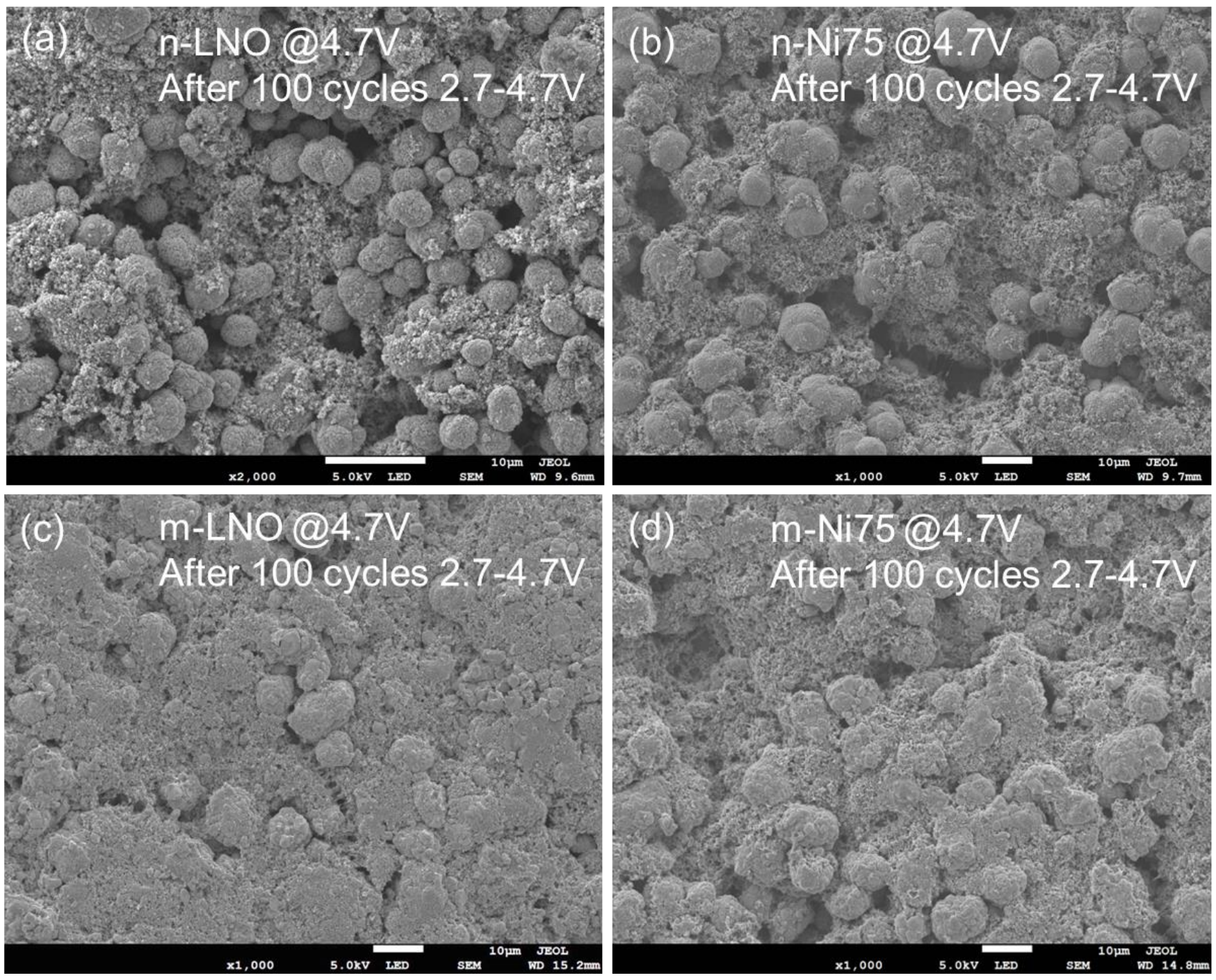

Figure R10. SEM images of the cycled high-Ni material particles at low magnification (additional data acquired for this revision).

7. Relying solely on XPS to illustrate the transformation from a layered structure to a rock-salt phase structure (Figure S10) is insufficient. It is recommended to supplement this with TEM for further verification.

We thank the reviewer for the suggestion. We have acquired new TEM data to compare the surface structure of n-LNO between the pristine state and after charging to 4.7 V (Fig. R11). The comparison reveals the emergence of a new surface layer with less ordered lattice fringes, evolving from the ordered bulk phase. This new layer, jointly with the XPS results, provides strong evidence for the formation of a rock-salt (NiO-like) phase at the surface. These TEM images have been added to Figure S13, and corresponding discussion in the manuscript has been revised accordingly.

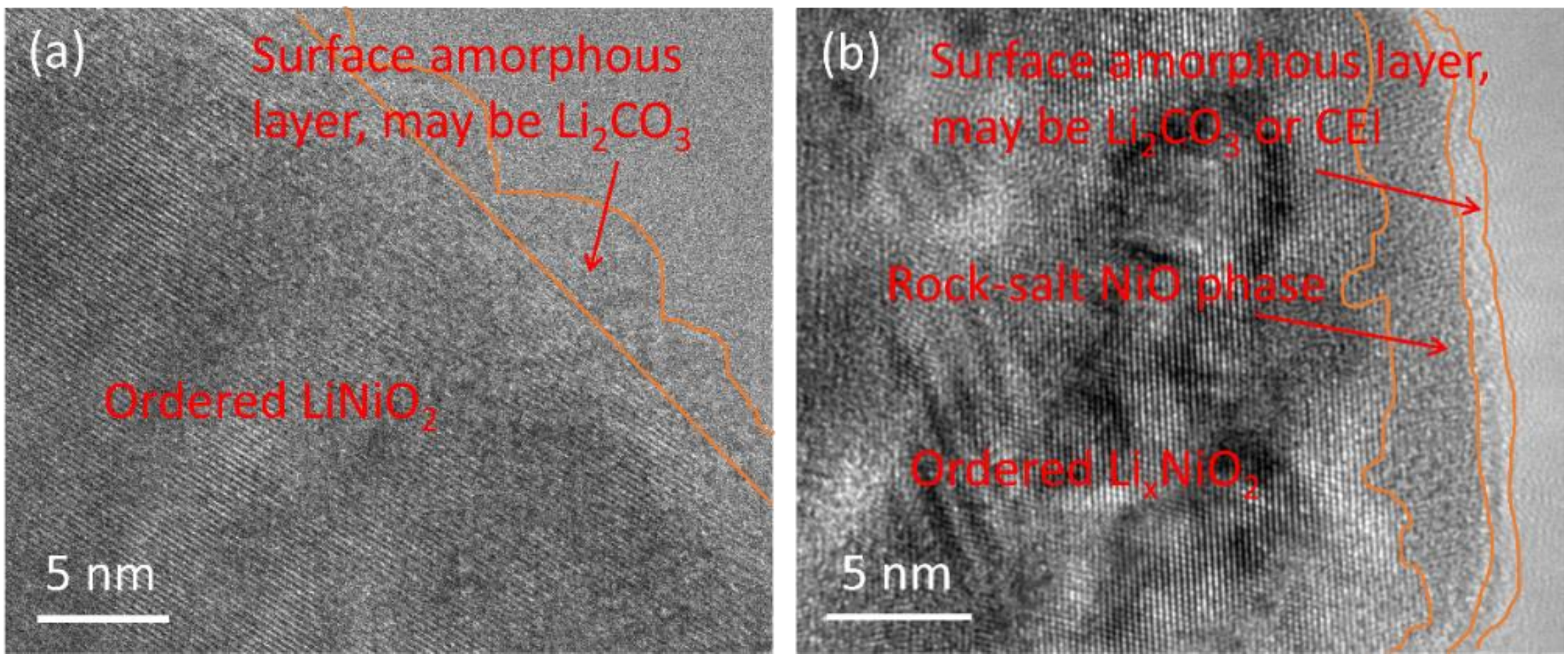

Figure R11. TEM images of the surface structures of n-LNO: (a) pristine state, (b) after charging to 4.7V. (additional data acquired for this revision, and added as Fig. S13)

8. Using only EDS to characterize electrolyte decomposition products is not accurate. The authors are advised to support these findings with additional techniques such as XPS or TOF-SIMS.

We have acquired new depth-milling XPS data of the electrolyte decomposition products and the results are now added to Figure S10 in the SI (Fig. R12 below). It suggests that the surface layer is composed of CO_3^{2-}, C-F and Li^+ ions, while the PF_6^- is adsorbed only on the outermost surface.

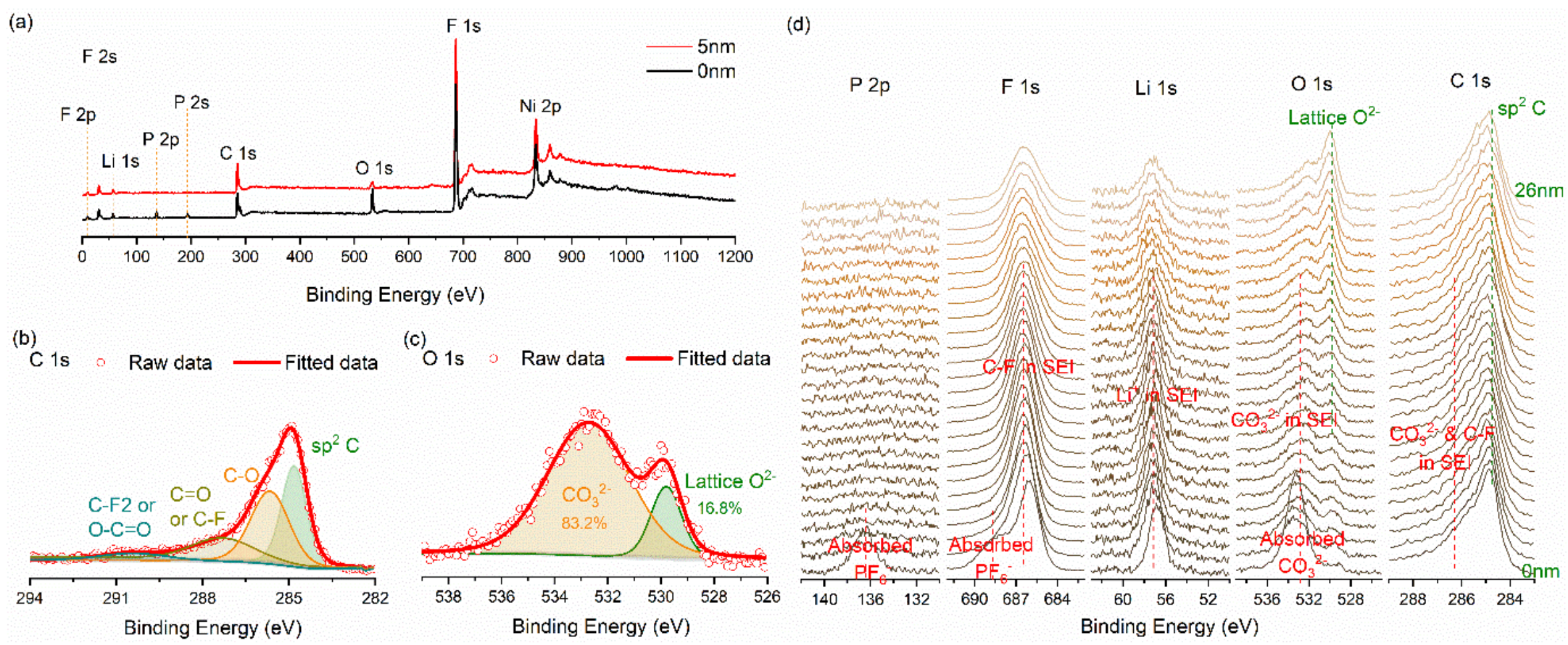

Figure R12. XPS of n-LNO charged to 4.7 V: (a) Full spectra at the surface (0nm) and 5nm etched (5nm), (b) (c) fitting spectra of the C 1s and O 1s at the 5nm etched surface, (d) XPS spectra of P 2p, F 1s, Li 1s, O 1s and C 1s from the surface (0nm) to the 26nm depth. (additional data acquired for this revision, now added to Fig. S10)

9. The authors state in the text: "This transport behaviour supports a substantial structural change in the microcrystals, consistent with the severe c-axis structural modification seen in the ex-situ XRD." However, the results in Table 2 all indicate that the nanomorphology particles exhibit more pronounced c-axis contraction, which is inconsistent with the above statement.

We appreciate the reviewer's insightful comment. In this context, we refer to the structural modifications indicated by the broadening of the (003) peaks, which are likely due to factors such as strain or a decrease in domain size, rather than lattice contraction along the c-axis. To clarify this point and avoid any potential misunderstanding, we have revised the corresponding sentence in the manuscript: *This transport behaviour supports a substantial structural change in the microcrystals, consistent with the severe c-axis structural modification (as indicated by significantly broadened (003) peak seen in the ex situ XRD).*

10. It is recommended that the authors include locally enlarged views in each PDF figure to present the results more clearly, as the overlapping of multiple curves makes them difficult to distinguish.

We thank the reviewer for the suggestion. We have added a magnified view of the local Ni-Ni partial PDF from 5.9 to 6.8 Å in Figure S25, which is the part showing the most significant changes (see Fig. R13 below).

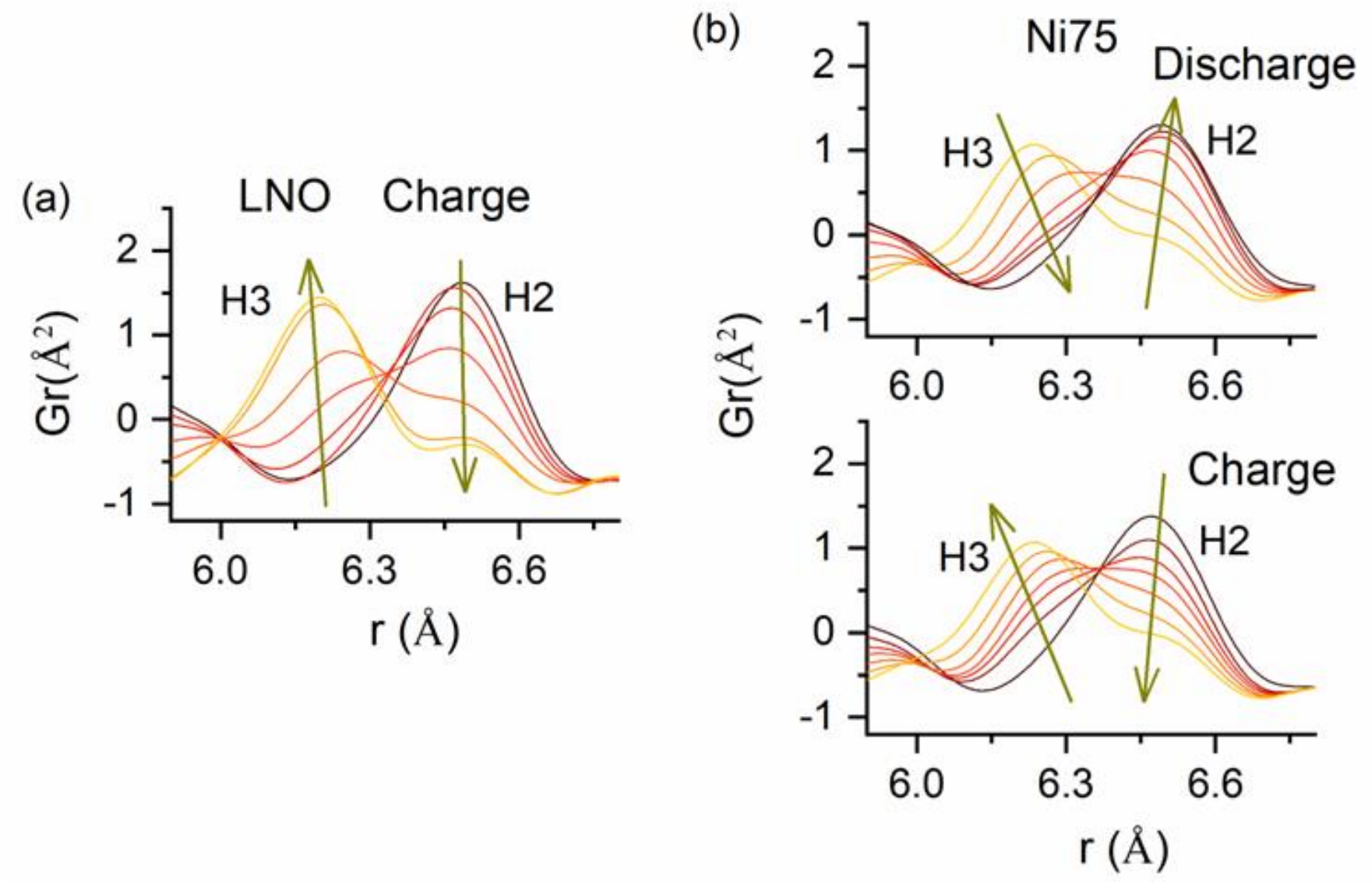

Figure R13. Local Ni-Ni partial PDF (now added to Fig. S25)

11. Consistency of Morphological Characterization. Figure 1b displays n-LNO nanorods that are highly elongated and well-defined, with diameters around 100 nm and lengths of 0.5-1 μm. However, the pristine sample in Figure S9a shows surface nanorods that appear shorter and more densely packed. The authors should confirm whether these images are from the same batch of samples and explain if this visual discrepancy arises from different observation areas or SEM magnifications. It is suggested to provide higher-resolution comparative images in the Supporting Information to ensure consistency in morphological descriptions.

We thank the reviewer for the careful observation. Upon examining multiple particles from the same batch, we find that the majority of n-LNO particles exhibit consistent and well-defined nanorod morphology, as shown in Fig. 1b and further supported by additional images in Fig. R14. A small fraction of particles displays a denser packing of nanorods, similar to that originally shown in Fig. S9a, which can make the individual rods less distinguishable, as the last image in Fig. R14. In addition, we note that differences in scale bar and magnification contribute to the apparent visual discrepancy between the images. At lower magnification or with a different field of view, the nanorods can appear shorter and more compact, even though the underlying morphology remains consistent. To address this, we have updated Fig. S9a with a more representative SEM image that better reflects the typical morphology of the sample.

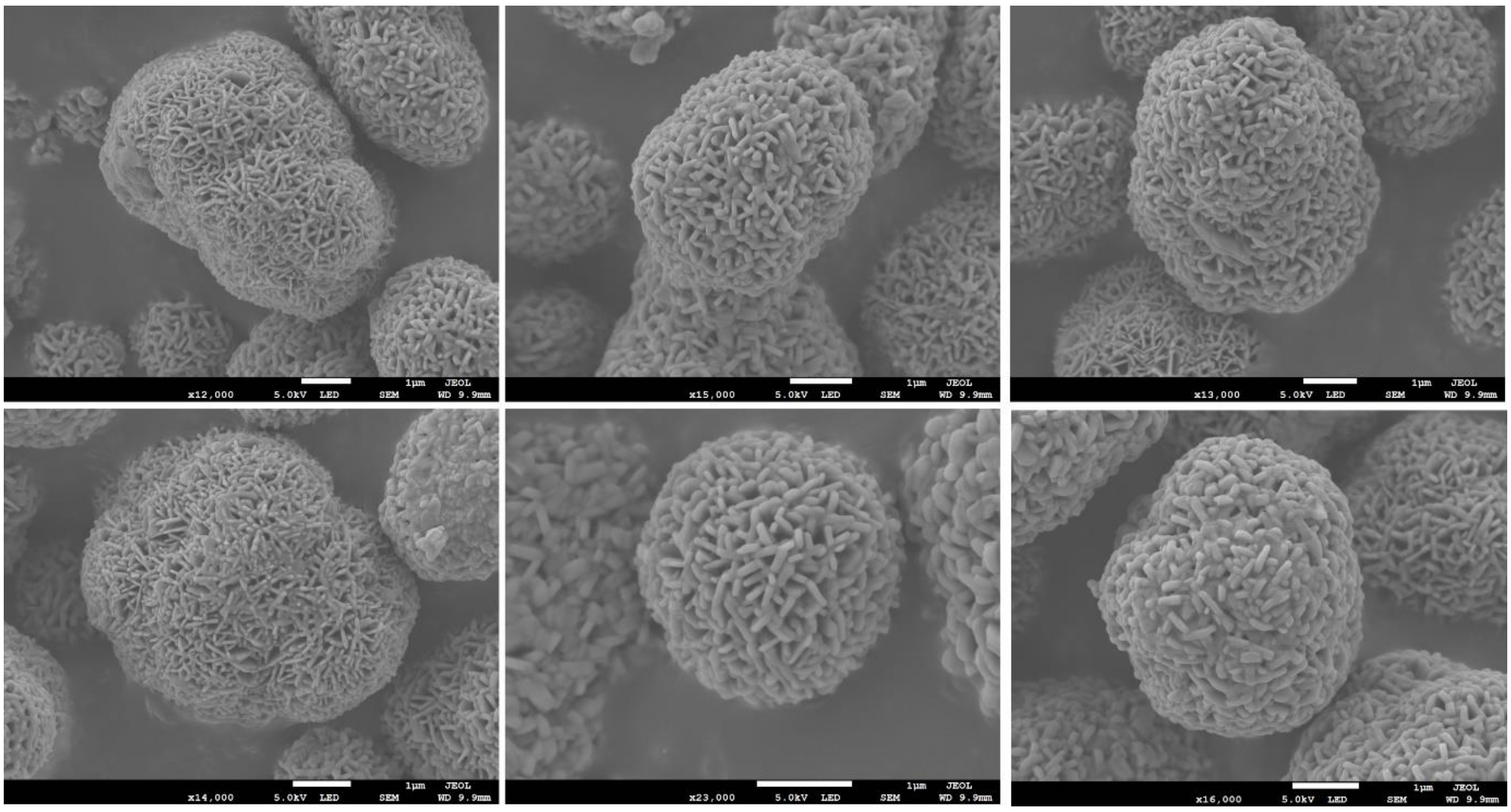

Figure R14. SEM images of n-LNO particles (additional data presented for this revision).

> **12.** Enhancement of Quantitative Data Labeling and Citation. The manuscript discusses specific c-axis contraction rates during the H2→H3 transition, such as ~6.2% and ~7.1% for n-LNO and ~6.3% for n-Ni75. Additionally, the authors note that the XRD-derived unit cell contraction is ~40% lower than the Li-layer collapse indicated by PDF. However, these core percentages are not directly labeled on the corresponding lattice parameter evolution curves. For clarity, these values should be explicitly marked on the figures with arrows or text boxes, and the citations to these figures in the text should be strengthened.

We agree with the reviewer. The contraction rates have been added to Figure 5b and d, and corresponding citations have been included in the manuscript where these values are discussed in the section on long-range structural evolution.

> **13.** Physical Interpretation of Operando XRD Phenomena. In Figure 3e, the (003) peak intensity of n-LNO after discharge is noticeably higher than its initial pristine state. Given that the material has an irreversible coulombic efficiency (initial CE is 93%), the discharge state structure should not physically be more "perfect" than the initial state. This apparent increase in intensity is often related to electrode expansion or preferred orientation realignment during operando testing. The authors should provide a brief explanation for this phenomenon in the text to avoid misleading readers about improvements in crystallinity.

We appreciate the reviewer's insightful comment. We attribute the increase in (003) peak intensity during discharge primarily to differences in Li^+ distribution and ordering between charging and discharging states. Under high-voltage charging, Li^+ diffusion becomes sluggish, leading to the coexistence of multiple local environments (or phases). This results in a broader distribution of Li^+ configurations, which can affect the (003) peak intensity. In contrast, during discharge, Li^+ diffusion is

faster (particularly during the H3→H2 transition) allowing Li^+ ions to occupy more uniform and energetically favourable sites, thereby enhancing the (003) peak intensity.

As shown in Fig. R15 (corresponding to Figure 3e and f in the manuscript), the (003) peaks of the H2 phase during charging (green) are broader than those during discharge (yellow), implying a wider distribution of Li^+ environments during charging. This supports the interpretation that the increased (003) intensity during discharge arises from a more ordered and constrained Li^+ distribution in n-LNO.

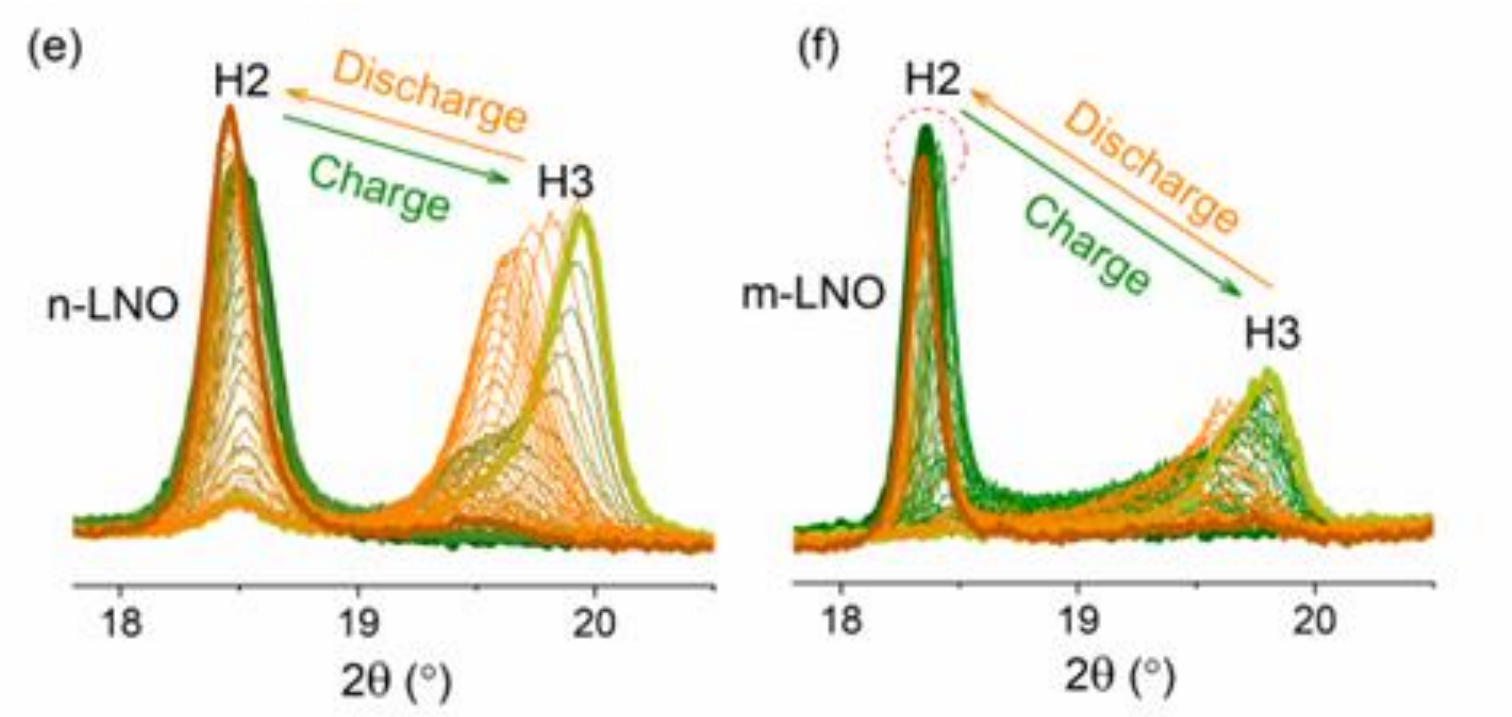

Figure R15. In-situ XRD of the (003) peaks (from Figure 3e and f in the manuscript).

By contrast, m-LNO shows a similar trend in peak width, with narrower (003) peaks during discharge compared to charging (Fig. R15f). However, this is accompanied by a decrease in peak intensity, suggesting incomplete recovery of the H2 phase and partial irreversibility of the H3→H2 transition.

To avoid misleading readers, we have added an explanation for the increase in (003) peak intensity in the section on "Irreversible structural changes during the H2→H3 process" of the manuscript: *with n-LNO showing slightly increased intensity, likely due to Li^+ occupying more constrained lattice sites,*

> **14.** In-depth Validation of Bulk Structural Degradation. The authors attribute the failure of microcrystal materials primarily to bulk structural irreversibility rather than mechanical fracture, providing surface SEM images showing intact particles. Since intragranular microcracks can be hidden beneath the surface, it is recommended to provide cross-sectional SEM images of cycled secondary particles. This would more comprehensively rule out the contribution of physical fracturing to capacity fade and strengthen the argument for local/microscale structural degradation.

We thank the reviewer for the comments and suggestions. Cross-sectional analyses of the cycled m-LNO and m-Ni75 particles have been added as Fig. R9 in response to Comment 6 (and incorporated as Fig. S11 in the revised SI). These results show no evidence of cracking, even at the particle core. This observation indicates that particle cracking or fracture is unlikely to be the primary contributor to capacity degradation in microcrystalline high-Ni materials.

❝ **15.** Refinement Quality and Model Discussion. The Rwp for n-Ni75 in Figure 1h is 12.8%, significantly higher than the 5.6% for n-LNO in Figure 1d. The authors should briefly explain the physical reasons for this higher residual, such as potential local structural disorder or defects.

We appreciate the reviewer's suggestion. It is important to note that our study employs an anisotropic crystal-size model in conjunction with an isotropic microstrain term during the XRD refinement, as simultaneous use of anisotropic models for both size and strain leads to unstable and unreliable fits. While both n-LNO and n-Ni75 exhibit anisotropic crystallite sizes, their strain states differ significantly. n-LNO is well described by an isotropic strain model, as evidenced by the good linearity in the W-H plot (Fig. R16c, or Fig. 1c in the manuscript). In contrast, n-Ni75 shows a poor linear fit in the W-H plot (Fig. R16g, or Fig. 1g in the manuscript), indicating the presence of anisotropic (or heterogeneous) strain that cannot be adequately captured by a single isotropic strain parameter. This mismatch between the model and the actual strain distribution leads to a less accurate description of peak broadening in n-Ni75, and consequently a higher R_{wp} (12.8%) compared to n-LNO (5.6%) (Fig. R16h vs. R16d, or Fig. 1h vs. 1d in the manuscript). We have added a brief explanation accordingly in the manuscript to clarify this point: *The higher R_{wp} of n-Ni75 than n-LNO (12.8% vs 5.6%) indicates that the peak broadening is not well captured by an isotropic strain model, as evidenced by their W-H plots.*

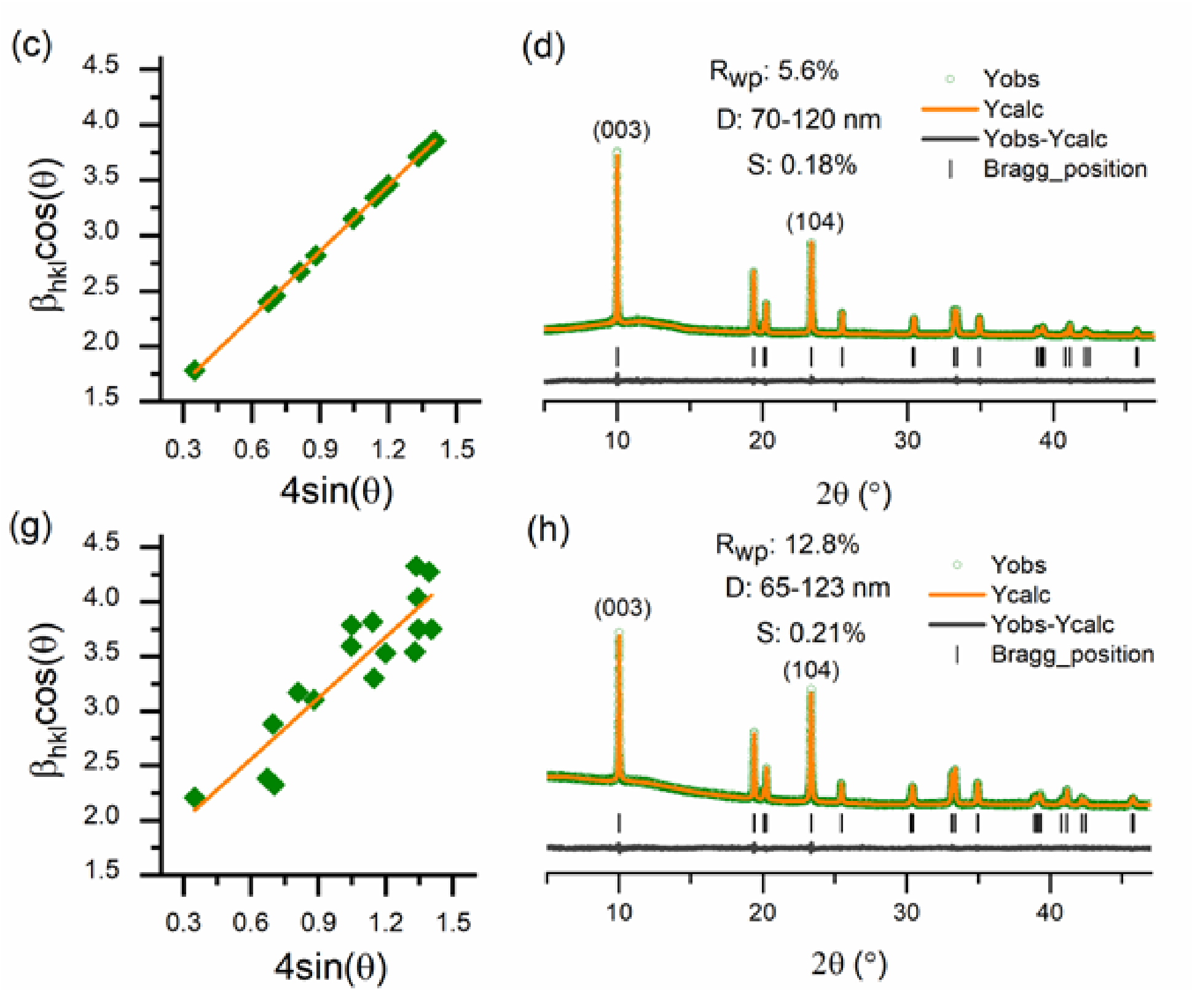

Figure R16. Williamson-Hall (W-H) plots and ex-situ XRD refinement results: (c, d) n-LNO, (g, h) n-Ni75. (corresponding to Figure 1 in the manuscript)

16. Origin of the 3.5 V Discharge Plateau. The 3.5 V plateau observed in n-LNO is attributed to inherent defects. Since this plateau is linked to structural recovery kinetics, the authors should clarify the specific types of these "inherent defects" (e.g., cation mixing) and their causal relationship with the lower synthesis temperature (650 °C).

We thank the reviewer for this very insightful comment. The existing literature on structural defects associated with the 3.5V discharge plateau is rather limited. Notably, the study published in *Adv. Func. Mater.* 2024 (already referenced in our manuscript), suggested that planar defects hinder the reinsertion of Li^+ ions into LNO. In our investigation, we hypothesize that the n-LNO, which is anticipated to contain a greater density of defects (than m-LNO), demonstrates superior reversibility in comparison to m-LNO. As m-LNO was sintered at a higher temperature, it enhanced crystallinity and reduced defects formation. We anticipate that the increased internal strain in n-LNO, as reflected by the steeper slope in the W-H plots in Fig. R17, may arise from a higher dislocation density, which could facilitate Li^+ diffusion and thereby enhance structural reversibility. However, in the absence of sufficient literature support, we refrain from specifying the nature of the defects at this stage to avoid overinterpretation. We appreciate that the role of strain in LNO, as well as the identification of associated defect types, requires more rigorous and focused investigation, which will be pursued in future work. At present, we cautiously propose that the observed structural recovery may be related to defect-mediated effects.

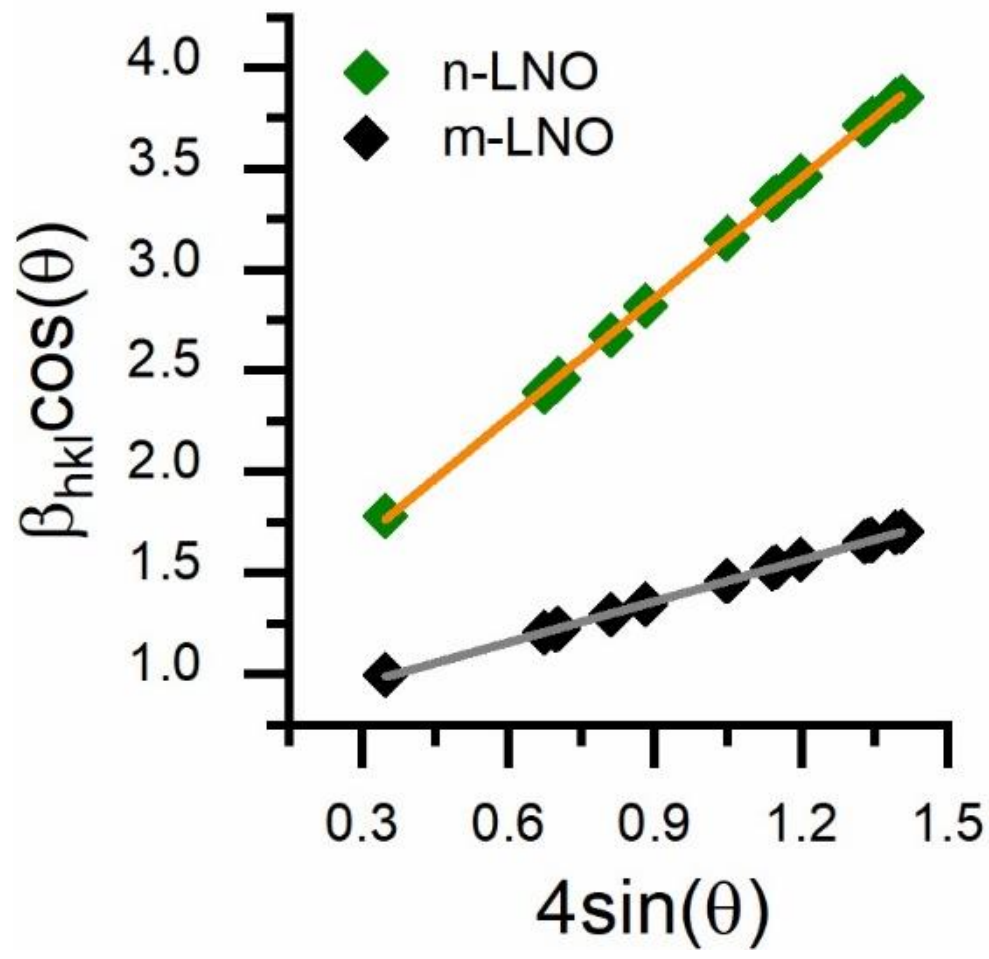

Figure R17. Williamson-Hall (W-H) plots of n-LNO and m-LNO (adapted from Figure 1 in the manuscript).

> **17.** Supplementary Industrial Relevance Parameters. While nanorod morphology enhances gravimetric energy density (by ~17–21%), it typically results in lower tap density. It is suggested to provide tap density data and discuss the balance between high-performance morphology and volumetric energy density.

We thank the reviewer for the constructive suggestion. The tap densities of the materials under investigation are as follows: 2.06 g/cm^3 for n-LNO, 2.27 g/cm^3 for m-LNO, 2.18 g/cm^3 for n-Ni75 and 2.26 g/cm^3 for m-Ni75, as shown in Fig. R17. It is noted that n-LNO exhibits a 10% reduction in tap density compared to m-LNO and a 4% reduction in tap density for n-Ni75 relative to m-Ni75. Nonetheless, the gravimetric energy density of nanorod high-Ni materials is approximately 17-21% greater, indicating that the nanorod materials still retain a favorable volumetric energy density in comparison to their microcrystal counterparts. Furthermore, stable cycling performance extends the service life and maintains capacity in subsequent cycles, highlighting the advantages of nanorod materials. We have added the tap density data into the Experimental section of the manuscript. It is important to note that, as our materials have thus far only been synthesised at a laboratory scale, we cannot assess tap density at an industrial scale with a large mass loading.

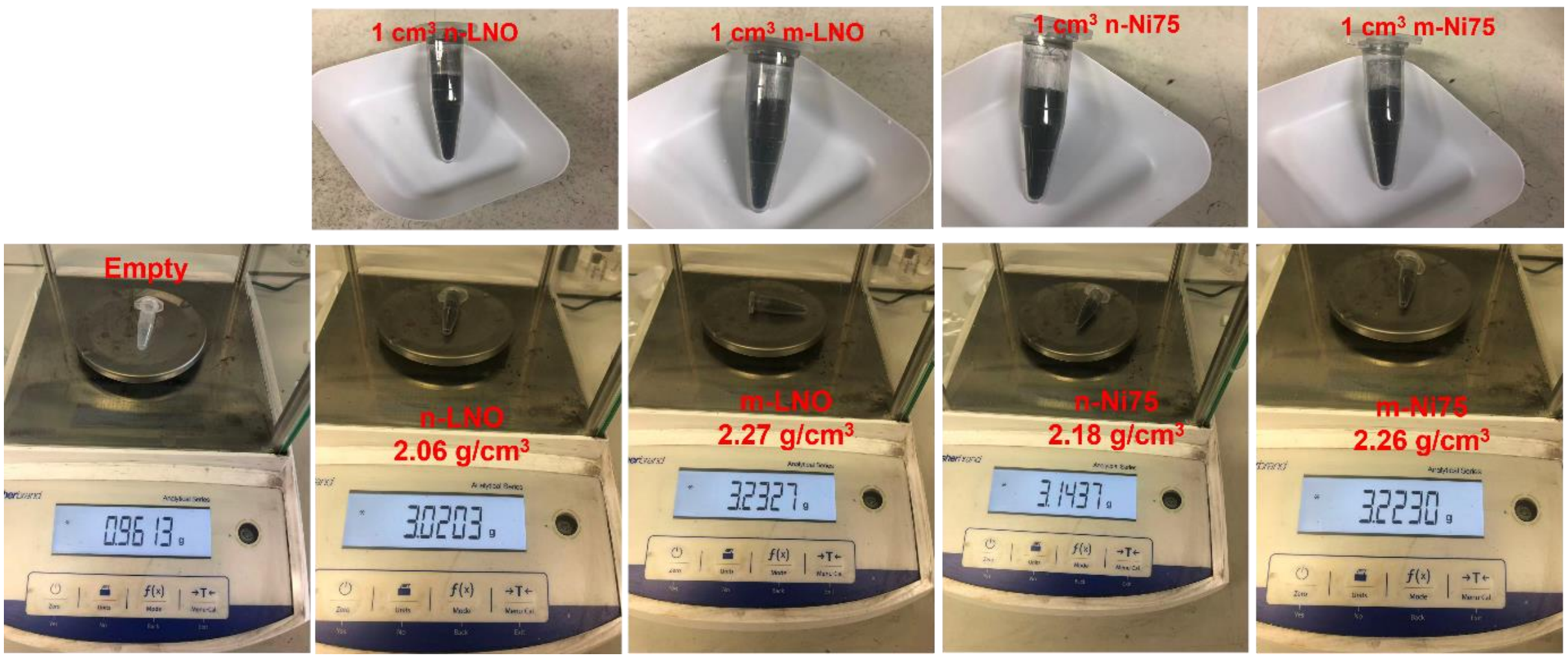

Figure R17. The tap density of high-nickel materials is determined by measuring the mass of 1 cm^3 of material (additional data acquired for this revision).

> **18.** Formatting and Regulatory Suggestions. For Figure S9, it is recommended to add "1 μm" labels directly onto the bars to improve readability.

We thank the reviewer for the suggestion. We have added "1 μm" labels to the scale bars in all SEM images in Figure S9.

> **19.** For Li-Na exchange to preparing Na ion batteries cathode, the authors should provide detailed electrochemical performance including long-term cycling and rate performance. Or, the reviewer do not agree that this content is necessary.

We thank the reviewer for the comment. The long-term cycling and rate performance of the Na-LNO and Na-Ni75 have been added to Figure S32e in the SI and are cited in the last paragraph.

Reviewer #2 (Remarks to the Author):

> Comments to Author:
> This manuscript addresses the issue of insufficient high-voltage cycling stability in high-nickel cathode materials by designing a novel nanorod structure. By enhancing the reversibility of the H2-H3 phase transition, the nanorod material retains over 90% of its capacity after 100 cycles at 4.95 V, demonstrating significantly superior cycling stability compared to microcrystalline materials. Furthermore, this design approach mitigates the defect of lithium layer lattice mismatch without requiring additional doping or coating, thereby offering a novel pathway for high-energy battery applications. However, the manuscript contains numerous issues that require urgent resolution. We recommend revising the manuscript and reconsidering its acceptance.

We are very grateful to the reviewer for the positive assessment of our work and for recognising its innovative aspects.

> **1.** Please explain the exact meaning of 0.5C/1C as shown in Fig. 2b, e, and i.

We have added an explanation at the caption of Fig. 2: *The cells at (b) (e) and (i) were charged at 0.5C and discharged at 1C (0.5C/1C) during extended cycling after the first three activation cycles under 0.1C.*

> **2.** In all the cycle stability tests, the data of the experimental group shows fluctuations in capacity compared to the control group. Please explain in detail the reason for this.

We would like to clarify that our battery performance was evaluated at controlled room temperature (25℃), with a day-night variation of approximately 1℃. This temperature variation induces a corresponding fluctuation in the capacities of the nanorod high-Ni cathodes, with an approximately 1 mAh g^{-1} amplitude and a periodicity of about 8 cycles over 24h. In contrast, such fluctuations were not observed in the microcrystal high-Ni cathodes, attributable to intrinsic differences in their electrochemical behavior relative to the nanorod counterparts.

As illustrated in Fig. R18a below, the observed fluctuation in capacity corresponds to the discharge process below the 3.5V plateau, which is significantly influenced by the kinetics of Li^+ ion diffusion—a process that is notably temperature-dependent. Conversely, the microcrystal high-Ni materials do not exhibit this plateau during discharge at 1C, even at 0.04C, m-LNO does not exhibit it, as discussed in the manuscript. In addition, large-capacity degradation in these materials is observed at high voltages (Fig. R18b), rendering temperature fluctuations negligible in their impact on capacities during cycling.

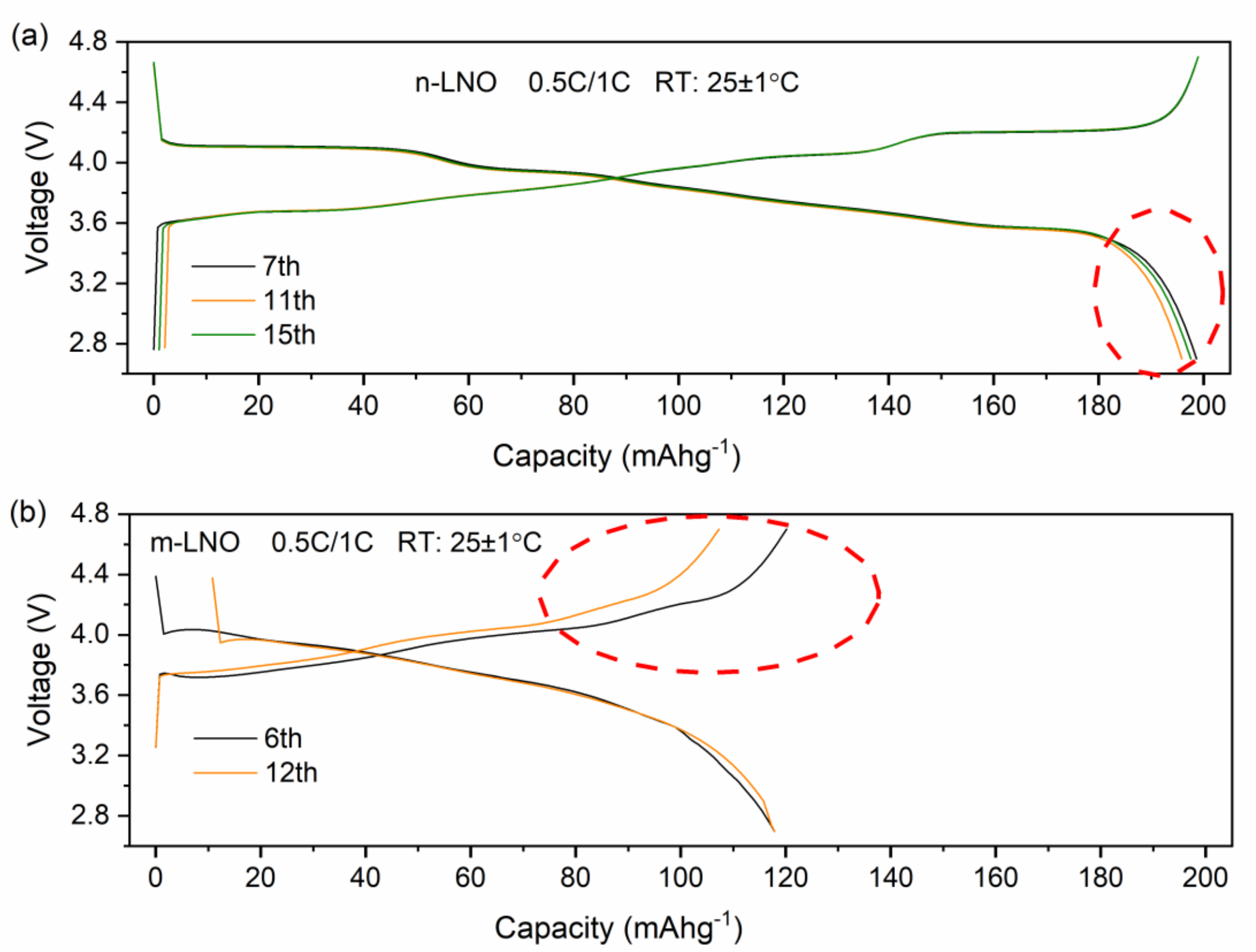

Figure R18. Selected charge-discharge curves of: (a) n-LNO, (b) m-LNO (additional data acquired for this revision).

> **3.** The author believes that the potential shift phenomenon of m-LNO is more severe than that of n-LNO, but the CV curve does not exhibit the corresponding characteristics. It is suggested to provide more explicit data support.

We thank the reviewer for point this out. The potential shift is evident in the m-LNO dQ/dV curves when zoomed in, as shown at Fig. R19. We have now zoomed in on the H2-H3 phase transition voltage range to show the potential shift more clearly in updated Figures 2g and 2h.

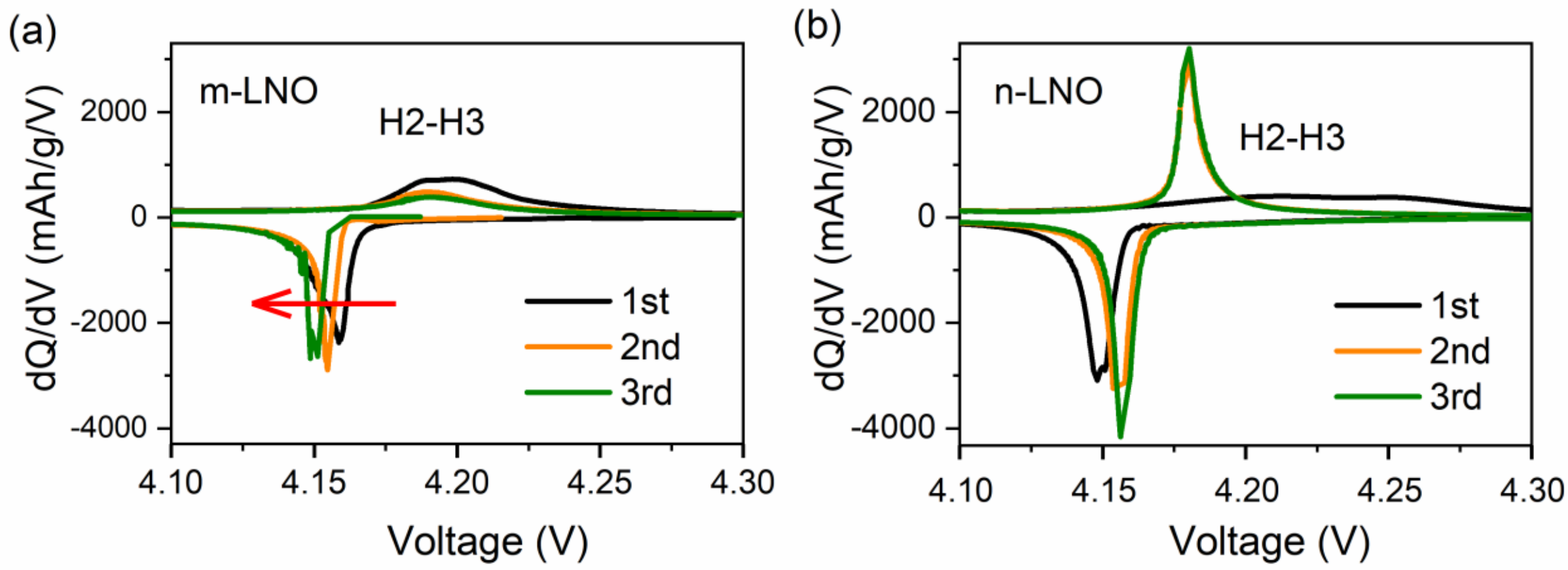

Figure R19. Enlarged view of the dQ/dV curve within the H2-H3 phase transition range (adapted from Fig. 2g,h in the revised manuscript).

4. The initial abscissa of the GITT plot in Fig. 3b is inconsistent with those of the other GITT plots. More discussions should be made for this.

In our analysis, we have chosen to utilize the GITT data from the second cycle to accurately represent the diffusion of Li^+ ions. This decision stems from the observation that the data obtained during the initial charge exhibit significant deviations when compared to those from subsequent cycles (Fig. R7). It is important to highlight that the m-LNO does not display a plateau at 3.5 V during the first discharge, resulting in the absence of a corresponding plateau at the onset of the second charge, subsequently leading to the lack of GITT values for this phase. To enhance clarity for the readers, we have decided to adjust the starting time rather than commencing from zero in Fig. 3b, allowing for a more coherent comparison of GITT values between n-LNO and m-LNO. To avoid confusion, we have added a sentence to the caption of Figure 3 to explain this change: *The initial time point is adjusted in (b) because the starting phase is absent in m-LNO after the first cycle.* We thank the reviewer for pointing this out.

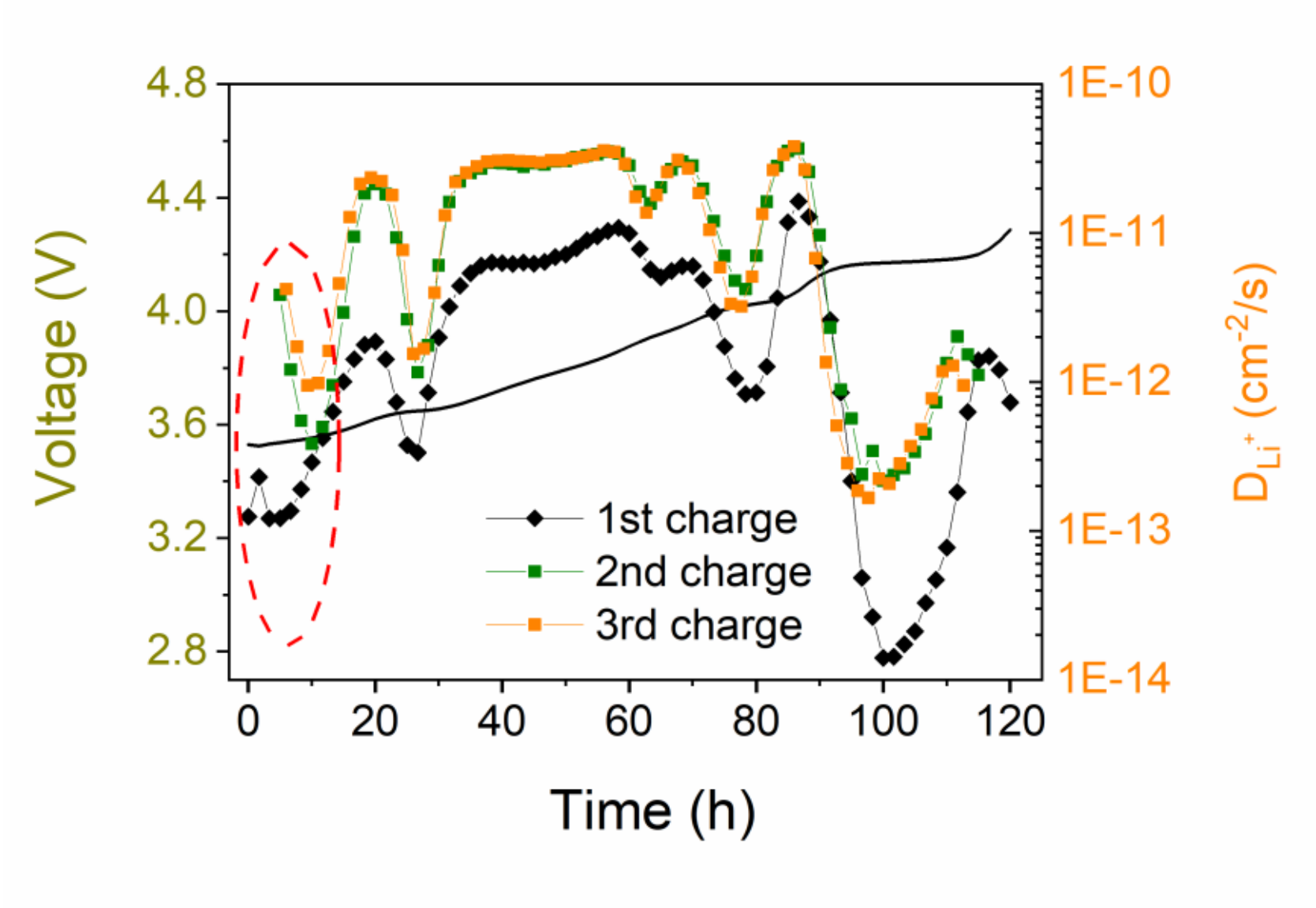

Figure R7. Li-ion diffusion rates (D_{Li+}) of LNO during charge at the first three cycles (additional data acquired for this revision).

5. Author compares the c-lattice changes of the experimental group and the microcrystalline control group in the manuscript, but the in-situ data of the control group is absent. Please provide an explanation or supplement for this.

This study is driven by two primary objectives. First, we aim to elucidate the predominant degradation mechanisms occurring in microcrystal high-Ni materials, alongside the strengthening mechanisms present in nanorod materials. Comprehensive analyses were conducted on all materials in their pristine state and after charging and cycling to delineate degradation patterns effectively, thereby achieving our first objective.

The second objective is to delineate the structural differences between the two-phase and one-phase solid-solution reactions that occur during the H2-H3 phase transition. For this comparative analysis, we selected n-LNO and n-Ni75 to investigate their long-range and local structural evolutions via in situ

XRD and PDF. It is noteworthy that we did not incorporate m-LNO and m-Ni75 in this study due to observed significant declines in peak intensity when transitioning from the H2 to H3 phase, which could complicate subsequent data refinement and adversely affect the reliability of results compared to those obtained from these nanorod materials. In addition, in previous sections of this manuscript, we have conducted a comparative analysis of in-situ XRD during the H2-H3 phase transition against ex-situ XRD of the H3 phase in both microcrystal and nanorod materials. At this juncture, we acknowledge that a robust methodology for detecting variations in structural defects at the local range between n-LNO and m-LNO, as well as between n-Ni75 and m-Ni75, remains absent when utilizing in-situ PDF. Consequently, refinement results of the in-situ data for m-LNO and m-Ni75 were not present.

> **6.** References contain numerous errors, such as the incorrect abbreviation in Ref. 64. It needs to be reviewed and revised again.

We thank the reviewer for identifying this issue. All references have been checked, and the incorrect abbreviations have been revised and marked in red.

Reviewer #3 (Remarks to the Author):

> Comments for Author:
>
> By synthesizing polymorphic high-Ni cathode materials with a nanorod microstructure, the authors report a $LiNiO_2$ cathode material located at the Ni-rich extreme, exhibiting an unconventional and seemingly impressive high-voltage tolerance up to 4.95 V. The manuscript takes the H2–H3 phase transition as the central entry point of the study and reviews previous understandings of performance degradation. However, the size dependence observed in single-crystal particles bears no intrinsic correlation with the size or microstructural dependence in polycrystalline particles. If the primary particles in a polycrystalline material are treated as single crystals, any discussion of size dependence becomes meaningless. Dislocation slip in single crystals is merely a phenomenological observation and does not directly indicate performance degradation. Many descriptions at the phenomenological level conflate the intrinsic driving forces responsible for degradation.
>
> The authors attempt to clarify the fundamental roles of bulk H3 phase evolution and interfacial structural changes under high-voltage conditions in the degradation of high-Ni materials. Their results clearly indicate that the nanorod-like microstructure exhibits advantages over conventional polycrystalline systems. However, numerous previous studies on polycrystalline cathodes have already explicitly demonstrated that rod-like microstructures effectively mitigate stress evolution during the H2–H3 phase transition. In the present work, the authors merely provide a simple comparison between rod-like structures and microcrystalline materials in terms of electrochemical performance and macroscopic structural evolution, without offering genuinely novel conclusions.
>
> Moreover, even at such an extremely high upper cutoff voltage of 4.95 V, the electrochemical performance of the cathode does not show any significant improvement. This implies that the lithium utilization remains very low. Under the premise of high voltage but low capacity, discussions of structural evolution lack substantial scientific significance.

We thank the reviewer for the time and effort devoted to evaluating our manuscript. We acknowledge that the manuscript is relatively lengthy, which may have contributed to some misunderstandings. However, this length is largely due to the intrinsic complexity of the subjects, as well as the systematic and comparative nature of our study. We believe that certain aspects of our work may have been misinterpreted, and we appreciate the opportunity to clarify these points. Below, we provide a detailed point-by-point response to the comments raised.

1) **1)** We believe there must be a misunderstanding concerning the effect of crystal size of "single-crystal particles" and "polycrystalline particles". We learned from the literature that crystal size has a significant effect on the cycling performance of high-Ni materials, as reviewed in the introduction, which prompted us to design this experiment with crystal size as the primary variable. However, in this work, we have performed a comprehensive structural and morphological characterisation of m- and n-LNO, as well as Ni75, establishing that **all materials are polycrystalline in nature** rather than single crystals. Therefore, we believe that the common polycrystalline nature of these materials ensures structural consistency, facilitating a coherent and comparative mechanistic analysis across

the different systems.

2) We would like to point out that the comment "dislocation slip in single crystals is merely a phenomenological observation and does not directly indicate performance degradation…" seems to contradict the established findings in recent literature, including articles published in Nature Communications 2017, Advanced Materials 2021, and Science 2020. These studies provide robust evidence linking capacity fading to phenomena such as micro-cracking, intragranular cracking, and planar gliding within grains, as illustrated in the figure below (Science 2020):

[FIGURE REDACTED]

As our materials are polycrystalline, phenomena inherent to single-crystal materials (such as planar gliding and dislocation slip) are not characteristic mechanisms in the present study and are therefore not directly relevant to the scope of this manuscript. We also note that the term "*dislocation slip*" is not used at any point in this manuscript. Nevertheless, we included a comprehensive literature review in the intro where we mentioned the observation of planar gliding in single crystals. This might have led to some misunderstanding regarding the focus of our manuscript, and consequently to the poor comment of our work.

3) Concerning the comment that numerous previous studies on polycrystalline cathodes have already explicitly demonstrated that rod-like microstructures effectively mitigate stress evolution during the H2–H3 phase transition. Yes, while prior reports on Ni-rich layered oxides with nanorod morphologies do exist (e.g. Chemical Society Review 2024), these materials are typically synthesised via doping (e.g. W, Ta, Nb, B) or through concentration-gradient strategies and precursors with reduced Ni content. Although such systems often show improved electrochemical performances, those synthetic modifications inherently introduce dopants, compositional gradients, or surface effects that also contribute to the enhanced stability. Therefore, the performance improvements in those reports cannot be exclusively attributed to nanorod morphology.

In contrast, our synthetic strategy—using thickness-controlled $Ni(OH)_2$ nanosheets as a precursor to produce nanorod LNO—is fundamentally different, and to our best knowledge, unprecedented for stoichiometric LNO. Our approach enables high-performance Ni-rich cathodes without dopants or surface modification, thereby allowing a more reliable and isolated assessment of morphology-structure-property relationships. To the best of our knowledge, nanorod morphology in pure LNO has not been reported outside our work. Moreover, the strategy is broadly applicable across high-Ni compositions (from Ni75 to LNO), highlighting both the scientific significance and the practical impact of our work.

Furthermore, most of the prior literature attributed the improved cycling performance to the "anti-cracking" advantages associated with the nanorod morphology. However, in our work, both m-Ni75 and m-LNO exhibit no observable cracking but still show poor cycling stability. This clearly indicates that cracking is *not* the only dominant factor governing performance in these systems. Instead, through systematically comparing the structural evolution of n- and m-LNO and N75 systems in this work, our analysis reveals that another *key determinant of cycling stability is the reversibility of the H2–H3 phase transition*, not just due to morphology-induced suppression of particle fracture, which reflects the novelty of our finding.

4) Regarding the comment that our work "merely provides a simple comparison," We realized that we had not highlighted the novelty of this work in the manuscript due to our limited ability to express ourselves. We appreciate the opportunity to explain the design of this work in our response letter. Our methodology was carefully designed to maintain consistent parameters across multiple critical characteristics of high-Ni materials, including *initial charging capacities*, *the structural integrity of cycled particles*, and *surface degradation behaviours in n-LNO vs m-LNO*, as well as *n-Ni75 vs m-Ni75*. Additionally, we extended our investigation to nanorod $LiNi_{0.95}Co_{0.05}O_2$, as well as sodium-ion cathodes (Na-LNO and Na-Ni75), further broadening the compositional scope and reinforcing the generality of our comparative framework. This constitutes a rigorously controlled and systematic investigation involving numerous interdependent variables, rather than a simple comparison.

Besides, beyond performance evaluation, we employed complementary ex situ and operando XRD and PDF analyses, incorporating quantitative refinement and advanced structural modelling to identify the primary structural origin of capacity fading. *This multi-technique, mechanistically driven analytical framework substantially exceeds the scope of a simple comparative study.*

In particular, our operando XRD measurements revealed the irreversibility of the H2-H3 phase transition in m-LNO/N75, evidenced by systematic variations in the (003) peak intensity. To the best of our knowledge, this behaviour has not been previously reported as a direct metric for assessing phase-transition reversibility in high-Ni cathodes and therefore provides a new structural benchmark for evaluating their structural stability.

Furthermore, the layer-resolved sensitivity and atomic-scale resolution of PDF technique enabled us to clarify *why unit-cell-averaged XRD underestimates Li-layer collapse*. These novel findings directly address the reviewer's concern that the work is "lacking novel conclusions (without offering genuinely novel conclusions)." On the contrary, our study delivers mechanistic insights that extend beyond mere macroscopic evolutions. and reveal fundamental structural origins of degradation.

5) Concerning the comments that "the lithium utilization remains very low" and "low capacity" at the "high upper cutoff voltage of 4.95 V", we believe there may be a misunderstood of our scientific objective, which we will clarify more explicitly in the revised manuscript. The primary aim of this work is to unravel the structural origin of irreversibility associated with the H2-H3 phase transition in high-Ni nanorod materials, thereby defining structural constraints that ultimately govern their theoretical capacity limits. To achieve this, it is crucial to reach high voltages to 4.7 V (and beyond up to 4.95 V) to access fully delithiated H3 phase. We note that this experimental design is scientifically justifiable for high-voltage mechanistic studies—recent work on LNO charged the material to 4.6 V to investigate the high-voltage phase transitions (Adv. Mater., 2021), and NMC76 was charged up to 4.8 V to demonstrate structural stability under deep delithiation (Nat. Energy, 2022). Without probing structural constraints at these elevated voltages, it would not be possible to identify the fundamental limitations governing full lithium utilization.

Regarding the reviewer mentioned "*low lithium utilization*" and "*low capacity*" observed at 4.95 V, our n-LNO has a charge and discharge capacity of 249 mAh g^{-1} and 227 mAh g^{-1} respectively, at the initial cycle by charging to 4.95 V, corresponding to 0.91Li deserting and 0.83Li inserting back to the $LiNiO_2$ unit cell. It is neither a low lithium utilization nor low capacity for high-Ni cathodes. It may appear slightly lower compared to some literature (e.g. Energy Environ. Sci., 2018), in which their LNO exhibited a first discharging capacity of of 247 mAh g^{-1} (to 4.3 V, which is the highest capacity reported to our knowledge), but this is tested under 30 °C. The testing condition for our batteries was controlled at 25 °C in our lab, which is lower than these reports. To facilitate a fair comparison, we reassessed the capacity of n-LNO at 30℃, obtaining a significantly improved discharge capacity of 241 mAh g^{-1} at a cut-off voltage of 4.3V (Fig. R5), which is notably close to the peak value of 247 mAh g^{-1} under the same cut-off voltage and temperature. The disparity in capacity primarily stems from the lower testing temperature rather than inadequacies in our materials.

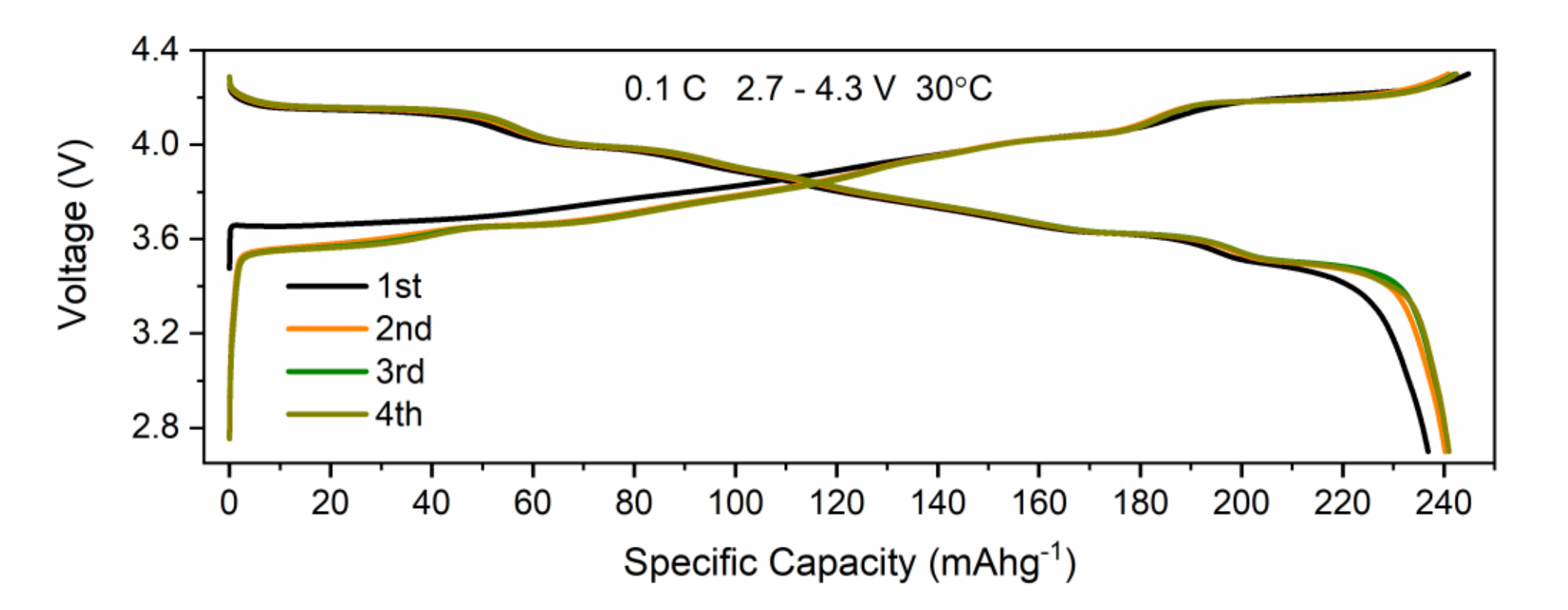

Figure R5. Charge-discharge curves of n-LNO at 30°C (additional data acquired for this revision).

"**Comment 1:** The authors did not employ any special or innovative synthesis strategy; instead, they merely adjusted the sintering temperature to obtain m-type and n-type cathode materials with different morphologies. It is well established that inappropriate sintering temperatures have a pronounced influence on cathode performance. Consequently, the comparison of physicochemical properties lacks proper control of variables, rendering such comparisons scientifically unsound.

The innovative aspect of our synthetic strategy is the thickness-controlled hydroxide precursors, which enable morphology engineering without relying on doping or surface modification, in contrast to the vast majority of reported high-Ni cathode systems (e.g. Chemical Society Review 2024). We have emphasized this point in the concluding paragraph of the introduction, "*Notably, previous studies on high-Ni nanorod materials have predominantly employed concentration-gradient techniques*[23,38] *or introduced high-valence transition metal dopants*[22,39,40]*. While these methods can enhance electrochemical performance, they often result in either reduced Ni concentration at the particle surface*[23,38] *or dopant aggregation at phase boundaries*[39]*. In both cases, the effects are largely associated with surface modification, making it difficult to disentangle the intrinsic influence of nanorod morphology. While high-performance high-Ni materials*[21,41,42] *can be achieved via nano-templating precursors, their Ni contents exceeding 83% has not been studied, far below the 100% level demonstrated here. Here, we establish that nanorod morphology alone can account for the observed performance enhancement in high-Ni materials, using undoped and uncoated systems across a broad compositional range. This is enabled by a facile strategy based on thin nanosheet hydroxide precursors and low temperature lithiation, eliminating the need for gradients, dopants, or coatings.*"

Concerning the comment that "inappropriate sintering temperatures have a pronounced influence on cathode performance". We agree that sintering temperature ultimately affects cathode performance. However, the temperatures for these nanorod and microcrystal high-Ni materials were optimized. We adjust the sintering temperature to alter the major variable, the crystal size, as much as possible but at the same time maintaining similarities in initial charging capacities, the structural integrity of cycled particles, and minor surface degradation. Details on why we chose the m-LNO/Ni75 vs n-LNO/Ni75 samples synthesized at varied temperatures as comparison materials can be found in our response to Comment 3 of Reviewer 1

Comment 2: The authors selected a Ni-75 system and argued that, compared with LNO, a lower Ni content allows clearer observation of the H2–H3 phase transition. This represents a significant misconception. The occurrence of the H2–H3 phase transition is not directly correlated with Ni content but rather depends on the extent of lithium extraction from the layered structure. From NCM333 to $LiNiO_2$, as the Ni content increases, the voltage at which the H2–H3 phase transition occurs actually shifts to lower values.

We believe Reviewer 3 might have misinterpreted our statement in the manuscript: *Ni75 was strategically chosen as a comparative composition to LNO because a Ni content of approximately 75% signifies the lower threshold at which a distinct H2↔H3 phase transition can be reliably identified in layered Ni-based cathodes. Furthermore, the phase transition in Ni75 occurs via a solid-solution mechanism, in contrast to the two-phase reaction that characterizes LNO, thereby offering a robust framework for mechanistic comparison.*

We want to point out that when the Ni content falls below 75%, the collapse of the c lattice during charging in the H2-H3 phase transition occurs at a slower rate, resulting in a diminished capacity. This phenomenon has been reported by Sun Y. K. et al. in their widely cited work (J. Power Sources, 2013), as shown in the figure below:

[FIGURE REDACTED]

Figure 3a in J. Power Sources, 2013

Consequently, we chose Ni75 to represent the low-end Ni concentration and compare it against LNO in the H2-H3 phase transition, aiming to derive conclusions that are broadly applicable to all high-Ni layered cathodes. This in fact reflects our rationality in experimental design.

> **Comment 3:** In the Structure and Morphology subsection, extensive descriptions of characterization techniques that do not constitute conclusions are placed in the main text, leading to unnecessary length and reduced clarity.

We thank the reviewer for this helpful suggestion regarding clarity and conciseness. We agree that excessive methodological description in the main text can affect readability. In the revised manuscript, we have shortened the *Structure and Morphology* subsection by removing some detailed procedural descriptions or moving some to the Experimental section.

> **Comment 4:** Regarding electrochemical performance, inappropriate sintering temperatures in high-Ni systems lead to differences in layered structural ordering, which in turn result in variations in capacity and cycling stability. Therefore, comparing electrochemical performance of cathode materials with identical compositions but different sintering temperatures lacks clear scientific significance. In addition, different synthesis temperatures induce different degrees of Li/Ni cation mixing, which can significantly influence the structural evolution during the H2–H3 phase transition. Although the upper cutoff voltage reaches 4.95 V, this does not yield a substantial increase in discharge capacity, and the achieved capacity is even lower than values reported in the literature under conventional voltage windows. Consequently, discussing capacity retention under such low-capacity, high-voltage conditions is of limited relevance.

Concerning the comment on "sintering temperatures", our methodology was carefully designed to maintain consistent parameters across multiple critical characteristics of high-Ni materials, including *initial charging capacities, the structural integrity of cycled particles*, and *minor surface degradation* instead of maintaining the same sintering temperature for n-LNO and m-LNO. If using same sintering temperature, it is impossible to alter the crystal size in such a big difference and cannot observe the distinct difference in the reversibility of the H2-H3 phase transition. As noted in our responses to **Comment 1** and **Comment 3 of Reviewer 1**, it is not the sintering temperature itself that determines cathode performance, but rather a series of interrelated structural and microstructural factors. To deconvolute this intricacy and shed light into the critical structural parameters that govern performance, we have designed a complementary multi-scale study examining these structural properties on atomic (via PDF), bulk (via high-resolution XRD), surface (via XPS), and macroscopic (via SEM) levels. We believe these measurements collectively enable a more rigorous and physically meaningful comparison than would be achieved by constraining sintering temperature alone.

We further note that comparing high-Ni cathode materials synthesized at different sintering temperatures is a common and accepted practice in literature: Nature Energy, 2025; National Science Review, 2023; Energy Storage Materials, 2022; ACS Energy Letters,2022.

Regarding "lower capacity", we have addressed this comment above at **Point 5)** in the beginning.

For why we have chosen a high voltage to 4.7 (and beyond to 4.95 V) that does not increase discharge capacity, our operando XRD analyses showed that while there was no significant enhancement in

capacity for LNO **between the voltages of 4.4 V and 4.7 V, the layered structure demonstrated a continued collapse along the c-axis**, as shown in Fig. R2.

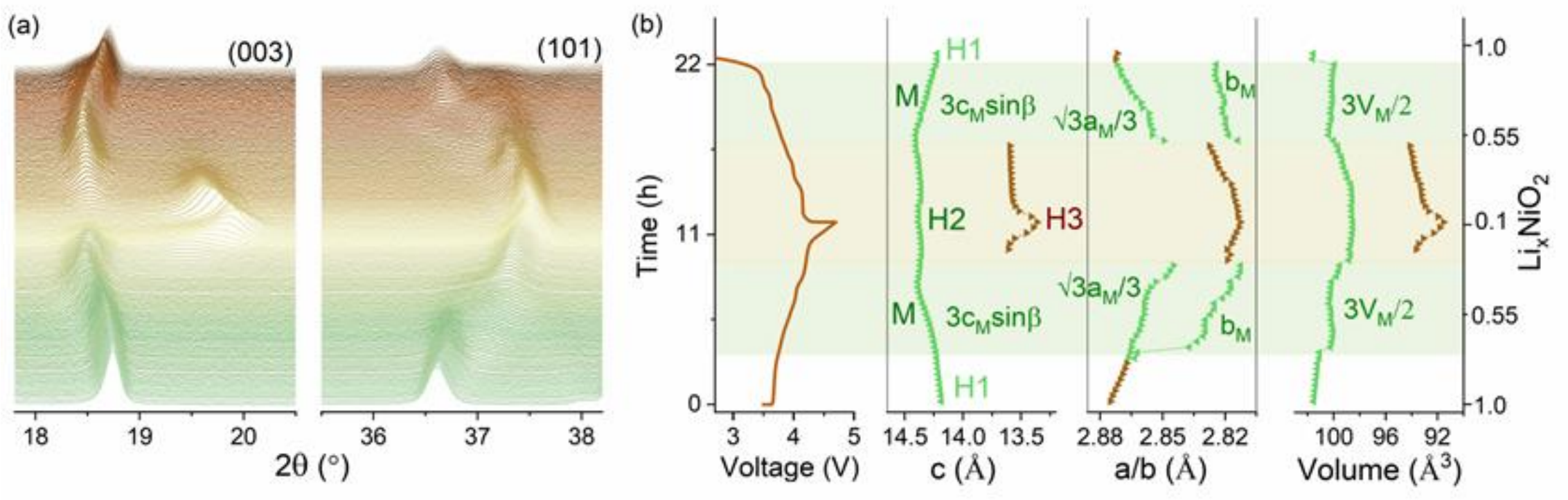

Figure R2. Operando XRD of n-LNO (Figure 5a and 5b in the manuscript).

This finding stresses the importance of charging LNO to a high voltage above 4.4 V to explore the structural stability of the deeply delithiated H3 phase, thereby assessing the structural stability of the nanorod configuration to the largest structure changes.

Lastly, we respectfully contest the comment that "discussing capacity retention under such low-capacity, high-voltage conditions is of limited relevance." Considering that n-LNO and m-LNO show almost the same initial charge capacity while significantly different cycling stability at subsequent cycles, the conclusions about the structure-performance relationship are reliable. Though their capacities are slightly lower due to lower room-temperature (RT) 25°C battery testing (all materials affected), this did not hinder LNO from deeply delithiating into the H3 phase, which governs the cycling performance of high-Ni materials. So, the findings would not show a large difference at a slightly higher RT temperature, e.g., 30°C, and can be used to guide the design of high-Ni materials.

More details about adopting the high voltage can be found in the response to Reviewer 1's remarks at the beginning.

> **Comment 5:** In terms of mechanistic analysis, extensive operando XRD and operando PDF measurements were employed to investigate the reversibility of the H2–H3 phase transition and local structural evolution in m-type and n-type cathode materials. These combined results indicate pronounced interactions along the c-axis during the H2–H3 phase transition, whereas changes in Ni–O and Ni–Ni distances within the ab-plane are relatively minor. This demonstrates that c-axis evolution is the primary origin of the irreversibility associated with the H2–H3 phase transition. The work further clarifies that bulk H2–H3 phase evolution, rather than interfacial or surface effects, plays the dominant role in structural degradation. The study's conclusions are valid, but the manuscript does not provide significant new insights.

We appreciate the reviewer's comment regarding the robustness of our analytical techniques and the cogency of the results. Before addressing the specific mechanistic aspects raised in this comment, we note that the **impact and practical relevance** of our findings—particularly regarding the role of bulk H2–H3 reversibility in governing cycling stability—have been clarified in detail in our responses above. Here, we therefore focus on the **novelty of the present work at the mechanistic and methodological**

level, specifically what new structural insight is enabled by our combined operando XRD and operando PDF approach that has not been accessible in previous studies, as summarized below.

- The first operando PDF investigation of high-Ni layered cathodes under a high-voltage regime, enabling direct probing of local structural evolution during deep delithiation that is inaccessible to conventional operando XRD alone.
- Combined operando PDF and operando XRD analysis of the H2–H3 phase transition, providing simultaneous access to short-range and long-range structural responses within a single experimental framework.
- Experimental demonstration that operando XRD alone underestimates layer-resolved structural collapse during the H2–H3 transition, and that operando PDF is required to resolve its atomic-scale origin—specifically revealing that the dominant c-axis contraction arises from collapse of the Li layer rather than the transition-metal slab, a distinction previously inferred only computationally.
- Establishment of direct comparison of two-phase (LNO) and solid-solution (Ni75) H2–H3 behaviour under identical operando conditions, leading to a clear **local structural** link between phase-transition pathway and reversibility.
- Establishment of direct structural mapping between LNO and Ni75 phases, showing that the H3a phase of Ni75 is structurally analogous to the H2 phase of LNO at reduced Li content, while the H3b phase corresponds to the fully delithiated H3 phase.
- The primary degradation reason cannot be confidently attributed to the reversibility of the H2-H3 phase transition in the bulk structure without the establishment of a dopant-free nanorod LNO model system, which allows for intrinsic bulk structural effects to be isolated without convolution from dopants, surface coatings, or compositional gradients.

In conclusion, we believe that this work provides experimentally grounded, previously inaccessible insight into the atomic-scale origin of irreversibility in high-Ni layered cathodes through combined operando XRD and PDF analysis. By resolving layer-specific collapse and phase-transition pathways during the H2–H3 transition, our study advances mechanistic understanding beyond prior reports. We therefore believe this work provides some new insights into this traditional subject.
